# Low-Dose Methotrexate and Serious Adverse Events Among Older Adults With Chronic Kidney Disease

**DOI:** 10.1001/jamanetworkopen.2023.45132

**Published:** 2023-11-27

**Authors:** Flory T. Muanda, Peter G. Blake, Matthew A. Weir, Fatemeh Ahmadi, Eric McArthur, Jessica M. Sontrop, Brad L. Urquhart, Richard B. Kim, Amit X. Garg

**Affiliations:** 1 ICES Western, London, Ontario, Canada; 2 Department of Physiology and Pharmacology, Western University, London, Ontario, Canada; 3 Department of Epidemiology and Biostatistics, Western University, London, Ontario, Canada; 4 Division of Nephrology, Department of Medicine, Western University, London, Ontario, Canada; 5 Lawson Health Research Institute, London Health Sciences Centre, London, Ontario, Canada; 6 Division of Clinical Pharmacology, Department of Medicine, Western University, London, Ontario, Canada

## Abstract

**Importance:**

Low-dose methotrexate is used to treat rheumatoid arthritis and psoriasis. Due to its kidney elimination, better evidence is needed to inform its safety in adults with chronic kidney disease (CKD).

**Objectives:**

To compare the 90-day risk of serious adverse events among adults with CKD who started low-dose methotrexate vs those who started hydroxychloroquine and to compare the risk of serious adverse events among adults with CKD starting 2 distinct doses of methotrexate vs those starting hydroxychloroquine.

**Design, Setting, and Participants:**

This retrospective, population-based, new-user cohort study was conducted in Ontario, Canada (2008-2021) using linked administrative health care data. Adults aged 66 years or older with CKD (defined as an estimated glomerular filtration rate [eGFR] <60 mL/min/1.73 m^2^ but not receiving dialysis) who started low-dose methotrexate (n = 2309) were matched 1:1 with those who started hydroxychloroquine.

**Exposure:**

Low-dose methotrexate (5-35 mg/wk) vs hydroxychloroquine (200-400 mg/d).

**Main Outcome and Measure:**

The primary outcome was a composite of serious adverse events: a hospital visit with myelosuppression, sepsis, pneumotoxic effects, or hepatotoxic effects within 90 days of starting the study drug. Prespecified subgroup analyses were conducted by eGFR category. Propensity score matching was used to balance comparison groups on indicators of baseline health. Risk ratios (RRs) were obtained using modified Poisson regression, and risk differences (RDs) using binomial regression.

**Results:**

In a propensity score–matched cohort of 4618 adults with CKD (3192 [69%] women; median [IQR] age, 76 [71-82] years), the primary outcome was higher in patients who started low-dose methotrexate vs those who started hydroxychloroquine (82 of 2309 [3.55%] vs 40 of 2309 [1.73%]; RR, 2.05 (95% CI, 1.42-2.96); RD, 1.82% [95% CI, 0.91%-2.73%]). In subgroup analysis, the risks increased progressively at lower eGFR (eg, eGFR <45 mL/min/1.73 m^2^: RR, 2.79 [95% CI, 1.51-5.13]). In the secondary comparison with hydroxychloroquine, methotrexate users at 15 to 35 mg/wk had a higher risk of the primary outcome.

**Conclusions and Relevance:**

In this cohort of 4618 older patients with CKD, the 90-day risk of serious adverse events was higher among those who started low-dose methotrexate than those who started hydroxychloroquine. If verified, these risks should be balanced against the benefits of low-dose methotrexate use.

## Key Points

**Question:**

Is there a higher 90-day risk of serious adverse events among adults with chronic kidney disease (CKD) who start low-dose methotrexate vs hydroxychloroquine?

**Findings:**

In this cohort study that included 4618 propensity-matched adults with CKD, the 90-day risk of serious adverse events among those who started low-dose methotrexate vs hydroxychloroquine was 3.55% vs 1.73%, a statistically significant difference.

**Meaning:**

In this study, adults with CKD who started low-dose methotrexate had a higher 90-day risk of serious adverse events compared with those who started hydroxychloroquine.

## Teaser

This cohort study compares the 90-day risk of serious adverse events among adults with chronic kidney disease (CKD) who started low-dose methotrexate vs those who started hydroxychloroquine.

## Introduction

Low-dose methotrexate is a disease-modifying antirheumatic drug (DMARD) primarily used for the treatment of rheumatoid arthritis and psoriasis.^1,2^ It is also prescribed as an immunomodulator for patients with Crohn disease.^3^ The usual dose of methotrexate for these conditions is 5 to 25 mg/wk,^1,2,4^ and occasionally, the maximum weekly dose is increased to 35 mg/wk.^1,2,4^ Low-dose methotrexate is also prescribed off-label for dermatomyositis, eczema, systemic lupus erythematosus, and systemic sclerosis.^5^ In the United States, 5.9 million methotrexate prescriptions were dispensed in 2019.^6^

Methotrexate is primarily eliminated by the kidneys, with 80% to 90% excreted unchanged in the urine.^5,7^ To avoid toxic effects, product monographs and prescribing guidelines recommend that methotrexate be started at a low dose in patients with chronic kidney disease (CKD) (eTable 1 in Supplement 1). Recommended dose adjustments in CKD are largely based on clinical experience (eTable 1 in Supplement 1). A pharmacokinetic study showed that methotrexate clearance was slower in patients with lower levels of creatinine clearance, and its half-life was 2-fold higher in patients with a creatinine clearance less than 45 mL/min compared with those with a creatinine clearance greater than 80 mL/min (eTable 2 in Supplement 1).^8^

Serious adverse events associated with low-dose methotrexate use include myelosuppression, serious infections, and hepatotoxic effects.^9^ While these risks have been reported in several randomized clinical trials,^10,11,12,13^ there is limited information on whether these risks are amplified in patients with CKD. At least 50 case reports and 2 observational studies (one retrospective cohort study and one observational study using a spontaneous reporting system) of patients with CKD suggest that the risk of toxic effects with low-dose methotrexate (5-35 mg/wk) is substantial (literature search and summary of studies appear in eTables 3 and 4 in Supplement 1). In one cohort study of 120 patients with CKD, almost one-third developed toxic effects after starting methotrexate for rheumatoid arthritis (35 of 120 [29.2%]). This proportion increased in a stepwise manner across categories of patients with lower kidney function (*P* = .02). In another study of 88 patients with rheumatoid arthritis who started treatment with low-dose methotrexate, 3 times as many patients with CKD developed hematological toxic effects than those without CKD (33 of 88 [37.5%] vs 594 of 5560 [10.7%]; *P* < .001) (eTable 4 in Supplement 1).

We conducted a population-based study to examine the risk of serious adverse events in older adults with CKD who started low-dose methotrexate. The primary objective was to assess the 90-day risk of a hospital visit with myelosuppression, sepsis, pneumotoxic effects, or hepatotoxic effects in older adults with CKD who started methotrexate at 5 to 35 mg/wk compared with those who started hydroxychloroquine (200 to 400 mg/d). The secondary objective was to separately examine the risk of toxic effects in patients with CKD starting 2 different doses of methotrexate (5 to <15 mg/wk and 15 to 35 mg/wk) compared with those starting hydroxychloroquine.

## Methods

### Study Design and Setting

This study was conducted using linked administrative health care databases in the province of Ontario, Canada (2008-2021). All Ontario residents (approximately 15 million) have universal access to hospital care and physician services through a government-funded single-payer system.^14^ Those aged 65 years and older (approximately 2.2 million) also receive universal prescription drug coverage. The use of data in this study was authorized under section 45 of Ontario’s Personal Health Information Protection Act, which does not require review by a Research Ethics Board or informed consent from participants. Study reporting follows recommended guidelines for pharmaco-epidemiological studies that use routinely collected health data.^15^

### Data Sources

Data for this study were obtained from 8 health care databases housed at ICES.^16^ The following data sets were linked using unique encoded identifiers and analyzed at ICES: the Canadian Institute for Health Information Discharge Abstract Database, the ICES-derived Physician Database, the National Ambulatory Care Reporting System database, the Ontario Drug Benefit Database, the Ontario Health Insurance Plan database, the Ontario Laboratories Information System database, the Ontario Mental Health Reporting System Database, and the Registered Persons Database. Data on hospital admissions and diagnoses were coded by trained personnel using the *International Statistical Classification of Diseases and Related Health Problems, Tenth Revision* (*ICD-10*) system; personnel only consider physician-recorded diagnoses in a patient’s medical record when assigning codes and do not review or interpret symptoms or test results. We have used these databases before to study prescription drug safety.^17,18,19,20,21^ Except for prescriber data (8% missing, defined as a separate category) and neighborhood income quintile (0.2% missing, recorded as the middle quintile), the databases were complete for all variables in this study. The only reason for loss to follow-up was emigration from the province, which is less than 0.5% per year on average.^22^ The codes used to ascertain comorbidities and outcomes are detailed in eTable 5 in Supplement 1. We assessed comorbidities in the 5-year period before cohort entry and health care use in the 1-year period before cohort entry. We used a 120-day look-back period to ascertain use of other prescription drugs because the Ontario Drug Benefits program allows a maximum prescription duration of 100 days.

### Patients

The primary cohort included adults aged 66 years and older who had an estimated glomerular filtration rate (eGFR) of less than 60 mL/min/1.73 m^2^ (excluding patients receiving dialysis and kidney transplant recipients) and who were newly dispensed oral methotrexate or hydroxychloroquine from an outpatient pharmacy between January 1, 2008, and July 31, 2021. The prescription fill date was the patient’s cohort entry date (the index date); patients could only enter the cohort once. The age restriction was applied to ensure all patients in the study had at least 1 year of provincial universal prescription drug coverage before their index date. The GFR was estimated using the most recent outpatient serum creatinine measurement before the index date (using the isotope dilution mass spectroscopy–traceable enzymatic method), and eGFR was calculated using the new race-free CKD Epidemiology (CKD-EPI) equation: 142 × min([serum creatinine concentration in μmol/L/88.4]/ĸ, 1)α × max([serum creatinine concentration in μmol/L/88.4]/ĸ, 1)-1.200 × 0.9938Age ×1.012 [if female] ĸ = 0.7 if female and 0.9 if male; α = -0.241 if female and −0.302 if male; min = the minimum of serum creatinine concentration/ĸ or 1; max = the maximum of serum creatinine concentration/ĸ or 1.^23^ Ontarians with African ancestry represented less than 5% of the population in 2016.^24^ In Ontario, many older adults have at least 1 outpatient serum creatinine measurement in routine care each year, where a single value represents a stable, chronic value.^25^ Patients with no serum creatinine measurements in the year before the index date were excluded.

To ensure that patients were new users, we excluded those with any evidence of methotrexate or hydroxychloroquine use in the 180-day period before the index date and those with any evidence of other DMARD use (conventional or biologics) in the 30 days before the index date. We also excluded patients who were discharged from the hospital or emergency department within 2 days before the index date. (In Ontario, DMARD outpatient prescriptions are given on the same or next day after a hospital stay.) To ensure generalizability to usual prescribing, we excluded patients who started nonstandard doses (ie, methotrexate <5 mg/wk or >35 mg/wk; hydroxychloroquine <200 mg/d or >400 mg/d). A diagram summarizing the cohort creation steps is provided in eFigure 1 in Supplement 1.

The secondary cohorts included (1) patients who started methotrexate at 15 to 35 mg/wk and those who started hydroxychloroquine and (2) patients who started methotrexate at 5 to <15 mg/wk and those who started hydroxychloroquine. The 2 cohorts were not mutually exclusive (ie, hydroxychloroquine users were included in both cohorts). To better characterize the risk modification by baseline category of eGFR (≥60, 45-59, and <45 mL/min/1.73 m^2^), we added to the primary cohort of patients with CKD an additional cohort of patients with normal kidney function (ie, patients with an eGFR ≥60 mL/min/1.73 m^2^).

### Exposure

The primary exposure was low-dose oral methotrexate (5-35 mg/wk). An active comparator, oral hydroxychloroquine (200-400 mg/d), was chosen as the primary comparator to reduce the influence of indication bias. As a DMARD, hydroxychloroquine has some of the same indications as methotrexate, including rheumatoid arthritis, systemic lupus erythematosus, and dermatomyositis.^26^ We considered other DMARDs for the comparator (eg, leflunomide and sulfasalazine)^2,27,28,29^; however, these drugs have been associated with the primary outcome (eg, hepatotoxic effects for leflunomide and myelosuppression for sulfasalazine).^28,29^ For the secondary objective, patients starting methotrexate at 15 to 35 mg/wk and 5 to less than 15 mg/week were compared separately with hydroxychloroquine users.

### Outcomes

All primary and secondary outcomes were prespecified. The primary outcome was the 90-day risk of a hospital visit (ie, an emergency department visit or a hospital admission) with myelosuppression (defined as a diagnosis of aplastic anemia, neutropenia, thrombocytopenia, or pancytopenia), sepsis, pneumotoxic effects, or hepatotoxic effects. These adverse events have been reported in several randomized clinical trials of low-dose methotrexate^10,11,12,13^ and have been linked to myelosuppression, sepsis, pneumotoxic effects, and hepatotoxic effects in patients with CKD (eTable 4 in Supplement 1). We combined these outcomes into a composite to increase statistical power because, individually, they occur infrequently in Ontario. The 90-day follow-up was defined based on a review of the literature (eTable 4 in Supplement 1); in these studies, the median (IQR) time to toxic effects after methotrexate initiation was 26 (IQR 8-60) days. Acute kidney injury was not included in the primary outcome because a recent randomized clinical trial did not support a causal association with low-dose methotrexate.^30^

The secondary outcomes were the components of the primary composite outcome, all-cause hospitalization, and all-cause mortality. Diagnostic codes for all outcome variables and information on their validation and interpretation are provided in eTable 6 in Supplement 1.

### Statistical Analysis

Analyses were conducted using SAS version 9.4 (SAS Institute). Propensity score matching was used to balance comparison groups on indicators of baseline health, including all known indications for methotrexate use (including off-label indications).^31,32^ The propensity score was estimated using multivariable logistic regression with 140 covariates chosen (eTable 7 in Supplement 1) because they are known confounders or risk factors for study outcomes, including the year of cohort entry, which serve as a proxy for changes in health care practices and drug use during the study period. This selection was based on subject matter knowledge and previous literature.^31,32^ Using greedy matching, we matched each low-dose methotrexate user (1:1) to a hydroxychloroquine user based on the logit of the propensity score (within a caliper of ±0.2 SDs).^33^ In simulation studies, greedy matching with a caliper width of 0.2 SD produced less bias than optimal and nearest-neighbor matching.^34,35^

Between-group differences in baseline characteristics were compared using standardized differences in both the unmatched and matched samples (differences >10% were considered meaningful).^36^ Risk ratios and 95% CIs were obtained using modified Poisson regression,^37^ and a sandwich variance estimator was used to account for the correlation within matched pairs. Risk differences and 95% CIs were obtained using a binomial regression model with an identity link function which also accounted for the correlation within matched pairs. We interpreted 2-tailed *P* < .05 as statistically significant. Because of the potential for type I error due to multiple comparisons, findings of the secondary, subgroup, and sensitivity analyses should be interpreted as exploratory. To comply with ICES privacy regulations to minimize the risk of identification, values of cells with 5 or fewer patients were reported as less than 6.

A prespecified subgroup analysis for the primary composite outcome by baseline eGFR (grouped into 3 categories: ≥60, 45-59, and <45 mL/min/1.73 m^2^) was conducted by including an interaction term in the model. To ensure that baseline health indicators were balanced between comparison groups, we recalculated the propensity score within each eGFR category.^38^ We then matched each methotrexate user 1:1 to a hydroxychloroquine user based on the logit of the propensity score (within a caliper of ±0.2 SDs) as in the primary analysis.^33^

To address the secondary objective, each group of low-dose methotrexate users (15 to 35 mg/wk and 5 to <15 mg/wk) was compared separately to hydroxychloroquine users on the risk of the primary outcome. Propensity score matching was performed separately for these comparisons.

We conducted the following post hoc analyses to assess the robustness of the main results and examine the potential for bias. We performed a survival analysis with a 90-day follow-up censoring on death (the proportional hazards assumption was met: methotrexate × follow-up time interaction term, *P* = .99). To assess the potential for indication bias, we reran the primary analysis using 2 different weighting methods^39^ and we compared those who started methotrexate at 15 to 35 mg/wk with those who started methotrexate at 5 to less than 15mg/wk. To examine the potential for unmeasured confounding, we conducted (1) an E-value analysis to assess the extent of unmeasured confounding that would be required to negate the observed results^40^ and (2) an analysis using a negative-control outcome,^41^ defined as receipt of a hearing test during an outpatient or hospital visit. To account for surveillance bias, we compared the proportion of patients in each group who received a test for complete blood count or liver function or who received a chest radiograph in follow-up. Respectively, these tests may be used to assess myelosuppression, hepatoxic effects, and pneumotoxic effects (the components of the primary outcome). To control for potential surveillance bias, we repeated the primary analysis restricted to patients who received at least 1 of these tests in follow-up. We also repeated the primary analysis using (1) a more restrictive outcome definition (ie, only counting hospital admissions with the primary outcome, but not emergency department visits) and (2) a 30-day follow-up period.

## Results

### Patients

The primary cohort included 6909 older adults with an eGFR of less than 60 mL/min/1.73 m^2^ who were newly dispensed low-dose methotrexate (n = 2900) or hydroxychloroquine (n = 4009) at an outpatient pharmacy. The flow diagram for the cohort build is shown in the Figure.

**Figure.**
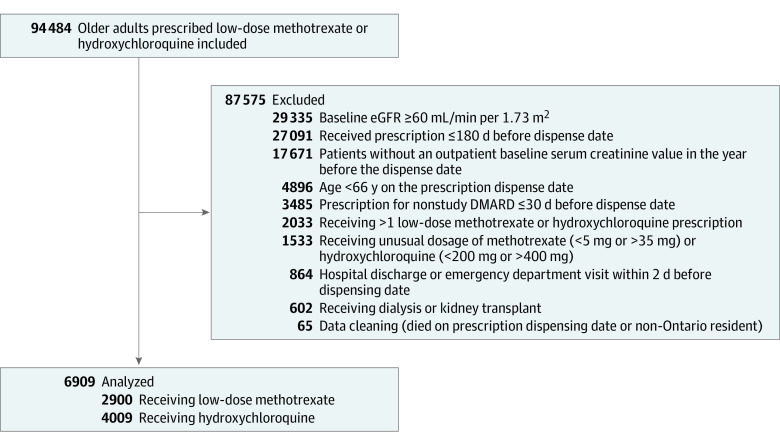
Flow Diagram of Cohort Build DMARD indicates disease-modifying antirheumatic drug; eGFR, estimated glomerular filtration rate.

Before matching, 130 of 141 variables (92%) had standardized differences of less than 10% (variables with differences >10% were sex; Local Health Integration Network; prescriber type; history of psoriasis, dermatomyositis, or systemic lupus erythematosus; concurrent use of glucocorticoids; and receipt of a chest radiograph, mammography, serum thyroid-stimulating hormone test, or serum parathyroid hormone test in the prior 12 months) (eTable 8 in Supplement 1). We matched 2309 methotrexate users (80%) with 2309 hydroxychloroquine users.

After matching, standardized differences for all 141 variables were less than 10% (eTable 8 in Supplement 1). In the matched cohort, the median (IQR) age was 76 (71-82) years, and 3192 (69%) were female; recorded diagnoses included rheumatoid arthritis (2579 [56%]), atopic dermatitis or eczema (1297 [28%]), systemic lupus erythematosus (474 [10%]), dermatomyositis (366 [8%]), systemic sclerosis (342 [7%]), and psoriasis (259 [5%]) (Table 1). Patients received prescriptions for low-dose methotrexate or hydroxychloroquine primarily from rheumatologists (2744 [59%]), primary care physicians (685 [15%]), internists (365 [8%]), and dermatologists (206 [5%]). Most patients (3168 [69%]) had an eGFR of 45 to 59 mL/min/1.73 m^2^, followed by an eGFR of 30 to 45 mL/min/1.73 m^2^ (1208 [26%]), and the remaining 242 (5%) had an eGFR less than 30 mL/min/1.73 m^2^. The median (IQR) prescribed doses of methotrexate and hydroxychloroquine were 15 (10.0-17.5) mg/wk and 400 (200-400) mg/d, respectively (eTable 9 in Supplement 1). The median (IQR) duration of continuous dispensing was 145 (59-424) days for methotrexate and 124 (30-438) days for hydroxychloroquine (eTable 9 in Supplement 1).

**Table 1.** Baseline Characteristics of Older Adults With CKD Newly Prescribed Low-Dose Methotrexate vs Hydroxychloroquine in Ontario, Canada, 2008-2021^a^

Characteristics	Unmatched data (n = 6909)			Matched data (n = 4618)		
	Patients, No. (%)		Standardized difference, %^b^	Patients, No. (%)		Standardized difference, %^b^
	Low-dose methotrexate (n = 2900)	Hydroxychloroquine (n = 4009)		Low-dose methotrexate (n = 2309)	Hydroxychloroquine (n = 2309)	
Demographics						
Sex						
Female	1918 (66.1)	2958 (73.8)	17	1611 (69.8)	1581 (68.5)	3
Male	982 (33.9)	1051 (26.2)	17	698 (30.2)	728 (31.5)	3
Age, mean (SD), y	77.2 (6.9)	76.9 (6.8)	5	77.1 (6.9)	77.1 (6.9)	0
Residence						
Urban	2517 (86.8)	3513 (87.6)	2	2017 (87.4)	2011 (87.1)	1
Rural	383 (13.2)	496 (12.4)	2	292 (12.6)	298 (12.9)	1
Long-term care	42 (1.4)	37 (0.9)	5	28 (1.2)	30 (1.3)	1
Income quintile^c^						
1 (Lowest)	566 (19.5)	781 (19.5)	0	431 (18.7)	433 (18.8)	0
2	630 (21.7)	874 (21.8)	0	507 (22.0)	484 (21.0)	2
3 (Middle)	619 (21.3)	835 (20.8)	1	489 (21.2)	502 (21.7)	1
4	546 (18.8)	780 (19.5)	2	441 (19.1)	465 (20.1)	3
5 (Highest)	539 (18.6)	739 (18.4)	1	441 (19.1)	425 (18.4)	2
Kidney function						
eGFR, mL/min/1.73m^2^^d^						
Mean (SD)	48.5 (9.1)	47.5 (9.9)	10	48.2 (9.3)	48.1 (9.6)	1
<30	136 (4.7)	266 (6.6)	8	116 (5.0)	126 (5.5)	2
30-44	745 (25.7)	1100 (27.4)	4	612 (26.5)	596 (25.8)	2
45-59	2018 (69.6)	2643 (65.9)	8	1581 (68.5)	1587 (68.7)	0
Prescriber						
Rheumatologist	1540 (53.1)	2471 (61.6)	17	1354 (58.6)	1390 (60.2)	3
General practitioner	428 (14.8)	619 (15.4)	2	347 (15.0)	338 (14.6)	1
Dermatologist	321 (11.1)	116 (2.9)	33	104 (4.5)	102 (4.4)	0
Internist	210 (7.2)	296 (7.4)	1	184 (8.0)	181 (7.8)	1
Other	158 (5.4)	180 (4.5)	4	115 (5.0)	110 (4.8)	1
Missing	243 (8.4)	327 (8.2)	1	205 (8.9)	188 (8.1)	3
Comorbidities^e^						
Rheumatoid arthritis	1500 (51.7)	1922 (47.9)	8	1271 (55.0)	1308 (56.6)	3
Atopic dermatitis or eczema	918 (31.7)	1154 (28.8)	6	642 (27.8)	655 (28.4)	1
Psoriasis	448 (15.4)	130 (3.2)	43	132 (5.7)	127 (5.5)	1
Systemic lupus erythematosus	264 (9.1)	664 (16.6)	23	236 (10.2)	238 (10.3)	0
Systemic sclerosis	221 (7.6)	305 (7.6)	0	172 (7.4)	170 (7.4)	0
Dermatomyositis	191 (6.6)	546 (13.6)	23	182 (7.9)	184 (8.0)	1
Modified Charlson comorbidity index, mean (SD)^f^	2.5 (1.2)	2.5 (1.1)	7	2.5 (1.2)	2.5 (1.2)	1
Health care visits and tests^g^						
Primary care visits, mean (SD)	10.5 (9.3)	10.3 (9.8)	2	10.5 (9.5)	10.4 (10.4)	1
Emergency department visits, mean (SD)	0.8 (1.4)	0.7 (1.4)	5	0.7 (1.4)	0.8 (1.4)	1
Medication use^h^						
Statins	1439 (49.6)	2128 (53.1)	7	1142 (49.5)	1157 (50.1)	1
Benzodiazepine	385 (13.3)	569 (14.2)	3	312 (13.5)	311 (13.5)	0

Abbreviations: CKD, chronic kidney disease; eGFR, estimated glomerular filtration rate.

^a^ Unless otherwise specified, baseline characteristics were assessed on the cohort entry date.

^b^ A value greater than 10 is interpreted as a meaningful difference.

^c^ Income was categorized into fifths of average neighborhood income on the index date.

^d^ The most recent eGFR measurement in the 7-to-365–day period before the index date.

^e^ Baseline comorbidities were assessed in the 5-year period before the index date.

^f^ Presence of kidney disease is a variable in the Charlson comorbidity index, so that all individuals have a minimum score of 2.

^g^ Total number of health care visits and tests in the 12-month period before the index date.

^h^ Medication use was examined in the 120-day period before the index date.

### Study Outcomes

The primary outcome, the 90-day risk of a hospital visit with myelosuppression, sepsis, pneumotoxic effects, hepatotoxic effects occurred in 82 of 2309 patients (3.55%) who started methotrexate and in 40 of 2309 patients (1.73%) who started hydroxychloroquine (risk ratio, 2.05 [95% CI, 1.42-2.96]; risk difference, 1.82% [95% CI, 0.91%-2.73%]). In patients who experienced the outcome, the median (IQR) time from starting the prescription to the outcome was 49 (28-68) days in the methotrexate group and 43 (22-70) days in the hydroxychloroquine group. Additional descriptive characteristics of these patients are shown in eTable 10 in Supplement 1. The number of events for each component of the composite outcome (ie, myelosuppression, sepsis, pneumotoxic effects, hepatotoxic effects) are shown in eTable 11 in Supplement 1. Starting low-dose methotrexate vs hydroxychloroquine was associated with a higher risk of a hospital visit with myelosuppression (risk ratio, 4.40 [95% CI, 1.73-11.20]; risk difference, 0.74% [95% CI, 0.31%-1.16%]), pneumotoxic effects (risk ratio, 2.28 [95% CI, 1.43-3.63]; risk difference, 1.39% [95% CI, 0.63%-2.14%]), and all-cause hospitalization (risk ratio, 1.39 [95% CI, 1.13-1.69]; risk difference, 2.51% [95% CI, 0.98%-4.04%]), but not a hospital visit with sepsis or all-cause mortality (Table 2).

**Table 2.** Risk of a Hospital Visit With Myelosuppression, Sepsis, Pneumotoxic Effects, or Hepatotoxic Effects in Older Adults With CKD Within 90 Days of Starting a New Prescription for Low-Dose Methotrexate vs Hydroxychloroquine^a^

Outcome	Events, No. (%)				Risk difference, % (95% CI)	NNH (95% CI)	Risk ratio (95% CI)
	Unmatched		Matched^b^				
	Low-dose methotrexate (n = 2900)	Hydroxy chloroquine (n = 4009)	Low-dose methotrexate (n = 2309)	Hydroxy chloroquine (n = 2309)			
Primary outcome^c^	97 (3.34)	66 (1.65)	82 (3.55)	40 (1.73)	1.82 (0.91 to 2.73)	55 (37 to 110)	2.05 (1.42 to 2.96)
Secondary outcomes							
Hospital visit with myelosuppression^d^	30 (1.03)	9 (0.22)	NR	NR	0.74 (0.31 to 1.16)	135 (86 to 323)	4.40 (1.73 to 11.20)
Hospital visit with sepsis	21 (0.72)	18 (0.45)	20 (0.87)	12 (0.52)	0.35 (−0.13 to 0.83)	NA	1.67 (0.81 to3.41)
Hospital visit with pneumotoxic effects	65 (2.24)	41 (1.02)	57 (2.47)	25 (1.08)	1.39 (0.63 to 2.14)	72 (47 to 159)	2.28 (1.43 to 3.63)
All-cause hospitalization	269 (9.28)	260 (6.49)	208 (9.01)	150 (6.50)	2.51 (0.98 to 4.04)	40 (25 to 102)	1.39 (1.13 to 1.69)
All-cause mortality	49 (1.69)	41 (1.02)	38 (1.65)	27 (1.17)	0.48 (−0.21 to 1.16)	NA	1.41 (0.86 to 2.31)

Abbreviations: NA, not applicable; NNH, number needed to harm; NR, not reported.

^a^ Reference group was hydroxychloroquine.

^b^ The propensity score was estimated using multivariable logistic regression with 140 covariates chosen a priori (eTable 7 in Supplement 1). Greedy matching was used to match each low-dose methotrexate user (1:1) to a hydroxychloroquine user based on the logit of the propensity score (within a caliper of ±0.2 SDs).^33^ Risk ratios and 95% CIs were obtained using modified Poisson regression^34^ and risk differences and 95% CIs were obtained using a binomial regression model with an identity link function.

^c^ The 90-day risk of a hospital visit with myelosuppression, sepsis, pneumotoxic effects, or hepatotoxic effects. The components of the primary outcome are not mutually exclusive, as such, some individuals experience more than 1 of the components of the primary outcome. To comply with ICES privacy regulations to minimize the risk of identification, specific values of cells with 5 or fewer patients were suppressed (reported as <6). As a result, the number of adverse events for hepatotoxic effects is not presented in the table.

^d^ Defined as a diagnosis of aplastic anemia, neutropenia, thromobocytopenia, or pancytopenia. The components of the primary outcome are not mutually exclusive, as such, some individuals experience more than 1 of the components of the primary outcome. To comply with ICES privacy regulations to minimize the risk of identification, specific values of cells with 5 or fewer patients were suppressed (reported as NR). As a result, the number of adverse events for this outcome is not presented in the matched cohort in the table.

### Prespecified Subgroup Analysis

The results of the subgroup analyses by baseline eGFR categories are shown in Table 3. The risk ratios and risk differences for the primary outcome increased significantly and progressively as eGFR declined (eg, eGFR <45 mL/min/1.73 m^2^: RR, 2.79 [95% CI, 1.51-5.13]; *P* = .003 for additive interaction; *P* = .008 for multiplicative interaction). The characteristics of low-dose methotrexate and hydroxychloroquine users within eGFR categories are presented in eTables 12 to 14 in Supplement 1.

**Table 3.** Subgroup Analysis for the Risk of the Primary Composite Outcome by eGFR Category^a^^,^^b^

eGFR group,^c^ mL/min per 1.73 m^2^	Study drug	Matched		Risk difference, % (95% CI)	*P* value^d^	NNH (95% CI)	Risk ratio (95% CI)	*P* value^e^
		Patients, No.	Events, No. (%)					
≥60	Low-dose methotrexate	10 277	112 (1.09)	0.03 (−0.25 to 0.31)	.003	NA	1.03 (0.79 to 1.33)	.008
	Hydroxychloroquine	10 277	109 (1.06)					
45-59	Low-dose methotrexate	1536	35 (2.28)	0.46 (−0.54 to 1.45)		NA	1.25 (0.77 to 2.04)	
	Hydroxychloroquine	1536	28 (1.82)					
<45	Low-dose methotrexate	706	39 (5.52)	3.54 (1.54 to 5.55)		28 (18 to 65)	2.79 (1.51 to 5.13)	
	Hydroxychloroquine	706	14 (1.98)					

Abbreviations: eGFR, estimated glomerular filtration rate; NA, not applicable; NNH, number needed to harm.

^a^ The propensity score was estimated using multivariable logistic regression with 140 covariates chosen a priori (eTable 7 in Supplement 1). Greedy matching was used to match each low-dose methotrexate user (1:1) to a hydroxychloroquine user based on the logit of the propensity score (within a caliper of ±0.2 SDs).^33^ Risk ratios and 95% CIs were obtained using modified Poisson regression^34^ and risk differences and 95% CIs were obtained using a binomial regression model with an identity link function.

^b^ The 90-day risk of a hospital visit with myelosuppression, sepsis, pneumotoxic effects, or hepatotoxic effects.

^c^ To ensure that baseline health indicators were balanced between comparison groups, the propensity score was recalculated within each eGFR category.^35^ Each methotrexate drug user was then matched 1:1 to a hydroxychloroquine user based on the logit of the propensity score (within a caliper of ±0.2 SDs) as in the primary analysis.^33^

^d^ *P* value for additive interaction.

^e^ *P* value for multiplicative interaction.

### Risk of the Primary Outcome in New Users of 2 Different Methotrexate Doses vs Hydroxychloroquine

Starting low-dose methotrexate at 15 to 35 mg/week vs hydroxychloroquine was associated with a higher 90-day risk of the primary composite outcome (52 of 1357 [3.83%] vs 16 of 1357 [1.18%]; risk ratio, 3.25 [95% CI, 1.87-5.64]; risk difference, 2.65% [95% CI, 1.49%-3.82%]) (eTable 15 in Supplement 1). Characteristics of patients starting methotrexate at 15 to 35 mg/wk vs those starting hydroxychloroquine are shown in eTable 16 in Supplement 1.

Starting low-dose methotrexate at 5 to less than 15 mg/wk vs hydroxychloroquine was not associated with a higher 90-day risk of the primary composite outcome (34 of 1212 [2.81%] vs 22 of 1212 [1.82%]; risk ratio, 1.55 [95% CI, 0.90 to 2.64]; risk difference, 0.99% [95% CI, −0.22% to 2.20%]) (eTable 17 in Supplement 1). Characteristics of patients starting low-dose methotrexate at 5 to less than 15 mg/wk vs those starting hydroxychloroquine are shown in eTable 18 in Supplement 1.

### Post Hoc Analyses

Results were consistent when the data were analyzed using a Cox proportional hazards regression (eTable 19 in Supplement 1) and when we used 2 different weighting techniques (eTable 20 and 21 in Supplement 1). Results were also consistent when we compared patients starting methotrexate at 15 to 35 mg/wk vs 5 to less than 15 mg/week (risk ratio, 2.15 [95% CI, 1.30-3.56]; risk difference, 2.13% [95% CI, 0.85%-3.40%]) (eTable 22 ad eTable 23 in Supplement 1). The E-values for the risk ratio and lower confidence bound for the primary outcome were 3.52 and 2.19, respectively (eFigure 2 in Supplement 1). Study results were also supported by the analysis of a negative-control outcome (eTable 24 in Supplement 1). The proportion of patients who received a test for a complete blood count, liver function, or a chest radiograph during the follow-up was higher in patients starting low-dose methotrexate vs hydroxychloroquine (eTable 25 in Supplement 1); however, results remained consistent when the analysis was restricted to patients who received at least 1 of these tests during follow-up (eTable 26 in Supplement 1), when we used a more restrictive outcome definition (eTable 27 in Supplement 1), and when we used a 30-day follow-up period (eTable 28 in Supplement 1).

## Discussion

In this study of older adults with CKD, those who started a prescription for low-dose methotrexate (median dose 15 mg/wk) had a significantly greater 90-day risk of hospital visit with myelosuppression, sepsis, pneumotoxic effects, or hepatotoxic effects compared with those who started hydroxychloroquine. The risks were amplified at lower levels of eGFR. For every 28 patients with moderate-to-severe CKD (ie, an eGFR <45mL/min/1.73 m^2^) who were prescribed low-dose methotrexate vs hydroxychloroquine, 1 patient was hospitalized with a serious adverse event. Pneumotoxic effects were the most frequent adverse event. Results were consistent in multiple sensitivity analyses. In a secondary comparison, the risk of toxic effects was greater in patients who started methotrexate at 15 to 35 mg/wk vs hydroxychloroquine.

The study did not assess whether low-dose methotrexate had more benefits than risks. Whether the potential benefits outweigh the risks will need to be evaluated individually.

This population-based study of 4618 older adults confirms and extends the findings of 50 case reports and 2 retrospective cohort studies that reported an association between methotrexate use and toxic effects in patients with CKD (eTable 4 in Supplement 1). This study has several strengths, including its population-based design, the inclusion of a representative sample of older CKD patients, and the use of propensity score matching to ensure similar baseline characteristics. Multiple sensitivity analyses confirmed the main findings.

### Limitations

This study has several limitations. First, the observational study design precludes reaching causal conclusions. Second, patients’ adherence to study medications is unknown. Third, the study focused on older patients with CKD, so results may not apply to younger populations or patients undergoing dialysis. Fourth, the risk-benefit ratio of low-dose methotrexate was not assessed, and the study likely underestimated adverse events not resulting in hospital visits. Residual confounding and surveillance bias are possible. Additionally, the small number of patients with hepatotoxic effects precluded further analysis of this outcome.

## Conclusions

Among older patients with CKD, the 90-day risk of serious adverse events was higher among those starting low-dose methotrexate compared with those starting hydroxychloroquine. Therefore, patients with CKD starting low-dose methotrexate should have active surveillance, including blood tests and chest radiographs performed regularly to monitor for signs of myelosuppression, infection, hepatotoxic effects, and pneumotoxic effects. The risks of using low-dose methotrexate in patients with CKD should be balanced against its benefits. If our findings are replicated in other jurisdictions, we recommend improving and updating product monographs and prescribing guidelines. The US Food and Drug Administration and Health Canada should also consider issuing warning labels to inform prescribers of the study’s findings.

## Notes

Supplement 1. **eTable 1.** Recommended dose of oral methotrexate based on a patient’s kidney function: guidelines from UpToDate and the product monograph **eTable 2.** Summary of a pharmacokinetic study of low-dose methotrexate (LD-MTX) in rheumatoid arthritis (RA) patients with and without kidney impairment **eTable 3.** Literature search **eTable 4.** Summary of studies of methotrexate-associated adverse events in patients with chronic kidney disease **eTable 5.** Coding definitions for demographic and comorbid conditions **eTable 6.** Operating characteristics of hospital diagnosis codes used to define the primary and secondary outcomes **eTable 7.** Variables included in the propensity score model **eTable 8.** Baseline characteristics of older adults with chronic kidney disease newly prescribed low-dose methotrexate (LD MTX) vs those newly prescribed hydroxychloroquine (HCQ) in Ontario, Canada (2008–2021) **eTable 9.** Dose and duration of continuous study DMARD dispensing in older adults with chronic kidney disease newly prescribed LD-MTX vs. HCQ in Ontario, Canada (2008-2021) **eTable 10.** Characteristics of 41 patients with a hospital admission with serious adverse events after starting a study DMARD in the matched cohort **eTable 11.** Frequency of each component in the matched cohort **eTable 12.** Baseline characteristics of older adults with an eGFR>60 mL/min/1.73m2 newly prescribed low-dose methotrexate (LD MTX) vs those newly prescribed hydroxychloroquine (HCQ) in Ontario, Canada (2008–2021) **eTable 13.** Baseline characteristics of older adults with an eGFR between 45 and <60 mL/min/1.73m2 newly prescribed low-dose methotrexate (LD MTX) vs those newly prescribed hydroxychloroquine (HCQ) in Ontario, Canada (2008–2021) **eTable 14.** Baseline characteristics of older adults with an eGFR <45 mL/min/1.73m2 newly prescribed low-dose methotrexate (LD MTX) vs those newly prescribed hydroxychloroquine (HCQ) in Ontario, Canada (2008–2021) **eTable 15.** Risk of a hospital visit with myelosuppression, sepsis, pneumotoxicity, and hepatotoxicity in older adults with chronic kidney disease within 90 days of starting a new prescription for low-dose methotrexate at 15 to 35 mg/week vs a new prescription for hydroxychloroquine **eTable 16.** Baseline characteristics of older adults with chronic kidney disease newly prescribed low-dose methotrexate (LD MTX) at 15 to 35 mg/week vs those newly prescribed hydroxychloroquine (HCQ) in Ontario, Canada (2008–2021) **eTable 17.** Risk of a hospital visit with myelosuppression, sepsis, pneumotoxicity, and hepatotoxicity in older adults with chronic kidney disease within 90 days of starting a new prescription for low-dose methotrexate at 5 to <15 mg/week vs a new prescription for hydroxychloroquine **eTable 18.** Baseline characteristics of older adults with chronic kidney disease newly prescribed low-dose methotrexate (LD MTX) at 5 to <15 mg/week vs those newly prescribed hydroxychloroquine (HCQ) in Ontario, Canada (2008–2021) **eTable 19.** Survival analysis in older adults with chronic kidney disease who started a new prescription for low-dose methotrexate- vs a new prescription for hydroxychloroquine: risk of a hospital visit with myelosuppression, sepsis, pneumotoxicity, and hepatotoxicity **eTable 20.** Risk of a hospital visit with myelosuppression, sepsis, pneumotoxicity, and hepatotoxicity in older adults with chronic kidney disease within 90 days of starting a new prescription for low-dose methotrexate vs. a new prescription for hydroxychloroquine using Inverse probability of treatment weighting analysis **eTable 21.** Risk of a hospital visit with myelosuppression, sepsis, pneumotoxicity, and hepatotoxicity in older adults with chronic kidney disease within 90 days of starting a new prescription for low-dose methotrexate vs a new prescription for hydroxychloroquine using Fine stratification weighting **eTable 22.** Risk of a hospital visit with myelosuppression, sepsis, pneumotoxicity, and hepatotoxicity in older adults with chronic kidney disease within 90 days of starting a new prescription for low-dose methotrexate at 15 to 35 mg/week vs 5 to <15 mg/week **eTable 23.** Baseline characteristics of older adults with chronic kidney disease newly prescribed low-dose methotrexate (LD MTX) at 15 to 35mg/week vs 5 to<15 mg/week in Ontario, Canada (2008–2021) **eTable 24.** Risk of an outpatient or hospital visit with hearing disorders in older adults with chronic kidney disease within 90 days of starting a new prescription for low-dose methotrexate vs a new prescription for hydroxychloroquine **eTable 25.** Proportion of patients who received a test for a complete blood count, liver function, or a chest radiograph within 90 days of starting a new prescription for low-dose methotrexate vs. a new prescription for hydroxychloroquine **eTable 26.** Risk of a hospital visit with myelosuppression, sepsis, pneumotoxicity, and hepatotoxicity in older adults with chronic kidney disease who received at least one test within 90 days of starting a new prescription for low-dose methotrexate vs a new prescription for hydroxychloroquine **eTable 27.** Risk of a hospital admission with myelosuppression, sepsis, pneumotoxicity, and hepatotoxicity in older adults with chronic kidney disease within 90 days of starting a new prescription for low-dose methotrexate vs a new prescription for hydroxychloroquine **eTable 28.** Risk of a hospital visit with myelosuppression, sepsis, pneumotoxicity, and hepatotoxicity in older adults with chronic kidney disease within 30 days of starting a new prescription for low-dose methotrexate vs. a new prescription for hydroxychloroquine **eFigure 1.** Study design diagram comparing use of methotrexate vs hydroxychloroquine and the risk of serious adverse events in patients with chronic kidney disease **eFigure 2.** E-value analysis to assess the extent of unmeasured confounding that would be required to negate the observed results
eReferences.

Supplement 2.
Data Sharing Statement
