# Supplementary material for: Low-Dose Methotrexate and Serious Adverse Events Among Older Adults With Chronic Kidney Disease
Source: JAMA Netw Open. 2023 Nov 27;6(11):e2345132. doi: 10.1001/jamanetworkopen.2023.45132 (PMC10682837; doi:10.1001/jamanetworkopen.2023.45132)
Supplement: Supplement 1. — eTable 1. Recommended dose of oral methotrexate based on a patient’s kidney function: guidelines from UpToDate and the product monograph eTable 2. Summary of a pharmacokinetic study of low-dose methotrexate (LD-MTX) in rheumatoid arthritis (RA) patients with and without kidney impairment eTable 3. Literature search eTable 4. Summary of studies of methotrexate-associated adverse events in patients with chronic kidney disease eTable 5. Coding definitions for demographic and comorbid conditions eTable 6. Operating characteristics of hospital diagnosis codes used to define the primary and secondary outcomes eTable 7. Variables included in the propensity score model eTable 8. Baseline characteristics of older adults with chronic kidney disease newly prescribed low-dose methotrexate (LD MTX) vs those newly prescribed hydroxychloroquine (HCQ) in Ontario, Canada (2008–2021) eTable 9. Dose and duration of continuous study DMARD dispensing in older adults with chronic kidney disease newly prescribed LD-MTX vs. HCQ in Ontario, Canada (2008-2021) eTable 10. Characteristics of 41 patients with a hospital admission with serious adverse events after starting a study DMARD in the matched cohort eTable 11. Frequency of each component in the matched cohort eTable 12. Baseline characteristics of older adults with an eGFR>60 mL/min/1.73m2 newly prescribed low-dose methotrexate (LD MTX) vs those newly prescribed hydroxychloroquine (HCQ) in Ontario, Canada (2008–2021) eTable 13. Baseline characteristics of older adults with an eGFR between 45 and <60 mL/min/1.73m2 newly prescribed low-dose methotrexate (LD MTX) vs those newly prescribed hydroxychloroquine (HCQ) in Ontario, Canada (2008–2021) eTable 14. Baseline characteristics of older adults with an eGFR <45 mL/min/1.73m2 newly prescribed low-dose methotrexate (LD MTX) vs those newly prescribed hydroxychloroquine (HCQ) in Ontario, Canada (2008–2021) eTable 15. Risk of a hospital visit with myelosuppression, sepsis, pneumotoxicity, and h [file jamanetwopen-e2345132-s001.pdf]

Supplemental Online Content

Muanda FT, Blake PG, Weir MA, et al. Low-dose methotrexate and serious adverse events among older adults with chronic kidney disease. *JAMA Netw Open.* 2023;6(11):e2345132. doi:10.1001/jamanetworkopen.2023.45132

eTable 1 . Recommended dose of oral methotrexate based on a patient's kidney function: guidelines from UpToDate and the product monograph

eTable 2. Summary of a pharmacokinetic study of low-dose methotrexate (LD-MTX) in rheumatoid arthritis (RA) patients with and without kidney impairment

eTable 3. Literature search

eTable 4. Summary of studies of methotrexate-associated adverse events in patients with chronic kidney disease

eTable 5. Coding definitions for demographic and comorbid conditions

eTable 6. Operating characteristics of hospital diagnosis codes used to define the primary and secondary outcomes

eTable 7. Variables included in the propensity score model

eTable 8. Baseline characteristics of older adults with chronic kidney disease newly prescribed low-dose methotrexate (LD MTX) vs those newly prescribed hydroxychloroquine (HCQ) in Ontario, Canada (2008–2021)

eTable 9. Dose and duration of continuous study DMARD dispensing in older adults with chronic kidney disease newly prescribed LD-MTX vs. HCQ in Ontario, Canada (2008-2021)

eTable 10. Characteristics of 41 patients with a hospital admission with serious adverse events after starting a study DMARD in the matched cohort

eTable 11. Frequency of each component in the matched cohort

eTable 12. Baseline characteristics of older adults with an eGFR>60 mL/min/1.73m2 newly prescribed low-dose methotrexate (LD MTX) vs those newly prescribed hydroxychloroquine (HCQ) in Ontario, Canada (2008–2021)

eTable 13. Baseline characteristics of older adults with an eGFR between 45 and <60 mL/min/1.73m2 newly prescribed low-dose methotrexate (LD MTX) vs those newly prescribed hydroxychloroquine (HCQ) in Ontario, Canada (2008–2021)

eTable 14. Baseline characteristics of older adults with an eGFR <45 mL/min/1.73m2 newly prescribed low-dose methotrexate (LD MTX) vs those newly prescribed hydroxychloroquine (HCQ) in Ontario, Canada (2008–2021)

eTable 15. Risk of a hospital visit with myelosuppression, sepsis, pneumotoxicity, and hepatotoxicity in older adults with chronic kidney disease within 90 days of starting a new

© 2023 Muanda FT et al. *JAMA Network Open.*

prescription for low-dose methotrexate at 15 to 35 mg/week vs a new prescription for hydroxychloroquine

eTable 16. Baseline characteristics of older adults with chronic kidney disease newly prescribed low-dose methotrexate (LD MTX) at 15 to 35 mg/week vs those newly prescribed hydroxychloroquine (HCQ) in Ontario, Canada (2008–2021)

eTable 17. Risk of a hospital visit with myelosuppression, sepsis, pneumotoxicity, and hepatotoxicity in older adults with chronic kidney disease within 90 days of starting a new prescription for low-dose methotrexate at 5 to <15 mg/week vs a new prescription for hydroxychloroquine

eTable 18. Baseline characteristics of older adults with chronic kidney disease newly prescribed low-dose methotrexate (LD MTX) at 5 to <15 mg/week vs those newly prescribed hydroxychloroquine (HCQ) in Ontario, Canada (2008–2021)

eTable 19. Survival analysis in older adults with chronic kidney disease who started a new prescription for low-dose methotrexate- vs a new prescription for hydroxychloroquine: risk of a hospital visit with myelosuppression, sepsis, pneumotoxicity, and hepatotoxicity

eTable 20. Risk of a hospital visit with myelosuppression, sepsis, pneumotoxicity, and hepatotoxicity in older adults with chronic kidney disease within 90 days of starting a new prescription for low-dose methotrexate vs. a new prescription for hydroxychloroquine using Inverse probability of treatment weighting analysis

eTable 21. Risk of a hospital visit with myelosuppression, sepsis, pneumotoxicity, and hepatotoxicity in older adults with chronic kidney disease within 90 days of starting a new prescription for low-dose methotrexate vs a new prescription for hydroxychloroquine using Fine stratification weighting

eTable 22. Risk of a hospital visit with myelosuppression, sepsis, pneumotoxicity, and hepatotoxicity in older adults with chronic kidney disease within 90 days of starting a new prescription for low-dose methotrexate at 15 to 35 mg/week vs 5 to <15 mg/week

eTable 23. Baseline characteristics of older adults with chronic kidney disease newly prescribed low-dose methotrexate (LD MTX) at 15 to 35mg/week vs 5 to<15 mg/week in Ontario, Canada (2008–2021)

eTable 24. Risk of an outpatient or hospital visit with hearing disorders in older adults with chronic kidney disease within 90 days of starting a new prescription for low-dose methotrexate vs a new prescription for hydroxychloroquine

eTable 25. Proportion of patients who received a test for a complete blood count, liver function, or a chest radiograph within 90 days of starting a new prescription for low-dose methotrexate vs. a new prescription for hydroxychloroquine

eTable 26. Risk of a hospital visit with myelosuppression, sepsis, pneumotoxicity, and hepatotoxicity in older adults with chronic kidney disease who received at least one test within

© 2023 Muanda FT et al. *JAMA Network Open.*

90 days of starting a new prescription for low-dose methotrexate vs a new prescription for hydroxychloroquine

eTable 27. Risk of a hospital admission with myelosuppression, sepsis, pneumotoxicity, and hepatotoxicity in older adults with chronic kidney disease within 90 days of starting a new prescription for low-dose methotrexate vs a new prescription for hydroxychloroquine

eTable 28. Risk of a hospital visit with myelosuppression, sepsis, pneumotoxicity, and hepatotoxicity in older adults with chronic kidney disease within 30 days of starting a new prescription for low-dose methotrexate vs. a new prescription for hydroxychloroquine

eFigure 1. Study design diagram comparing use of methotrexate vs hydroxychloroquine and the risk of serious adverse events in patients with chronic kidney disease

eFigure 2. E-value analysis to assess the extent of unmeasured confounding that would be required to negate the observed results

eReferences.

This supplemental material has been provided by the authors to give readers additional information about their work.

© 2023 Muanda FT et al. *JAMA Network Open.*

eTable 1. Recommended dose of oral methotrexate based on a patient's kidney function: guidelines from UpToDate[1] and the product monograph[2]

eTable 1a. UpToDate

**UpToDate Guidelines[1]**	
Kintzel 1995[3]	CrCl >60 mL/minute: No dose adjustment necessary. The normal dose depends on the indication of methotrexate use<br>CrCl 46 to 60 mL/minute: Administer 65% of normal dose.<br>CrCl 31 to 45 mL/minute: Administer 50% of normal dose.<br>CrCl <30 mL/minute: Avoid use.
Aronoff 2007[4]	CrCl >50 mL/minute: No dose adjustment necessary.<br>CrCl 10 to 50 mL/minute: Administer 50% of dose.<br>CrCl <10 mL/minute: Avoid use.

Abbreviations: NA, not available. CrCl, Creatinine clearance

© 2023 Muanda FT et al. *JAMA Network Open.*

eTable 1b. Product monograph

**Product monograph**[2]	
Creatinine clearance (mL/min)	Standard dose to administer
>80	Full dose (100%)
80	75%
60	63%
50	56%
<50	Use alternative therapy

© 2023 Muanda FT et al. *JAMA Network Open.*

eTable 2. Summary of a pharmacokinetic study of low-dose methotrexate (LD-MTX) in rheumatoid arthritis (RA) patients with and without kidney impairment

**Author/year of publication**	**Sample Size/patient eligibility**	**Low-dose methotrexate route of administration and Dosage**	**Changes in serum concentration/elimination half-life**	**Side effects**
Bressolle et al. 1998[5]	77 RA patients aged 18 years and older were classified into one of four groups based on CrCl:<br>(1) CrCl lower than 45 ml/min;<br>(2) CrCl between 45 and 60 ml/min;<br>(3) CrCl between 61 and 80 ml/min; and<br>(4) CrCl higher than 80 ml/min	Each patient received their usual dose of LD-MTX (7.5 to 15 mg) intramuscularly	LD-MTX concentrations (total and free) were 1.3 to 1.6-times higher in group 1 than in groups 2, 3, and 4. For total and free LD-MTX, elimination half-lives were 22.7 hours in group 1, 13.5 hours in group 2, 12 hours in group 3, and 11 hours in group 4.	All adverse events were mild to moderate in intensity.<br>No difference was observed between groups. However, the number of patients included did not allow a correct toxicity evaluation in the different groups.

Abbreviations: LD-MTX, low-dose methotrexate; RA, rheumatoid arthritis. CrCl, Creatinine clearance; t1/2 elimination,

© 2023 Muanda FT et al. *JAMA Network Open.*

eTable 3. Literature search

eTable 3a. A literature search in Medline (1946 to July 19, 2022)

1	methotrexat*.ti,ab,kw.
2	methotrexat*.tw. /freq=2
3	exp Methotrexate/ad, ae, po, tu, to [Administration & Dosage, Adverse Effects, Poisoning, Therapeutic Use, Toxicity]
4	1 or 2 or 3
5	(((chronic$ or progressive or diabetic) adj (kidney or renal or nephro$ or glomerul$)) or dialy$ or h?emodia$).mp. or ckd.tw. or esrd.tw. or ((diabet$.mp. or Disease Progression/ or Recurrence/) and nephropath$.mp.) or ur?emi$.mp. or m?croalbuminuri$.mp. or albuminuri$.mp. or proteinuri$.mp. or nephrosclerosis.mp. or glomerulosclerosis.mp. or glomerular sclerosis.mp. or *Glomerular Filtration Rate/ or (secondary adj2 hyperparathyroidism).mp. or ((tubulointerstitial or interstitial or renal or kidney) adj fibrosis).tw. or hyperphosphat?emia.tw. or vascular calcification$.tw. or alport$.mp. or denys- drash.mp. or glomerulopathy.tw. or hypoalbumin?emi$.mp. or multicystic kidney$.mp. or polycystic kidney$.mp. or cystic kidney$.mp. or calciphylaxis.mp. or tenckhoff.tw. or ((kidney or renal) adj (disease$ or failur$ or function$ or insufficienc$ or disorder$ or dysfunction or replacement)).mp. or ((kidney or renal) and (ckf or crd or crf or eskd or eskf or esrf or hyperparathyroidism or end-stage or endstage or eGFR)).mp. or (((kidney or renal) adj transplant$) and (candidates or wait$ list$)).tw. or ((sclerosi$ or fibrosi$ or fibrotic).mp. and ((ureteral obstruction or nephritis or glomerulonephritis or nephrop$).mp. or (obstruct$ and (kidney$ or renal or nephropathy)).tw.))
6	exp Renal Insufficiency, Chronic/
7	5 or 6
8	exp "drug-related side effects and adverse reactions"/ or adverse.ti,ab,kf. or side effect?.ti,ab,kf. or adverse effects.fs. or exp drug overdose/ or overdos*.ti,ab,kf. or exp drug misuse/ or misus*.ti,ab,kf. or exp substance-related disorders/ or abus*.ti,ab,kf. or exp pregnancy/ or pregnan*.ti,ab,kf. or exp pregnancy complications/ or exp lactation/ or exp lactation disorders/ or exp breast feeding/ or (exp milk, human/ and exp secretion/) or exp fertility/ or exp infertility/ or exp reproduction/ or exp fetus/ or exp embryonic structures/ or terat*.ti,ab,kf. or drug efficacy.ti,ab,kf. or therapeutic efficacy.ti,ab,kf. or drug withdrawal.ti,ab,kf. or exp medication errors/ or exp death/ or death*.ti,ab,kf. or fatal*.ti,ab,kf. or exp drug interactions/ or exp carcinogens/ or carcinogen*.ti,ab,kf. or mutagen*.ti,ab,kf. or exp "off-label use"/ or exp occupational exposure/ or toxicity.fs. or toxic*.ti,ab,kf. or pharmacotox*.ti,ab,kf. or neurotox*.ti,ab,kf. or cardiotox*.ti,ab,kf. or nephrotox*.ti,ab,kf. or immunotox*.ti,ab,kf. or hepatotox*.ti,ab,kf. or cytotox*.ti,ab,kf. or immunocytotox*.ti,ab,kf. or intoxicat*.ti,ab,kf. or exp "congenital, hereditary, and neonatal diseases and abnormalities"/ or drug treatment failure.ti,ab,kf. or drug toxicity.ti,ab,kf. or exp case report/ or case report?.ti,ab,kf. or exp environmental exposure/ or treatment contraindication.ti,ab,kf. or exp contraindications, drug/ or exp "wounds and injuries"/ or suicid*.ti,ab,kf. or exp poisoning/ or poisoning.fs. or exp drug tolerance/ or exp treatment failure/ or exp drug resistance/ or exp substance-related disorders/
9	4 and 7 and 8
10	limit 9 to (english language and humans)

© 2023 Muanda FT et al. *JAMA Network Open.*

eTable 3b. Literature search in Embase (1947 to July 19, 2022)

1	methotrexat*.ti,ab,kw.
2	methotrexat*.tw. /freq=2
3	exp methotrexate/ae, ct, ad, cm, do, dt, to, im, iv, po, pv, sc, tm [Adverse Drug Reaction, Clinical Trial, Drug Administration, Drug Comparison, Drug Dose, Drug Therapy, Drug Toxicity, Intramuscular Drug Administration, Intravenous Drug Administration, Oral Drug Administration, Special Situation for Pharmacovigilance, Subcutaneous Drug Administration, Unexpected Outcome of Drug Treatment]
4	1 or 2 or 3
5	(((chronic$ or progressive or diabetic) adj (kidney or renal or nephro$ or glomerul$)) or dialy$ or h?emodia$).mp. or ckd.tw. or esrd.tw. or ((diabet$.mp. or Disease Progression/ or Recurrence/) and nephropath$.mp.) or ur?emi$.mp. or m?croalbuminuri$.mp. or albuminuri$.mp. or proteinuri$.mp. or nephrosclerosis.mp. or glomerulosclerosis.mp. or glomerular sclerosis.mp. or *Glomerular Filtration Rate/ or (secondary adj2 hyperparathyroidism).mp. or ((tubulointerstitial or interstitial or renal or kidney) adj fibrosis).tw. or hyperphosphat?emia.tw. or vascular calcification$.tw. or alport$.mp. or denys- drash.mp. or glomerulopathy.tw. or hypoalbumin?emi$.mp. or multicystic kidney$.mp. or polycystic kidney$.mp. or cystic kidney$.mp. or calciphylaxis.mp. or tenckhoff.tw. or ((kidney or renal) adj (disease$ or failur$ or function$ or insufficienc$ or disorder$ or dysfunction or replacement)).mp. or ((kidney or renal) and (ckf or crd or crf or eskd or eskf or esrf or hyperparathyroidism or end-stage or endstage or eGFR)).mp. or (((kidney or renal) adj transplant$) and (candidates or wait$ list$)).tw. or ((sclerosi$ or fibrosi$ or fibrotic).mp. and ((ureteral obstruction or nephritis or glomerulonephritis or nephrop$).mp. or (obstruct$ and (kidney$ or renal or nephropathy)).tw.))
6	exp chronic kidney failure/co, cn, dm, dt, si, th [Complication, Congenital Disorder, Disease Management, Drug Therapy, Side Effect, Therapy]
7	5 or 6
8	exp adverse drug reaction/ or adverse.ti,ab,kw. or side effect?.ti,ab,kw. or side effect.fs. or exp drug overdose/ or overdos*.ti,ab,kw. or exp drug misuse/ or misus*.ti,ab,kw. or exp drug abuse/ or exp substance abuse/ or abus*.ti,ab,kw. or exp pregnancy/ or pregnan*.ti,ab,kw. or exp pregnancy complications/ or exp lactation/ or exp breast feeding/ or (exp milk human/ and exp secretion/) or exp fertility/ or exp infertility/ or exp reproduction/ or exp fetus/ or exp embryo/ or terat*.ti,ab,kw. or exp drug efficacy/ or exp drug withdrawal/ or exp medication error/ or exp death/ or death*.ti,ab,kw. or fatal*.ti,ab,kw. or exp drug interaction/ or exp carcinogenicity/ or carcinogen*.ti,ab,kw. or mutagen*.ti,ab,kw. or exp 'off label drug use'/ or exp occupational exposure/ or exp toxicity/ or toxic*.ti,ab,kw. or pharmacotox*.ti,ab,kw. or neurotox*.ti,ab,kw. or cardiotox*.ti,ab,kw. or nephrotox*.ti,ab,kw. or immunotox*.ti,ab,kw. or hepatotox*.ti,ab,kw. or cytotox*.ti,ab,kw. or immunocytotox*.ti,ab,kw. or exp intoxication/ or exp congenital disorder/ or exp drug treatment failure/ or exp case report/ or case report?.ti,ab,kw. or exp environmental exposure/ or exp treatment contraindication/ or exp drug contraindication/ or exp injury/ or suicid*.ti,ab,kw. or exp poisoning/ or exp drug tolerance/ or exp treatment failure/ or exp drug resistance/ or exp substance-related disorders/ or drug resistance.fs.
9	4 and 7 and 8
10	limit 9 to (human and english language)

© 2023 Muanda FT et al. *JAMA Network Open.*

eTable 4 Summary of studies of methotrexate-associated adverse events in patients with chronic kidney disease

eTable 4a. Case reports and case series

Author/year	Design	Age[a]	Sex	eGFR or Cr Cl/ SCr	Indication for LD-MTX use	Dose (mg/week)[a]	Type of toxicity	Onset of symptoms (days)[a]	Treatment	Outcome
Gutierrez 1996[6]	Case report	63	F	SCr between 1.7-2.8 mg/dl	Rheumatoid arthritis	5	Pancytopenia	-	Blood transfusion, GCSF, leucovorin	Died
Calvo-Romero 2001[7]	Case report	68	M	45ml/min	Rheumatoid arthritis	7.5	Pancytopenia	9	RBC transfusions, GCSF	Died
Arakawa 2019[8]	Case report	78	-	1.8 mg/dL	Psoriasis	7.5	Myelosuppression and AKI	-	Not mentioned	Na
Yoon 2001[9]	Case report	65	M	26 mL/min	Systemic vasculitis	7.5	Pancytopenia and AKI	21	Folinic acid, GCSF	Recovered
Shaikh 2018[10]	Case report	82	F	12 mL/min	Rheumatoid arthritis	7.5	Pancytopenia and AKI	NA	Leucovorin, GCSF, RBC, Platelet, darbepoetin, HD and CVVHFD	Died
Park 1999[11]	Case report	67	F	2.3 mg/dL	Rheumatoid arthritis	5	Pancytopenia and AKI	-	MTX stopped, GCSF, RBC	Recovered
Chen 2017[12b]	Case report	76	M	37 mL/min	Psoriatic arthritis	15	Aplastic anemia	60	Leucovorin,	Recovered
	Case report	82	M	56 mL/min	Psoriasis	35	Thrombocytopenia<br>Neutropenia<br>Oral mucosal ulcers<br>Hepatotoxicity	7	Leucovorin,	Recovered
	Case report	71	M	30 mL/min	Psoriatic arthritis	15	Thrombocytopenia<br>Neutropenia<br>Hepatotoxicity	3	Supportive care	Died
	Case report	73	F	19 mL/min	Psoriasis	5	Pancytopenia, Oral mucosal ulcers	30	Leucovorin, GCSF	Died
	Case report	65	M	19 mL/min	Psoriatic arthritis	10	Thrombocytopenia<br>Neutropenia	3	GCSF	Recovered
	Case report	74	F	41 mL/min	Psoriasis	10	Aplastic anemia	8	Leucovorin	Recovered
	Case report	59	M	59 mL/min	Psoriasis	15	Pancytopenia<br>oral mucosal ulcers	35	Leucovorin	Recovered
	Case report	77	M	52 mL/min	Psoriasis	15	Thrombocytopenia<br>Aplastic anemia<br>oral mucosal ulcers	NA	Leucovorin	Recovered
	Case report	75	F	22 mL/min	Psoriasis	15	Thrombocytopenia,<br>oral mucosal ulcers	NA	Leucovorin	Died
	Case report	49	M	26 mL/min	Psoriasis	5	Aplastic anemia	60	Leucovorin	Recovered

© 2023 Muanda FT et al. *JAMA Network Open.*

Author/year	Design	Age[a]	Sex	eGFR or Cr Cl/ SCr	Indication for LD-MTX use	Dose (mg/week)[a]	Type of toxicity	Onset of symptoms (days)[a]	Treatment	Outcome
	Case report	69	F	33 mL/min	Psoriasis	15	Aplastic anemia<br>Hepatotoxicity	90	Leucovorin	Recovered
	Case report	54	M	40 mL/min	Psoriasis	15	Pancytopenia<br>oral mucosal ulcers	NA	Leucovorin	Recovered
	Case report	68	M	40 mL/min	Rheumatoid arthritis	7.5	Pancytopenia<br>oral and genital mucosal ulcers	90	Leucovorin	Died
	Case report	77	F	49 mL/min	Psoriasis	10	Pancytopenia<br>oral mucosal ulcers<br>Hepatotoxicity	NA	Leucovorin	Recovered
Lim 2005[13b]	Case report	77	F	Renal impairment	NA	7.5	Sepsis	NA	Blood transfusion; Folinic acid	Died
	Case report	78	M	Renal impairment	NA	12.5	Epistaxis	NA	Blood transfusion; Folinic acid	Recovered
	Case report	69	M	Renal impairment	NA	12.5	Epistaxis, sepsis, mucositis, pancytopenia	NA	Blood transfusion; Folinic acid	Recovered
	Case report	82	F	Renal impairment	NA	10	Pneumonia, sepsis, pancytopenia	NA	Blood transfusion; Folinic acid	Recovered
	Case report	82	F	Renal impairment	NA	10	sepsis, pancytopenia	NA	Folinic acid	Recovered
	Case report	61	F	Renal impairment	NA	25	sepsis, epistaxis, mucositis, pancytopenia	NA	Folinic acid, Blood transfusion	Recovered
	Case report	84	F	Renal impairment	NA	10	sepsis, melaena mucositis, pancytopenia	NA	Folinic acid, Blood transfusion	Died
Ajmani 2017[14b]	Case report	65	M	Renal impairment	Psoriasis	25	Pancytopenia, urinary tract infection	NA	GCSF	Died
	Case report	51	F	Renal impairment	Rheumatoid arthritis	15	Pancytopenia	NA	GCSF	Recovered
	Case report	49	M	Renal impairment	Psoriasis	10	Pancytopenia	NA	GCSF	Recovered
	Case report	37	M	Renal impairment	Psoriasis	15	Pancytopenia<br>Pneumotoxicity	NA	GCSF	Recovered
	Case report	59	M	Renal impairment	Psoriasis	15	Pancytopenia<br>Pneumotoxicity	NA	GCSF	Died
	Case report	54	M	Renal impairment	Rheumatoid arthritis	7.5	Pancytopenia<br>Urinary tract infection	NA	GCSF	Died
	Case report	52	M	Renal impairment	Rheumatoid arthritis	10	Pancytopenia<br>Bowel infection	NA	GCSF	Recovered
	Case report	56	F	Renal impairment	Rheumatoid arthritis	NA	Pancytopenia<br>Urinary infection	NA	GCSF	Recovered
	Case report	65	M	Renal impairment	Rheumatoid arthritis	25	Pancytopenia<br>Urinary infection	NA	GCSF	Died
	Case report	48	M	Renal impairment	Rheumatoid arthritis	7.5	Pancytopenia<br>Pneumotoxicity	NA	GCSF	Died

© 2023 Muanda FT et al. *JAMA Network Open.*

Author/year	Design	Age[a]	Sex	eGFR or Cr Cl/ SCr	Indication for LD-MTX use	Dose (mg/week)[a]	Type of toxicity	Onset of symptoms (days)[a]	Treatment	Outcome
	Case report	50	F	Renal impairment	Rheumatoid arthritis	15	Pancytopenia	NA	GCSF	Died
	Case report	45	F	Renal impairment	Rheumatoid arthritis	NA	Pancytopenia	NA	GCSF	Died
	Case report	52	F	Renal impairment	Rheumatoid arthritis	10	Pancytopenia Bowel infection	NA	GCSF	Recovered
**Bergner 2019[15b]**	Case report	66	NA	29 mL/min	Psoriasis	12.5	Leucopenia, mucositis	NA	NA	Recovered
	Case report	75	NA	28 mL/min	Rheumatoid arthritis	15	Pancytopenia, mucositis, pneumonia	NA	NA	Died
	Case report	54	NA	58 mL/min	Rheumatoid arthritis	15	Pancytopenia, mucositis	NA	NA	Recovered
	Case report	82	NA	55 mL/min	Rheumatoid arthritis	15	Pancytopenia, mucositis, fever, esophagitis	NA	NA	Recovered
	Case report	71	NA	59 mL/min	ANCA associated vasculitis	15	Pancytopenia, mucositis, pneumonia	NA	NA	Recovered
	Case report	78	NA	53 mL/min	Rheumatoid arthritis	15	Pancytopenia, pneumonia	NA	NA	Recovered
	Case report	83	NA	51 mL/min	Rheumatoid arthritis	15	Pancytopenia	NA	NA	Recovered
	Case report	78	NA	55 mL/min	Primary Sjoegren's syndrome	15	Pancytopenia	NA	NA	Recovered
	Case report	72	NA	28 mL/min	Psoriasis	22.5	Leucopenia, thrombocytopenia, mucositis	NA	NA	Recovered
	Case report	89	NA	20 mL/min	Rheumatoid arthritis	90	Pancytopenia, mucositis, pneumonia	NA	NA	Died

Abbreviations: CrCl, creatinine clearance; eGFR, estimated glomerular filtration rate; SCr, serum creatinine; LD-MTX, low-dose methotrexate; F, female; M, male; NA, not available; AKI, acute kidney injury; GCSF, granulocyte colony-stimulating factor.

[a]The median [IQR] age, weekly dose, and time to toxicity were 69 (56 to 77) years, 13.75 (8.75 to 15) mg, and 26 (8 to 60) days after methotrexate initiation in these case reports when the information was available.

[b]Case series which included a group of case reports involving patients who were given low dose methotrexate.

© 2023 Muanda FT et al. *JAMA Network Open.*

eTable 4b. Observational studies

Author	Study Size (n)	Study Type and Source Population	Patient Eligibility	Outcomes	Limitations
**Mitsobushi et al. (2021)[16]**	88 patients with CKD<br>5560 patients without CKD	An observational study using the Japanese Adverse Drug Event Report (JADER) database	Oral LD-MTX users with RA were included in this study which described (1) the prevalence of the hematological toxicities in patients with and without CKD. (2) factors associated with hematological toxicities in CKD patients.	A higher prevalence of hematological toxicity among patients with CKD than in those without CKD (37.5% [33/88] vs. 10.7% [594/5560], P<0.001). The folic intake had a preventive effect on the risk of hematological toxicities (Adjusted OR, 0.16 [0.04 to 0.62]).	Reporting bias: For example, pancytopenia may have been preferentially reported because of its severity.<br>Confounding bias: There may be differences in inpatient backgrounds, such as underlying disease.<br>Lack of a comparator group (descriptive cohort study).<br>A small sample of CKD patients.
**Lee et al (2020)[17]**	120 patients who received methotrexate treatment, 66 were included in the newly developed group and 54 were in the previously developed group.	In this retrospective cohort study, the authors reviewed the electronic medical records of patients diagnosed with RA who had reduced renal function between January 1997 and December 2018 at a tertiary referral hospital in Seoul, South Korea.	Patients with RA who were exposed to LD-MTX and in whom the renal function was reduced were included in the study to (1) evaluate LD-MTX -associated toxicity in patients with RA having renal dysfunction receiving LD-MTX treatment and (2) determine the associated risk factors of toxicity.<br>The patients were classified into two groups according to the onset of renal dysfunction: those who newly developed renal dysfunction after LD-MTX treatment for more than 3 months (newly developed group) and those who already had renal dysfunction after they were exposed to LD-MTX (previously developed group)	Thirty-five patients (29.2%) experienced toxicity, and the median time to toxicity events was 23 months (IQR 10–57). The toxicity rate did not differ significantly between newly and previously developed groups [23/66 (34.8%) vs. 12/54 (22.2%), P=0.130]. Multivariate analysis revealed that HCQ use (HR 0.425, 95% CI 0.212–0.853, P=0.016), baseline eGFR (HR 0.938, 95% CI 0.890–0.988, P=0.015) and being female (HR 10.538, 95% CI 1.375–80.793, P=0.023) were associated with LD-MTX -related toxicity.	Lack of an appropriate comparator group: The comparison with a group of patients who developed renal dysfunction after methotrexate initiation may be problematic due to temporal issues.<br>Confounding by indication: The initiation dose of LD-MTX was significantly higher in the newly developed group than in the previously developed group (12.5 vs. 7.5 mg/week, P<0.001).

Abbreviations: CKD, chronic kidney disease; OR, Odd ratio; HR hazard Ratio; CI, confidence interval; LD-MTX, low dose methotrexate; HCQ, hydroxychloroquine

© 2023 Muanda FT et al. *JAMA Network Open.*

eTable 5. Coding definitions for demographic and comorbid conditions

Characteristic	Database	Codes
**Demographics**		
Age	RPDB	
Sex	RPDB	
Location of residence – Rural status	Statistics Canada	
Long-term care	ODB	LTC
Year of cohort entry	ODB	
Socioeconomic Status (Neighbourhood Income Quintile)	Statistics Canada	
LHIN[a]	RPDB	LHIN
Prescriber	ODB	
**Comorbidities (5 years prior the cohort entry date)**		
Acute kidney injury	CIHI-DAD	ICD-10: N17
Anxiety disorder and depression	CIHI-DAD	ICD-10: F063, F064, F204, F313, F314, F315, F32, F33, F341, F400, F401, F402, F408, F409, F410, F411, F412, F413, F418, F419, F420, F421, F422, F428, F429, F430, F431, F432
	OHIP	OHIP DX: 311
	OMHRS (DSM-IV)	29189, 29284, 29289, 29383, 29384, 29620, 29621, 29622, 29623, 29624, 29625, 29626, 29630, 29631, 29632, 29633, 29634, 29635, 29636, 30000, 30001, 30002, 30021, 30022, 30023, 30029, 30030, 30040, 30113
Anemia	CIHI-DAD	ICD10 : D50, D51, D52, D53, D55 , D56 , D570 , D571, D58 , D59, D60 , D61 , D62 , D63, D64
	OHIP	OHIP dx: 280, 281, 282, 283, 284, 285
Bipolar disorder	CIHI-DAD	ICD-10: F300, F301, F302, F308, F309, F310, F311, F312, F313, F314, F315, F316, F317, F318, F319
	OHIP	OHIP DX: 296<br>OHIP FEE: Q020
	OMHRS (DSM-IV)	29600, 29601, 29602, 29603, 29604, 29605, 29606, 29640, 29641, 29642, 29643, 29644, 29645, 29646, 29650, 29651, 29652, 29653, 29654, 29655, 29656, 29660, 29661, 29662, 29663, 29664, 29665, 29666, 29670, 29680, 29689
Myocardial infarction	CIHI-DAD	ICD10: I21, I22
Atrial fibrillation/flutter	CIHI-DAD	ICD10: I48
Cancer	CIHI-DAD	ICD-10: 80003, 80006, 80013, 80023, 80033, 80043, 80102, 80103, 80106, 80113, 80123, 802, 803, 80413, 80423, 80433, 80443, 80453, 80502, 80503, 80513, 80523, 807, 808, 80903, 80913, 80923, 80933, 80943, 80953, 81103, 81202, 81203, 81213, 81223, 81233, 81243, 81303, 81402, 81403, 81406, 81413, 81423, 81433, 81443, 81453, 81473, 81503, 81513, 81523, 81533, 81543, 81553, 81603, 81613, 81623,

© 2023 Muanda FT et al. *JAMA Network Open.*

Characteristic	Database	Codes
		81703, 81713, 81803, 81903, 82003, 82013, 82102, 82103, 82113, 82203, 82213, 823, 82403, 82413, 82433, 82443, 82453, 82463, 82473, 82503, 82513, 82603, 82612, 82613, 82623, 82632, 82633, 82703, 82803, 82813, 82903, 83003, 83103, 83123, 83143, 83153, 83203, 83223, 83233, 83303, 83313, 83323, 83403, 83503, 83703, 83803, 83813, 83903, 84003, 84013, 84103, 84203, 84303, 84403, 84413, 84423, 84503, 84513, 84603, 84613, 84623, 84703, 84713, 84723, 84733, 84803, 84806, 84813, 849, 85002, 85003, 85012, 85013, 85023, 85032, 85033, 85042, 85043, 851, 852, 85303, 854, 85503, 85603, 85623, 857, 85803, 86003, 86203, 86303, 86403, 86503, 86803, 86933, 87003, 87103, 87202, 87203, 87213, 87223, 87233, 87303, 87403, 87412, 87413, 87422, 87423, 87433, 87443, 87453, 87613, 87703, 87713, 87723, 87733, 87743, 87803, 88003, 88006, 88013, 88023, 88033, 88043, 88103, 88113, 88123, 88133, 88143, 88303, 88323, 88333, 88403, 88503, 88513, 88523, 88533, 88543, 88553, 88583, 88903, 88913, 88943, 88953, 88963, 89003, 89013, 89023, 89103, 89203, 89303, 89333, 89403, 89413, 895, 89603, 89633, 89643, 897, 89803, 89813, 89903, 89913, 90003, 90203, 90403, 90413, 90423, 90433, 90443, 90503, 90513, 90523, 90533, 906, 90703, 90713, 90723, 90803, 90813, 90823, 90833, 90843, 90853, 90903, 91003, 91013, 91023, 91103, 91203, 91243, 91303, 91333, 91403, 91503, 91703, 91803, 91813, 91823, 91833, 91843, 91853, 91903, 92203, 92213, 92303, 92313, 92403, 92503, 92513, 92603, 92613, 92703, 92903, 93103, 93303, 93623, 93643, 93703, 93803, 93813, 93823, 93903, 93913, 93923, 940, 941, 942, 94303, 944, 945, 94603, 947, 948, 94903, 95003, 95013, 95023, 95033, 95043, 951, 952, 95303, 95393, 95403, 95603, 95613, 95803, 95813, 959, 965, 966, 967, 968, 969, 970, 971, 972, 973, 97403, 97413, 97603, 97613, 97623, 97633, 97643, 980, 982, 98303, 984, 98503, 986, 98703, 98803, 989, 99003, 99103, 993, 994, C00-C26, C30-C34, C37, C38- C86, C88, C90, C91-C97, D00-D09, Z85
	OHIP	OHIP DX :140-165, 170-175, 179- 208, 230-234
Stroke, including TIA	CIHI-DAD	ICD-10: I62, I630, I631, I632, I633, I634, I635, I638, I639, I64, H341, I600, I601, I602, I603, I604, I605, I606, I607, I609, I61, G450, G451, G452, G453, G458, G459, H340
Chronic liver disease	CIHI-DAD	ICD 10: B16, B17, B18, B19, I85, R17, R18, R160, R162, B942, Z225, E831, E830, K70, K713, K714, K715, K717, K721, K729, K73, K74, K753, K754, K758, K759, K76, K77
	OHIP	OHIP DX: 571, 573, 070<br>OHIP FEE: Z551, Z554
Coronary artery disease, with angina	CIHI-DAD	ICD-10: I20, I21, I22, I23, I24, I25, Z955, Z958, Z959, R931, T822<br>CCI: 1IJ26, 1IJ27, 1IJ54, 1IJ57, 1IJ50, 1IJ76<br>CCP: 4801, 4802, 4803, 4804, 4805, 481, 482, 483
	OHIP	OHIP DX: 410, 412, 413<br>OHIP FEE: R741, R742, R743, G298, E646, E651, E652, E654, E655, G262, Z434, Z448
Congestive heart failure	CIHI-DAD	ICD-10: I099, I420, I425, I426, I427, I428, I429, I43, I500, I501, I509, I255, J81<br>CCP: 4961, 4962, 4963, 4964<br>CCI: 1HP53, 1HP55, 1HZ53GRFR, 1HZ53LAFR, 1HZ53SYFR
	OHIP	OHIP DX: 428

© 2023 Muanda FT et al. *JAMA Network Open.*

Characteristic	Database	Codes
		OHIP FEE: R701, R702, Z429
Epilepsy/seizure	CIHI-DAD	ICD-10: G40, G41, R5680, R5688
	OHIP	OHIP DX: 345, 780
Migraine	CIHI-DAD	ICD-10: G43
	OHIP	OHIP DX: 346
Rheumatoid Arthritis	CIHI-DAD	ICD10: M05, M06
	OHIP	OHIP Dx: 714
Dyslipidemia	CIHI-DAD	ICD-10: E78
	OHIP	OHIP DX: 272
Crohn disease	CIHI-DAD	ICD10: "K50"
	OHIP	OHIP DX: 555
Parkinson's disease	CIHI-DAD	ICD-9: 332<br>ICD-10: G20, F023
Peripheral vascular disease	CIHI-DAD	ICD 10: I700, I702, I708, I709, I731, I738, I739, K551<br>CCP: 5125, 5129, 5014, 5016, 5018, 5028, 5038, 5126, 5159<br>CCI: 1KA76, 1KA50, 1KE76, 1KG50, 1KG57, 1KG76MI, 1KG87, 1IA87LA, 1IB87LA, 1IC87LA, 1ID87, 1KA87LA, 1KE57
	OHIP	OHIP FEE: R787, R780, R797, R804, R809, R875, R815, R936, R783, R784, R785, E626, R814, R786, R937, R860, R861, R855, R856, R933, R934, R791, E672, R794, R813, R867, E649
Ulcerative colitis (UC)	CIHI-DAD	ICD10: K51
	OHIP	OHIP DX: 556
Osteortathritis	CIHI-DAD	ICD10: M15, M16, M17, M18, M19, M47
Psoriasis	CIHI-DAD	ICD10: L400, L401, L404, L405, L408, L409, L413, L414, L418, L415, L419, M070, M072, M073, M090
	OHIP	OHIP DX: 696
Sarcoidosis	CIHI-DAD	ICD10: D860, D861, D862, D863, D868, D869, G532, M633
	OHIP	OHIP DX: 135
Dermatomyositis	CIHI-DAD	ICD10: M330, M331
	OHIP	OHIP DX:710
Systemic sclerosis	CIHI-DAD	ICD10:L940, L941, M342, M340, M341, M348, M349
	OHIP	OHIP DX:701
Atopic dermatitis or eczema	CIHI-DAD	ICD10: L208, L209, B000
	OHIP	OHIP DX: 691
Gout	CIHI-DAD	ICD10: M10
	OHIP	OHIP dx: 274
Hypercalcemia	CIHI-DAD	ICD10: E835

© 2023 Muanda FT et al. *JAMA Network Open.*

Characteristic	Database	Codes
Diabetes	ODB	Insulins, oral antihyperglycemic agents
Hypertension	ODB	Antihypertensive agents
Ventricular arrythmia	CIHI-DAD	ICD10: I4900, I472
Dementia	CIHI-DAD	ICD10: F065, F066, F068, F069, F09, F00, F01, F02, F03, F051, G30, G31, R54
	OHIP	OHIP DX CODES: "290", "331", "797"
	OMHRS (DSM-IV)	DSM-IV (OMHRS): "29040", "29041", "29042", "29043", "29120", "29282", "29410", "29411", "29480", "7809
Hyponatremia	CIHI-DAD	ICD10: E871
Schizophrenia	CIHI-DAD	ICD-10: F060, F062, F105, F107, F115, F117, F125, F127, F135, F137, F145, F147, F155, F157, F165, F167, F175, F177, F185, F187, F195, F197, F200, F201, F202, F203, F204, "F205", "F206, F208, F209, F220, F228, F229, F230, F231, F232, F233, F238, F239, F24, F250, F251, F252, F258, F259, F28, F29
	OHIP	OHIP dx: 291, 292, 295, 297, 298<br>OHIP fee: Q021
	OMHRS (DSM-IV)	DSM-IV (OMHRS): 29130, 29150, 29211, 29212, 29381, 29382, 29510, 29520, 29530, 29540, 29560, 29570, 29590, 29710, 29730, 29880, 29890
Alcohol misuse	CIHI-DAD	ICD10: E24, E512, F10, G312, G621, G721, I426, K292, K70, K860, T510, X45, X65, Y15, Y573, Z502, Z714, Z721
	OHIP	OHIP dx: 303
hypotension	CIHI-DAD	ICD10: I95
Arrythmia	CIHI-DAD	ICD10: I48, I44, I45, I47, I4900, I4901, I491, I492, I493, I494, I498, I499, R000<br>R001<br>OHIP fee: G178, G179, G249, G261, G259, Z443, Z431 Z437
Urinary tract infection	CIHI-DAD	ICD10: N10, N11, N12, N136, N151, N159, N160, N300, N308, N309, N340, N390, N410, N411, N412, N413, N431, N45, T835
Community-acquired pneumonia	CIHI-DAD	ICD10: J12, J13, J14, J15, J16, J17, J18, P23
Prosthetic joint infection	CIHI-DAD	ICD-10: T845
	OHIP	OHIP dx: 739
Other bacterial infections	CIHI-DAD	ICD-10: A49
	OHIP	OHIP dx: 786, 136, 040, 039
Gallstones /biliary stones	CIHI-DAD	ICD10: K80, K81, K82, K83, K87, K862, K863, K868, K869
	OHIP	OHIP DX: 574, 575, 576
Sepsis	CIHI-DAD	ICD10: A021, A392, A393, A394, A400, A401, A402, A408, A409, A410, A411, A412, A403, A414, A4159, A413, A4150, A4151, A4152, A4158, A4180" A4188, A427, A419

© 2023 Muanda FT et al. *JAMA Network Open.*

Characteristic	Database	Codes
Chronic obstructive pulmonary disease	CIHI-DAD	ICD10: J41, J43, J44
Gastroesophageal reflux disease	CIHI-DAD	ICD10: K21
	OHIP	OHIP dx: 530, 531, 532, 533, 534, 535, 536, 537, 538 , 539
Glaucoma	CIHI-DAD	ICD-10: H40<br>CCP: 0926
	OHIP	OHIP fee: "E123", "E133", "E214", "E983", "E984", "G819", "G820"
Cataract	CIHI-DAD	ICD-10 H25, H26, H27, H28
	OHIP	OHIP fee: E214, E140, E141
Syncope	CIHI-DAD	ICD10: R55
Prostate cancer	CIHI-DAD	ICD10: C61, D075
	OHIP	OHIP DX: 185
Prostatitis	CIHI-DAD	ICD10: N410, N411, N412
	OHIP	OHIP DX: 601
Prostatic hyperplasia	CIHI-DAD	ICD10: N40
	OHIP	OHIP DX: 600
Macular degeneration	CIHI-DAD	ICD-10 H35
	OHIP	OHIP Fee: E154, E125, E126, E1
Obesity	CIHI-DAD	ICD10: E660, E661, E662, E668, E669
	OHIP	OHIP DX: 278
Inflammatory bowel disease	CIHI-DAD	ICD10: "K50", "K51"
Hypothyroidism	CIHI-DAD	ICD-10: E030, E031, E032, E033, E034, E035, E038, E039, E890
	OHIP	OHIP DX: 243, 244
Hypoglycemia	CIHI-DAD	ICD10: E15, E160, E161, E162, E1063, E1163, E1363, E1463
Cirrhosis/liver damage	CIHI-DAD	ICD10: K702, K703, K704, K709, K740, K741, K742, K743, K744, K745, K746
	OHIP	OHIP DX: 571, 573
Chronic lung disease	CIHI-DAD	ICD10: I272, I278, I279, J40, J41, J42, J43, J44, J45, J47, J60, J61, J62, J63, J64, J65, J66, J67, J68, J701, J703, J704, J708, J709, J82, J84, J92, J941, J949, J953, J961, J969, J984, "J988", J989, J99
	OHIP	OHIP DX: 491, 492, 493, 494, 496, 501, 502, 515, 518, 519<br>OHIP FEE: J889, J689
Hypokalemia	CIHI-DAD	ICD10: E876
	OHIP	OHIP dx: 579

© 2023 Muanda FT et al. *JAMA Network Open.*

Characteristic	Database	Codes
Urinary retention	CIHI-DAD	ICD10: R33
**Medication use (120 days before cohort entry)**		
All medications	ODB	
**Healthcare Use (1 year prior to the cohort entry date)**		
GP/FP visits	OHIP<br>IPDB	Mainspeciality = “GP/FP” or “F.P./EMERGENCY MEDICINE”
Number of any hospitalizations	CIHI-DAD	"ddate"
Number of any ER visits	NACRS	"regdate"
TSH	OHIP	OHIP FEE: G016, L341
CT head	OHIP	OHIP FEE: X188, X400, X401, X402, X405, X408
CT abdomen	OHIP	OHIP FEE: X126, X409, X410
CT extremities	OHIP	OHIP FEE: X127, X412, X413
CT neck	OHIP	OHIP FEE: X124, X403, X404
CT pelvis	OHIP	OHIP FEE: X128, X415, X416
CT spine	OHIP	OHIP FEE: X231, X232, X233
CT thorax	OHIP	OHIP FEE: X125, X406, X407
Chest x-ray	OHIP	OHIP FEE: X090, X091, X092, X195
Echocardiography	CIHI-DAD	CCP: 0282<br>CCI: 3IP30
	OHIP	OHIP FEE: G560, G561, G562, G566, G567, G568, G570, G571, G572, G574, G575, G576, G577, G578, G581
Carotid ultrasound	CIHI-DAD	CCP: 0281<br>CCI: 3JE30, 3JG30
	OHIP	OHIP FEE: J201, J501, J190, J191, J490, J491, J492
Cardiac catheterization	CIHI-DAD	CCP: 4995, 4996, 4997, 4892, 4893, 4894, 4895, 4896, 4897, 4898<br>CCI: 3IJ30GP, 3HZ30GP, 2HZ24GPKJ, 2HZ24GPKL, 2HZ24GPKM, 2HZ24GPXJ, 2HZ28GPPL, 2HZ71GP, 3IP10, 3IS10
	OHIP	OHIP FEE: G296, G297, G299, G300, G301, G304, G305, G306, G297, G509
Coronary angiogram	CIHI-DAD	CCP: 4892, 4893, 4894, 4895, 4896, 4897, 4898<br>CCI: 3IP10, 3IS10
	OHIP	OHIP FEE: G297, G509
Holter monitoring	CIHI-DAD	CCP: 0354<br>CCI: 2HZ24JAKH
	OHIP	OHIP FEE: G311, G320, G647, G648, G649, G650, G651, G652, G653, G654, G655, G656, G657, G658, G659, G660, G661, G682, G683, G684, G685, G686, G687, G688, G689, G690, G692, G693

© 2023 Muanda FT et al. *JAMA Network Open.*

Characteristic	Database	Codes
Cardiac stress test	CIHI-DAD	CCP: 0341, 0342, 0343, 0344, 0605<br>CCI: 2HZ08, 3IP70
	OHIP	OHIP FEE: G315, G174, G111, G112, G319, G582, G583, G584, J607, J608, J807, J808, J809, J866, J609, J666
Coronary revascularization	CIHI-DAD	CCP: 481, 482, 483, 480<br>CCI: 1IJ50, 1IJ26, IIJ27, 1IJ57, 1IJ76, 1IJ57GQ, 1IJ54GQAZ
	OHIP	OHIP FEE: R741, R742, R743, E651, E652, E654, E646, G298, Z434, G262
Electrocardiography	CIHI-DAD	CCI: 2HZ24JAKE
	OHIP	OHIP FEE: G310, G313
Colorectal cancer screening	OHIP	OHIP FEE: G004, L179, L181, Q043, Q152, X112, X113, Z535, Z536, Z555, Z580
Cervical cancer screening	OHIP	OHIP FEE: E430, G365, G394, L713, L812
Prostate-specific antigen test	OHIP	OHIP FEE: Q005, Q118, Q119, Q120, Q121, Q122, Q123, Q133
Mammography	OHIP	OHIP FEE: X172, X178, X184, X185, X201
Influenza vaccination	OHIP	OHIP FEE: G590, G591
Bone mineral density test	OHIP	OHIP FEE: J654, J688, J854, J888, X149, X152, X153, X155, Y654, Y688, Y854, Y888
Hearing test	OHIP	OHIP FEE: G153, G154, G440, G441, G442, G443, G448, G450, G451, G452, G525, G526, G529, G530, G533, G815, G816
Cystoscopy	OHIP	OHIP FEE: Z606, Z607, Z628, Z632, Z633, Z634
Pulmonary function test	OHIP	OHIP FEE: L354, L358
At-home physician service	OHIP	OHIP FEE: A901, B960, B961, B962, B963, B964, B966, B990, B992, B993, B994, B996, B997, B998
Urinalysis	OHIP	OHIP FEE: L253, L254, L255, L633, G009, G010
Serum creatinine value*	OLIS	OBSERVATIONCODE: 14682-9
Urine albumin-to-creatinine ratio*	OLIS	OBSERVATIONCODE: 14959-1, 30000-4,32294-1, XON10383-8 and XON12394-3

*Assessed in the 365-day period before the cohort entry date.

© 2023 Muanda FT et al. *JAMA Network Open.*

eTable 6. Operating characteristics of hospital diagnosis codes used to define the primary and secondary outcomes

Outcome	Outcome component	ICD-10/CCI/OHIP/ORGD codes in this study	ICD-10 codes used in the validation study	Reference Standard	Operating Characteristics, % (95% CI)			Study
					Sensitivity	Specificity	Positive predictive value	
**Hospital visit with myelosuppression** [a]	Aplastic anemia/pancytopenia	ICD-10 codes: D611, D612, D613, D619, D618	ICD-10 codes D611	Identification of aplastic anemia in the review of the medical charts of four German hospitals covering the years 2014 and 2015 was done by experienced nurses, pharmacists, and hospital medical coders from the participating hospitals after having completed a 1-month training phase.	NA	NA	74 (60- 85)	**Kuklik**2017[18]
	Neutropenia	ICD-10 code: D700, D708, D729	NA	NA	NA	NA	NA	
	Thrombocytopenia	ICD-10 codes: D6938, D696	ICD-10 codes D693	Information from the chart reviews was used to identify patients with chronic idiopathic thrombocytopenic purpura (ITP). In addition to requiring an ITP diagnosis recorded in the medical charts for a period of more than 6 months, a platelet count below 150 × 10^9/L was required to confirm the diagnosis	NA	NA	93 (91-96)	Heden KE 2009[19]

© 2023 Muanda FT et al. *JAMA Network Open.*

Outcome	Outcome component	ICD-10/CCI/OHIP/ORGD codes in this study	ICD-10 codes used in the validation study	Reference Standard	Operating Characteristics, % (95% CI)			Study
					Sensitivity	Specificity	Positive predictive value	
**Hospital visit with pneumotoxicity**		ICD-10 codes: J12, J13, J14, J15, J16, J17, J18, P23	ICD-10 codes: J12, J13, J14, J15, J16, J17, J18,	Chart review was used to identify patients with pneumonia using relevant signs and symptoms consistent with a diagnostic of pneumonia: the documentation of cough, sputum production, pleuritic chest pain, fever [37.5 degrees Celsius], shortness of breath, crackles [crepitations], and aspiration	98 (97-98)	97(96-98)	96 (95-97)	Skull SA 2008[20]
**Hospital visit with hepatotoxicity**		ICD 10 codes: K711, K716, K719, K720, K729, K759	ICD-10 codes: K711, K716, K719, K720, K729	Chart review was used to identify patients with hepatoxicity and acute liver failure using predefined criteria. Hepatotoxicity was defined by Alanine aminotransferase >1,000 U/L, and acute liver injury was defined by an	86 (65 -97)	96 (79 - 100)	95 (75-100)	Myers RP 2007[21]

© 2023 Muanda FT et al. *JAMA Network Open.*

Outcome	Outcome component	ICD-10/CCI/OHIP/ORGD codes in this study	ICD-10 codes used in the validation study	Reference Standard	Operating Characteristics, % (95% CI)			Study
					Sensitivity	Specificity	Positive predictive value	
				international normalized ratio (INR) > 1.5				
**Hospital visit with sepsis**		ICD-10 codes: A021, A392, A393, A394, A400, A401, A402, A408, A409, A410, A411, A412, A403, A414, A4159, A413, A4150, A4151, A4152, A4158, A4180, A4188, A427, A419	ICD-10 codes: A021, A392, A393, A394, A400, A401, A402, A408, A409, A410, A411, A412, A403, A414, A4159, A413, A4150, A4151, A4152, A4158, A4180, A4188, A427, A419	Chart review was reviewed by two independent physicians (one trained in intensive care medicine and the other in surgery) to identify cases of sepsis using a checklist criteria tool developed based on the ACCP/SCCM 2001 Consensus Conference updated definitions and consensus of clinical experts.	46 (42- 50)	99 (97-100)	98 (96-99)	Jolley RJ, 2015[22]
**Death**					98	100		Jha P 1996[23]

Abbreviations: ICD-10, International Classification of Diseases, Tenth Revision; NA, not available

[a]The algorithm used to identify myelosuppression includes a diagnosis of aplastic anemia/pancytopenia neutropenia or thrombocytopenia. ICD-10 code algorithms that captured neutropenia were not validated. Despite moderate to high positive predictive values for algorithms detecting aplastic anemia and thrombocytopenia, neither sensitivity nor specificity was available.

© 2023 Muanda FT et al. *JAMA Network Open.*

eTable 7. Variables included in the propensity score model

Category	Variables
**Demographics**	Age, sex, year of cohort entry, neighborhood income quintile, long-term residence, location, Local Health Integration Network, prescriber type
**Comorbidities**	Acute kidney injury, alcoholism, angina, atrial fibrillation/flutter, bipolar disorder, chronic liver disease, chronic obstructive pulmonary disease, cirrhosis, coronary artery disease (minus angina), dementia, diabetes, anemia, glaucoma, major hemorrhage, congestive heart failure, hypertension, hypokalemia, hyponatremia, hypothyroidism, migraine, acute myocardial infarction, obesity, Parkinson disease, peripheral vascular disease, schizophrenia, hypoglycemia, ischemic stroke, unipolar depression and/or anxiety disorder, rheumatoid arthritis, syncope, arrhythmia, inflammatory bowel disease, cancer, prostatic hyperplasia, fracture, fall, hyperkalemia, prostatitis, hypotension, ulcerative colitis, Crohn disease, acute urinary retention, macular degeneration, dyslipidemia, gastroesophageal reflux disease, major surgery, osteoarthritis, gallstones /biliary stones, gout, prior pneumotoxicity, prior myelotoxicity, prior methotrexate toxicity, psoriasis, dermatomyositis, sarcoidosis, systemic sclerosis, systemic lupus erythematosus, eczema, urinary tract infection, prior sepsis, modified Charlson comorbidity index
**Medications**	Alpha-adrenergic blocking agents, anti-arrhythmic, allopurinol, antibiotics, anticoagulants, angiotensin II receptor blockers, Ace inhibitors, antifungals, Tricyclic antidepressants, gastrointestinal drugs, beta-blockers, H2 blockers, anticonvulsants, aspirin, antiplatelet agents, bone calcium regulators, benzodiazepine, bisphosphonates, beta-agonists, calcium channel blockers, cholinesterase inhibitors, glucocorticoid, loop diuretics, nitrates, fibrates, NSAIDs (excluding aspirin), insulin, potassium-sparing diuretics, opioids, overactive bladder medication, antipsychotics, proton pump inhibitors, 5 alpha reductases, selective serotonin reuptake inhibitors, thiazide diuretics, oral antidiabetics, statins, number of unique drug names, number of unique dins
**Health Care Use**	Emergency department visits, family physician visits, hospitalization
**Investigations**	TSH test, at-home, physician service, Bone mineral density test, cardiac catheterization, cardiac stress test, carotid ultrasound, chest-X ray, cataract surgery, cervical cancer screening, colorectal cancer screening, cholesterol test (total cholesterol, HDL), CT abdomen, CT extremities, CT head, CT neck, CT pelvis, CT spine, CT thorax, echocardiography, flu shot, cystoscopy, hearing test, mammography, prostate-specific antigen (PSA) test, Holter monitoring, parathyroid hormone testing, pulmonary function test, urinalysis, eGFR value

Abbreviations: ACR, urine albumin-to-creatinine ratio; ACE inhibitor, angiotensin-converting-enzyme inhibitor; CT, computed tomography; eGFR, estimated glomerular filtration rate; H2 blockers, Histamine H2-receptor antagonists.

© 2023 Muanda FT et al. *JAMA Network Open.*

eTable 8. Baseline characteristics[a] of older adults with chronic kidney disease newly prescribed low-dose methotrexate (LD MTX) vs those newly prescribed hydroxychloroquine (HCQ) in Ontario, Canada (2008–2021)

		Unmatched data (N=6909)					Matched data (N=4618) [b]				
		LD-MTX (n=2900)		HCQ (n=4009)		Standardized. Difference[c]	LD-MTX (n=2309)		HCQ (n=2309)		Standardized. Difference[c]
**Demographics**											
Age at cohort entry	Mean ± SD	77.2	6.9	76.9	6.8	5%	77.1	6.9	77.1	6.9	0%
	Median (IQR)	77	(72-82)	76	(71-82)	.	77	(72-82)	77	(71-82)	.
	66-<70	474	16.3%	673	16.8%	1%	368	15.9%	377	16.3%	1%
	70-<75	649	22.4%	947	23.6%	3%	529	22.9%	531	23.0%	0%
	75-<80	703	24.2%	982	24.5%	1%	569	24.6%	540	23.4%	3%
	80-<85	582	20.1%	791	19.7%	1%	459	19.9%	466	20.2%	1%
	85-<90	370	12.8%	468	11.7%	3%	291	12.6%	300	13.0%	1%
	90+	122	4.2%	148	3.7%	3%	93	4.0%	95	4.1%	1%
sex	F	1918	66.1%	2958	73.8%	17%	1611	69.8%	1581	68.5%	3%
	M	982	33.9%	1051	26.2%	17%	698	30.2%	728	31.5%	3%
Year of cohort entry	2008	81	2.8%	101	2.5%	2%	69	3.0%	58	2.5%	3%
	2009	199	6.9%	238	5.9%	4%	155	6.7%	152	6.6%	0%
	2010	227	7.8%	251	6.3%	6%	172	7.4%	173	7.5%	0%
	2011	230	7.9%	276	6.9%	4%	181	7.8%	187	8.1%	1%
	2012	236	8.1%	246	6.1%	8%	183	7.9%	175	7.6%	1%
	2013	216	7.4%	323	8.1%	3%	177	7.7%	168	7.3%	2%
	2014	211	7.3%	298	7.4%	0%	165	7.1%	165	7.1%	0%
	2015	184	6.3%	308	7.7%	5%	144	6.2%	150	6.5%	1%
	2016	206	7.1%	275	6.9%	1%	162	7.0%	149	6.5%	2%

© 2023 Muanda FT et al. *JAMA Network Open.*

		Unmatched data (N=6909)					Matched data (N=4618)[b]				
		LD-MTX (n=2900)		HCQ (n=4009)		Standardized. Difference[c]	LD-MTX (n=2309)		HCQ (n=2309)		Standardized. Difference[c]
	2017	218	7.5%	331	8.3%	3%	176	7.6%	185	8.0%	1%
	2018	243	8.4%	375	9.4%	4%	195	8.4%	210	9.1%	2%
	2019	250	8.6%	377	9.4%	3%	200	8.7%	187	8.1%	2%
	2020	214	7.4%	355	8.9%	5%	171	7.4%	184	8.0%	2%
	2021	185	6.4%	255	6.4%	0%	159	6.9%	166	7.2%	1%
Location	Urban	2517	86.8%	3513	87.6%	2%	2017	87.4%	2011	87.1%	1%
	Rural	383	13.2%	496	12.4%	2%	292	12.6%	298	12.9%	1%
Residence	Long term care	42	1.4%	37	0.9%	5%	28	1.2%	30	1.3%	1%
LHIN	1	159	5.5%	164	4.1%	7%	113	4.9%	125	5.4%	2%
	2	238	8.2%	267	6.7%	6%	187	8.1%	184	8.0%	0%
	3	178	6.1%	232	5.8%	1%	138	6.0%	133	5.8%	1%
	4	472	16.3%	871	21.7%	14%	422	18.3%	413	17.9%	1%
	5	191	6.6%	212	5.3%	5%	160	6.9%	145	6.3%	2%
	6	185	6.4%	239	6.0%	2%	152	6.6%	157	6.8%	1%
	7	139	4.8%	205	5.1%	1%	110	4.8%	114	4.9%	0%
	8	242	8.3%	426	10.6%	8%	206	8.9%	217	9.4%	2%
	9	289	10.0%	394	9.8%	1%	233	10.1%	226	9.8%	1%
	10	185	6.4%	151	3.8%	12%	118	5.1%	125	5.4%	1%
	11	360	12.4%	442	11.0%	4%	257	11.1%	267	11.6%	2%
	12	79	2.7%	180	4.5%	10%	69	3.0%	66	2.9%	1%
	13	127	4.4%	169	4.2%	1%	102	4.4%	98	4.2%	1%
	14	56	1.9%	57	1.4%	4%	42	1.8%	39	1.7%	1%

© 2023 Muanda FT et al. *JAMA Network Open.*

		Unmatched data (N=6909)					Matched data (N=4618)[b]				
		LD-MTX (n=2900)		HCQ (n=4009)		Standardized. Difference[c]	LD-MTX (n=2309)		HCQ (n=2309)		Standardized. Difference[c]
Socio-economic status[d]	1	566	19.5%	781	19.5%	0%	431	18.7%	433	18.8%	0%
	2	630	21.7%	874	21.8%	0%	507	22.0%	484	21.0%	2%
	3	619	21.3%	835	20.8%	1%	489	21.2%	502	21.7%	1%
	4	546	18.8%	780	19.5%	2%	441	19.1%	465	20.1%	3%
	5	539	18.6%	739	18.4%	1%	441	19.1%	425	18.4%	2%
**Prescriber**											
Rheumatologist		1540	53.1%	2471	61.6%	17%	1354	58.6%	1390	60.2%	3%
General practicioner		428	14.8%	619	15.4%	2%	347	15.0%	338	14.6%	1%
Internist		210	7.2%	296	7.4%	1%	184	8.0%	181	7.8%	1%
Dermatologist		321	11.1%	116	2.9%	33%	104	4.5%	102	4.4%	0%
Other		158	5.4%	180	4.5%	4%	115	5.0%	110	4.8%	1%
Missing		243	8.4%	327	8.2%	1%	205	8.9%	188	8.1%	3%
**Comorbidities[e]**											
Acute kidney injury		204	7.0%	249	6.2%	3%	156	6.8%	152	6.6%	1%
Alcoholism		30	1.0%	45	1.1%	1%	23	1.0%	23	1.0%	0%
Angina		490	16.9%	686	17.1%	1%	383	16.6%	373	16.2%	1%
Atrial fibrillation/flutter		199	6.9%	280	7.0%	0%	162	7.0%	164	7.1%	0%
Bipolar disorder		53	1.8%	83	2.1%	2%	37	1.6%	36	1.6%	0%
Chronic liver disease		107	3.7%	201	5.0%	6%	93	4.0%	91	3.9%	1%
Chronic obstructive pulmonary disease		863	29.8%	1203	30.0%	0%	691	29.9%	697	30.2%	1%
Cirrhosis		70	2.4%	141	3.5%	7%	63	2.7%	62	2.7%	0%

© 2023 Muanda FT et al. *JAMA Network Open.*

	Unmatched data (N=6909)					Matched data (N=4618)[b]				
	LD-MTX (n=2900)		HCQ (n=4009)		Standardized. Difference[c]	LD-MTX (n=2309)		HCQ (n=2309)		Standardized. Difference[c]
Coronary artery disease (minus angina)	820	28.3%	1105	27.6%	2%	646	28.0%	631	27.3%	2%
Dementia	269	9.3%	329	8.2%	4%	202	8.7%	194	8.4%	1%
Anemia	864	29.8%	1201	30.0%	0%	679	29.4%	690	29.9%	1%
Glaucoma	324	11.2%	476	11.9%	2%	258	11.2%	272	11.8%	2%
Major hemorrhage	184	6.3%	235	5.9%	2%	144	6.2%	156	6.8%	2%
Congestive heart failure	481	16.6%	668	16.7%	0%	384	16.6%	382	16.5%	0%
Hypertension	2329	80.3%	3303	82.4%	5%	1865	80.8%	1866	80.8%	0%
Hypokalemia	66	2.3%	76	1.9%	3%	48	2.1%	38	1.6%	4%
Hyponatremia	55	1.9%	77	1.9%	0%	43	1.9%	37	1.6%	2%
Hypothyroidism	394	13.6%	574	14.3%	2%	320	13.9%	328	14.2%	1%
Migraine	87	3.0%	153	3.8%	4%	69	3.0%	65	2.8%	1%
Obesity	170	5.9%	256	6.4%	2%	136	5.9%	136	5.9%	0%
Parkinson disease	45	1.6%	47	1.2%	3%	30	1.3%	32	1.4%	1%
Peripheral vascular disease	37	1.3%	47	1.2%	1%	27	1.2%	28	1.2%	0%
Schizophrenia	52	1.8%	67	1.7%	1%	40	1.7%	39	1.7%	0%
Ischaemic stroke	47	1.6%	51	1.3%	3%	32	1.4%	32	1.4%	0%
Depression	224	7.7%	364	9.1%	5%	187	8.1%	171	7.4%	3%
Rheumatoid arthritis	1500	51.7%	1922	47.9%	8%	1271	55.0%	1308	56.6%	3%
Syncope	50	1.7%	44	1.1%	5%	34	1.5%	30	1.3%	2%
Arrhythmia	282	9.7%	392	9.8%	0%	226	9.8%	230	10.0%	1%
Inflammatory bowel disease	51	1.8%	33	0.8%	9%	29	1.3%	27	1.2%	1%

© 2023 Muanda FT et al. *JAMA Network Open.*

	Unmatched data (N=6909)					Matched data (N=4618)[b]				
	LD-MTX (n=2900)		HCQ (n=4009)		Standardized. Difference[c]	LD-MTX (n=2309)		HCQ (n=2309)		Standardized. Difference[c]
Major cancer[f]	1171	40.4%	1617	40.3%	0%	943	40.8%	930	40.3%	1%
Prostatic hyperplasia	310	10.7%	364	9.1%	5%	223	9.7%	243	10.5%	3%
Fracture	213	7.3%	313	7.8%	2%	175	7.6%	178	7.7%	0%
Falls	141	4.9%	187	4.7%	1%	109	4.7%	102	4.4%	1%
Hyperkalemia	26	0.9%	46	1.1%	2%	25	1.1%	23	1.0%	1%
Prostatitis	61	2.1%	75	1.9%	1%	46	2.0%	48	2.1%	1%
Hypotension	65	2.2%	65	1.6%	4%	43	1.9%	45	1.9%	0%
Gallstones /biliary stones	144	5.0%	197	4.9%	0%	112	4.9%	113	4.9%	0%
Prior pneumotoxcity	142	4.9%	173	4.3%	3%	115	5.0%	109	4.7%	1%
Prior myelotoxicity	36	1.2%	41	1.0%	2%	25	1.1%	19	0.8%	3%
Prior sepsis	49	1.7%	58	1.4%	2%	37	1.6%	36	1.6%	0%
Prior methotrexate toxicity	196	6.8%	250	6.2%	2%	157	6.8%	147	6.4%	2%
Psoriasis	448	15.4%	130	3.2%	43%	132	5.7%	127	5.5%	1%
Dermatomyositis	191	6.6%	546	13.6%	23%	182	7.9%	184	8.0%	0%
Sarcoidosis	30	1.0%	24	0.6%	4%	20	0.9%	23	1.0%	1%
Systemic sclerosis or scleroderma	221	7.6%	305	7.6%	0%	172	7.4%	170	7.4%	0%
Systemic lupus erythematosus	264	9.1%	664	16.6%	23%	236	10.2%	238	10.3%	0%
Atopic dermatitis or eczema	918	31.7%	1154	28.8%	6%	642	27.8%	655	28.4%	1%
Hypoglycemia	18	0.6%	34	0.8%	2%	14	0.6%	16	0.7%	1%
Ulcerative colitis (UC)	60	2.1%	56	1.4%	5%	44	1.9%	36	1.6%	2%

© 2023 Muanda FT et al. *JAMA Network Open.*

		Unmatched data (N=6909)					Matched data (N=4618)[b]				
		LD-MTX (n=2900)		HCQ (n=4009)		Standardized. Difference[c]	LD-MTX (n=2309)		HCQ (n=2309)		Standardized. Difference[c]
Crohn disease		57	2.0%	36	0.9%	9%	33	1.4%	32	1.4%	0%
Acute urinary retention		61	2.1%	71	1.8%	2%	39	1.7%	40	1.7%	0%
Myocardial infarction		107	3.7%	122	3.0%	4%	74	3.2%	67	2.9%	2%
Dyslipidemia		744	25.7%	1009	25.2%	1%	574	24.9%	563	24.4%	1%
Macula degeneration		149	5.1%	132	3.3%	9%	100	4.3%	108	4.7%	2%
Gastroesophageal reflux disease		738	25.4%	1101	27.5%	5%	606	26.2%	600	26.0%	0%
Osteoarthritis		284	9.8%	475	11.8%	6%	237	10.3%	233	10.1%	1%
Major surgery		171	5.9%	229	5.7%	1%	130	5.6%	122	5.3%	1%
Prostate cancer		107	3.7%	115	2.9%	4%	80	3.5%	80	3.5%	0%
Diabete		732	25.2%	990	24.7%	1%	571	24.7%	572	24.8%	0%
Urinary tract infection		200	6.9%	262	6.5%	2%	157	6.8%	154	6.7%	0%
Gout		418	14.4%	610	15.2%	2%	330	14.3%	342	14.8%	1%
Modified Charlson comorbidity index[g]	Mean ± SD	2.53	1.23	2.45	1.13	7%	2.5	1.2	2.49	1.19	1%
	Median (IQR)	2	(2-2)	2	(2-2)	.	2	(2-2)	2	(2-2)	.
	2	2271	78.3%	3218	80.3%	5%	1831	79.3%	1835	79.5%	0%
	3+	629	21.7%	791	19.7%	5%	478	20.7%	474	20.5%	0%
**Medication use[h] , No. (%)**											
Alpha adrenergic blocking agents		91	3.1%	133	3.3%	1%	69	3.0%	73	3.2%	1%
Anti-arythmic		39	1.3%	39	1.0%	3%	30	1.3%	31	1.3%	0%
Antibiotics		861	29.7%	1127	28.1%	4%	673	29.1%	665	28.8%	1%
Ace inhibitor		839	28.9%	1183	29.5%	1%	676	29.3%	691	29.9%	1%

© 2023 Muanda FT et al. *JAMA Network Open.*

	Unmatched data (N=6909)					Matched data (N=4618)[b]				
	LD-MTX (n=2900)		HCQ (n=4009)		Standardized. Difference[c]	LD-MTX (n=2309)		HCQ (n=2309)		Standardized. Difference[c]
Anticoagulants	356	12.3%	504	12.6%	1%	289	12.5%	297	12.9%	1%
Anticonvulsants	236	8.1%	405	10.1%	7%	192	8.3%	206	8.9%	2%
Angiotensin II receptor blockers	936	32.3%	1379	34.4%	4%	755	32.7%	767	33.2%	1%
Aromatase inhibitors	26	0.9%	25	0.6%	3%	24	1.0%	10	0.4%	7%
Aspirin	65	2.2%	91	2.3%	1%	51	2.2%	48	2.1%	1%
Antiplatele**et** agents	170	5.9%	275	6.9%	4%	129	5.6%	133	5.8%	1%
Antifungals	54	1.9%	73	1.8%	1%	41	1.8%	39	1.7%	1%
Tricyclic antidepressant	416	14.3%	676	16.9%	7%	323	14.0%	326	14.1%	0%
Gastrointestinal drugs	164	5.7%	191	4.8%	4%	123	5.3%	125	5.4%	0%
Beta blockers	919	31.7%	1363	34.0%	5%	742	32.1%	738	32.0%	0%
Bone Calcium regulators	50	1.7%	98	2.4%	5%	46	2.0%	51	2.2%	1%
Benzodiazepine	385	13.3%	569	14.2%	3%	312	13.5%	311	13.5%	0%
Bisphosphonate**s**	618	21.3%	716	17.9%	9%	494	21.4%	472	20.4%	2%
Beta agonist	298	10.3%	400	10.0%	1%	229	9.9%	242	10.5%	2%
H2 blockers	110	3.8%	165	4.1%	2%	94	4.1%	83	3.6%	3%
Channel calcium blockers	962	33.2%	1419	35.4%	5%	786	34.0%	775	33.6%	1%
Cholinesterase inhibitors	46	1.6%	54	1.3%	3%	34	1.5%	32	1.4%	1%
Glucocorticoid[i]	1800	62.1%	1998	49.8%	25%	1344	58.2%	1345	58.3%	0%
Loop diuretics	505	17.4%	714	17.8%	1%	396	17.2%	397	17.2%	0%
Nitrates	134	4.6%	208	5.2%	3%	112	4.9%	112	4.9%	0%
Fibrates	57	2.0%	85	2.1%	1%	43	1.9%	39	1.7%	2%

© 2023 Muanda FT et al. *JAMA Network Open.*

		Unmatched data (N=6909)					Matched data (N=4618)[b]				
		LD-MTX (n=2900)		HCQ (n=4009)		Standardized. Difference[c]	LD-MTX (n=2309)		HCQ (n=2309)		Standardized. Difference[c]
NSAIDs (excluding ASA)		634	21.9%	965	24.1%	5%	538	23.3%	552	23.9%	1%
Insulin		233	8.0%	340	8.5%	2%	177	7.7%	193	8.4%	3%
Opioids		673	23.2%	1002	25.0%	4%	565	24.5%	567	24.6%	0%
Over-active bladder medication		73	2.5%	149	3.7%	7%	61	2.6%	65	2.8%	1%
Potassium Sparing diuretics		177	6.1%	276	6.9%	3%	143	6.2%	142	6.1%	0%
Allopurinol		264	9.1%	450	11.2%	7%	218	9.4%	209	9.1%	1%
Anti-psychotics		66	2.3%	91	2.3%	0%	52	2.3%	49	2.1%	1%
Proton pump inhibitors		1259	43.4%	1863	46.5%	6%	1031	44.7%	1020	44.2%	1%
5 alpha-reductase		170	5.9%	228	5.7%	1%	137	5.9%	137	5.9%	0%
Selective serotonin reuptake inhibitors		325	11.2%	478	11.9%	2%	251	10.9%	253	11.0%	0%
Statins		1439	49.6%	2128	53.1%	7%	1142	49.5%	1157	50.1%	1%
Thiazide diuretics		569	19.6%	736	18.4%	3%	443	19.2%	452	19.6%	1%
Oral antidiabetics		642	22.1%	847	21.1%	2%	499	21.6%	496	21.5%	0%
Number of unique drug names	Mean ± SD	8.36	4.44	8.24	4.38	3%	8.23	4.37	8.23	4.45	0%
	Median (IQR)	8	(5-11)	8	(5-11)	.	8	(5-11)	8	(5-11)	.
	0-4	539	18.6%	776	19.4%	2%	450	19.5%	459	19.9%	1%
	5-9	1302	44.9%	1828	45.6%	1%	1021	44.2%	1036	44.9%	1%
	10-14	811	28.0%	1073	26.8%	3%	654	28.3%	620	26.9%	3%
	15-19	190	6.6%	263	6.6%	0%	149	6.5%	151	6.5%	0%
	20+	58	2.0%	69	1.7%	2%	35	1.5%	43	1.9%	3%
Number of unique dins	Mean ± SD	8.97	4.97	8.82	4.92	3%	8.84	4.9	8.82	5.01	0%

© 2023 Muanda FT et al. *JAMA Network Open.*

		Unmatched data (N=6909)					Matched data (N=4618)[b]				
		LD-MTX (n=2900)		HCQ (n=4009)		Standardized. Difference[c]	LD-MTX (n=2309)		HCQ (n=2309)		Standardized. Difference[c]
	Median (IQR)	8	(5-12)	8	(5-11)	.	8	(5-12)	8	(5-12)	.
	0-4	500	17.2%	725	18.1%	2%	418	18.1%	427	18.5%	1%
	5-9	1203	41.5%	1698	42.4%	2%	952	41.2%	970	42.0%	2%
	10-14	841	29.0%	1121	28.0%	2%	666	28.8%	640	27.7%	2%
	15-19	254	8.8%	326	8.1%	3%	203	8.8%	186	8.1%	3%
	20+	102	3.5%	139	3.5%	0%	70	3.0%	86	3.7%	4%
**Healthcare use[j]**											
Primary care visits	Mean ± SD	10.46	9.27	10.3	9.84	2%	10.52	9.48	10.43	10.44	1%
	Median (IQR)	8	(5-13)	8	(5-13)	.	8	(5-13)	8	(5-13)	.
	0-4	631	21.8%	903	22.5%	2%	508	22.0%	527	22.8%	2%
	5-9	1065	36.7%	1474	36.8%	0%	845	36.6%	828	35.9%	1%
	10-14	611	21.1%	844	21.1%	0%	475	20.6%	493	21.4%	2%
	15-19	274	9.4%	394	9.8%	1%	217	9.4%	218	9.4%	0%
	20+	319	11.0%	394	9.8%	4%	264	11.4%	243	10.5%	3%
Hospitalizations	Mean ± SD	0.2	0.58	0.16	0.54	7%	0.19	0.56	0.18	0.55	2%
	Median (IQR)	0	(0-0)	0	(0-0)	.	0	(0-0)	0	(0-0)	.
	0	2496	86.1%	3550	88.6%	8%	2002	86.7%	2020	87.5%	2%
	1	289	10.0%	331	8.3%	6%	224	9.7%	199	8.6%	4%
	2	71	2.4%	93	2.3%	1%	50	2.2%	67	2.9%	4%
	3+	44	1.5%	35	0.9%	6%	33	1.4%	23	1.0%	4%
Emergency department visits	Mean ± SD	0.77	1.4	0.7	1.4	5%	0.73	1.35	0.75	1.42	1%
	Median (IQR)	0	(0-1)	0	(0-1)	.	0	(0-1)	0	(0-1)	.

© 2023 Muanda FT et al. *JAMA Network Open.*

		Unmatched data (N=6909)					Matched data (N=4618)[b]				
		LD-MTX (n=2900)		HCQ (n=4009)		Standardized. Difference[c]	LD-MTX (n=2309)		HCQ (n=2309)		Standardized. Difference[c]
	0	1810	62.4%	2593	64.7%	5%	1465	63.4%	1447	62.7%	1%
	1	565	19.5%	782	19.5%	0%	442	19.1%	464	20.1%	3%
	2	268	9.2%	327	8.2%	4%	205	8.9%	206	8.9%	0%
	3+	257	8.9%	307	7.7%	4%	197	8.5%	192	8.3%	1%
TSH test		2001	69.0%	2998	74.8%	13%	1646	71.3%	1643	71.2%	0%
At-home physician service		72	2.5%	89	2.2%	2%	52	2.3%	49	2.1%	1%
Bone mineral density test		409	14.1%	657	16.4%	6%	341	14.8%	341	14.8%	0%
Cardiac catheterization		39	1.3%	48	1.2%	1%	30	1.3%	27	1.2%	1%
Cardiac stress test		397	13.7%	566	14.1%	1%	320	13.9%	330	14.3%	1%
Carotid ultrasound		156	5.4%	193	4.8%	3%	117	5.1%	120	5.2%	0%
Chest-xray		1387	47.8%	1694	42.3%	11%	1071	46.4%	1091	47.2%	2%
Cataract surgery		159	5.5%	205	5.1%	2%	123	5.3%	131	5.7%	2%
Cervical cancer screening		104	3.6%	130	3.2%	2%	77	3.3%	82	3.6%	2%
Colorectal cancer screening		441	15.2%	607	15.1%	0%	344	<15%	351	15.2%	1%
Cholesterol test (total cholesterol, HDL)		1889	65.1%	2663	66.4%	3%	1498	64.9%	1479	64.1%	2%
CT abdomen		337	11.6%	387	9.7%	6%	260	11.3%	264	11.4%	0%
CT extremities		31	1.1%	60	1.5%	4%	28	1.2%	30	1.3%	1%
CT head		284	9.8%	355	8.9%	3%	220	9.5%	216	9.4%	0%
CT neck		42	1.4%	42	1.0%	4%	26	1.1%	30	1.3%	2%
CT pelvis		316	10.9%	361	9.0%	6%	242	10.5%	250	10.8%	1%

© 2023 Muanda FT et al. *JAMA Network Open.*

		Unmatched data (N=6909)					Matched data (N=4618) [b]				
		LD-MTX (n=2900)		HCQ (n=4009)		Standardized. Difference[c]	LD-MTX (n=2309)		HCQ (n=2309)		Standardized. Difference[c]
CT spine		53	1.8%	104	2.6%	5%	48	2.1%	43	1.9%	1%
CT thorax		335	11.6%	464	11.6%	0%	267	11.6%	285	12.3%	2%
Echocardiography		716	24.7%	1046	26.1%	3%	575	24.9%	587	25.4%	1%
Flu shot		1439	49.6%	1951	48.7%	2%	1150	49.8%	1124	48.7%	2%
Cystoscopy		123	4.2%	172	4.3%	0%	97	4.2%	107	4.6%	2%
Hearing test		122	4.2%	210	5.2%	5%	107	4.6%	113	4.9%	1%
Mammography		323	11.1%	595	14.8%	11%	286	12.4%	284	12.3%	0%
Prostate-specific antigen (PSA) test		41	1.4%	36	0.9%	5%	27	1.2%	28	1.2%	0%
Holter monitoring		249	8.6%	361	9.0%	1%	206	8.9%	204	8.8%	0%
Parathyroid hormone testing		327	11.3%	686	17.1%	17%	292	12.6%	297	12.9%	1%
Pulmonary function test		366	12.6%	544	13.6%	3%	293	12.7%	303	13.1%	1%
Urinalysis		1662	57.3%	2489	62.1%	10%	1352	58.6%	1363	59.0%	1%
**Laboratory measurement**											
eGFR[k]	Mean ± SD	48.46	9.14	47.54	9.87	10%	48.22	9.27	48.12	9.58	1%
	Median (IQR)	51	(43-56)	50	(41-56)	.	50	(43-56)	50	(42-56)	.
Baseline eGFR categories	45-<60	2018	69.6%	2643	65.9%	8%	1581	68.5%	1587	68.7%	0%
	30-<45	746	25.7%	1100	27.4%	4%	612	26.5%	596	25.8%	2%
	<30	136	4.7%	266	6.6%	8%	116	5.0%	126	5.5%	2%
Urine ACR available		1073	37.0%	1597	39.8%	6%	860	37.2%	863	37.4%	0%
Baseline ACR categories, µg/mg	Missing	1827	63.0%	2412	60.2%	6%	1449	62.8%	1446	62.6%	0%
	<30	650	22.4%	994	24.8%	6%	518	22.4%	551	23.9 %	4%

© 2023 Muanda FT et al. *JAMA Network Open.*

		Unmatched data (N=6909)					Matched data (N=4618)[b]				
		LD-MTX (n=2900)		HCQ (n=4009)		Standardized. Difference[c]	LD-MTX (n=2309)		HCQ (n=2309)		Standardized. Difference[c]
	30-300	323	11.1%	433	10.8%	1%	258	11.2%	233	10.1%	4%
	>300	100	3.4%	170	4.2%	4%	84	3.6%	79	3.4%	1%

Abbreviations: LD-MTX, low-dose methotrexate; HCQ, hydroxychloroquine; ACE inhibitor, angiotensin-converting-enzyme inhibitor; H2 blockers, Histamine H2-receptor antagonists; eGFR, estimated glomerular filtration rate; IQR, interquartile range; LHIN, Local Health Integration Network; ACR, urine albumin-to-creatinine ratio.

[a] Unless otherwise specified in the footnotes, baseline characteristics were assessed on the date the patient filled a low-dose methotrexate prescription or a hydroxychloroquine prescription—the cohort entry date.

[b] Propensity score matching technique was used to balance comparison groups on indicators of baseline health, including all known indications for methotrexate use (including off-label indications). The propensity score was estimated using multivariable logistic regression with 140 covariates chosen *a priori* (defined in eTable 8 in the Supplement). We use greedy matching, to match low-dose methotrexate drug user (1:1) to hydroxychloroquine user based on the logit of the propensity score (within a caliper of ±0.2 standard deviations).[24.]

[c] The difference between the groups divided by the pooled SD; a value greater than 10% is interpreted as a meaningful difference.[25]

[d] Income was categorized into fifths of average neighborhood income on the cohort entry date.

[e] Baseline comorbidities were assessed in the 5-year period before the cohort entry date.

[f] Cancer includes the following types of cancer: skin, mouth (lip, tonsil, etc), throat, stomach, small/large intestine, liver, gall bladder, pancreas, breast, male/female reproductive organs, heart, lung, bone, urinary system (kidney, bladder, etc), endocrine glands, as well as leukemias and lymphomas

[g] Presence of kidney disease is a variable in the Charlson comorbidity index, which automatically results in all individuals receiving a minimum score of 2

[h] Medication use was examined in the 120-day period before the cohort entry date (the Ontario Drug Benefit program dispenses a maximum 100-day supply).

[l] Glucocorticoids included many medications regardless of their route of administration such as hydrocortisone acetate, dexamethasone, beclomethasone dipropionate, prednisone, hydrocortisone, flumetasone pivalate, clioquinol, betamethasone valerate, betamethasone, triamcinolone acetonide, triamcinolone diacetate, triamcinolone, flurandrenolide, betamethasone & dexamethasone sodium phosphate, cortisone acetate, dexamethasone tebutate, prednisolone, dexamethasone, corticotrophin, prednisolone acetate, fluocinolone acetonide, hydrocortisone sodium succinate, methylprednisolone sodium succinate, methylprednisolone acetate, methylprednisolone disodium phosphate, methylprednisolone, fluocinonide, betamethasone disodium phosphate, medrysone & polyvinyl alcohol, prednisolone acetate & sulfacetamide sodium, dexamethasone & neomycin sulfate & polymyxin b sulfate, clioquinol & flumetasone pivalate, clioquinol & hydrocortisone, 1,2-propanediol diacetate & acetic acid & benzethonium chloride & hydrocortisone, clioquinol & triamcinolone acetonide, flurandrenolide, fluocinolone acetonide, dexamethasone & neomycin sulfate, hydrocortisone & lidocaine HCL & neomycin sulfate, hemorrhoidal veinous plexus, prednisone & pheniramine maleate & inositol & phosphatidyl choline & vitamin a & vitamin d2& vitamin e, chloramphenicol & hydrocortisone acetate, haemorroidal veinous plexus, dexamethasone & framycetin sulfate & gramicidin, dibucaine hcl & esculin & framycetin sulfate & hydrocortisone, betamethasone valerate & neomycin sulfate, betamethasone valerate & gentamicin sulfate, prednisolone acetate & sulfacetamide sodium, ascorbic acid &

© 2023 Muanda FT et al. *JAMA Network Open.*

chlorpheniramine maleate & prednisone acetate, neomycin sulfate & prednisolone acetate & sulfacetamide sodium, gramicidin & neomycin sulfate & triamcinolone acetonide, methylprednisolone, acetylsalicylic acid & methyltestosterone, methylprednisolone sulfate & neomycin sulfate, hydrocortisone acetate & neomycin sulfate, aluminum chlorohydrate & methylprednisolone acetate & neomycin sulfate & sulfur, gramicidin & neomycin sulfate & nystatin & triamcinolone acetonide, hydrocortisone acetate & zinc oxide, hydrocortisone acetate & pramoxine hcl & zinc sulfate, aluminum chlorohydrate & methylprednisolone acetate & sulfur, hydrocortisone acetate & zinc oxide, hydrocortisone acetate & pramoxine hcl & zinc sulfate, desonide, clobetasol propionate, beclomethasone dipropionate & clioquinol, bacitracin zinc & hydrocortisone & neomycin sulfate & polymyxin b sulfate, hydrocortisone & neomycin sulfate & polymyxin b sulfate, gramicidin & neomycin sulfate & nystatin & triamcinolone acetonide, fluorometholone & polyvinyl alcohol, aluminum chlorohydrate & methylprednisolone acetate & sulfur, fluorometholone, lidocaine hcl & methylprednisolone acetate, flumetasone pivalate & salicylic acid, fluorometholone, lidocaine hcl & methylprednisolone acetate, aclometasone dipropionate, allantoin & chloramphenicol & hydrocortisone, amcinonide, atropine sulfate & prednisolone acetate, bacitracin & hydrocortisone & neomycin sulfate & polymyxin b sulfate, benzalkonium & dexamethasone & tobramycin , benzocaine & hydrocortisone acetate & zinc sulfate, betamethasone & sulfacetamide sodium, betamethasone acetate & betamethasone sodium phosphate, betamethasone benzoate, betamethasone dipropionate, betamethasone dipropionate &calcipotriene, betamethasone dipropionate &clotrimazole, betamethasone dipropionate & gentamicin sulfate, betamethasone dipropionate & salicylic acid, betamethasone disodium phosphate, betamethasone valerate & salicylic acid, betamethasone valerate & gentamycin sulfate, betamethasone valerate & neomycin sulfate, budesonide, camphor & hydrocortisone & menthol, chlorbutol & dexamethasone &tobramycin, ciclesonide.

[j] Total number of healthcare visits/tests in the 12-month period before the cohort entry date.

[k] The most recent eGFR measurement in the 365-day period before the cohort entry date (including the cohort entry date); eGFR was calculated using the new Chronic Kidney Disease (CKD)–Epidemiology (EPI) equation: 142× min([serum creatinine concentration in µmol/L/88.4]/κ, 1)$^{\alpha}$ × max([serum creatinine concentration in µmol/L/88.4]/κ, 1)$^{-1.200}$× 0.9938^{Age} × 1.012 [if female]; κ=0.7 if female and 0.9 if male; α=-0.241 if female and -0.302 if male; min=the minimum of serum creatinine concentration/κ or 1; max=the maximum of serum creatinine concentration/κ or 1.

© 2023 Muanda FT et al. *JAMA Network Open.*

eTable 9. Dose and duration of continuous study DMARD dispensing in older adults with chronic kidney disease newly prescribed LD-MTX vs. HCQ in Ontario, Canada (2008-2021)

**Dose and duration of continuous DMARD dispensing**	**LD-MTX**	**HCQ**
	**n=2900 (42.0%)**	**n=4009 (58.0%)**
Median weekly dose, mg (IQR)	15 (10 to 17.5)	NA
Median daily dose, mg (IQR)	NA	400 (200 to 400)
Median duration,[a] days (IQR)	145 (59-424)	124 (30-438)

Abbreviations: DMARD, disease-modifying antirheumatic drug; LD-MTX, low-dose methotrexate; HCQ, hydroxychloroquine; IQR, interquartile range; NA, not applicable.

[a]Defined as consecutive prescription claims within a period equivalent to 150% of the days supplied for the previous prescription.

© 2023 Muanda FT et al. *JAMA Network Open.*

eTable 10. Characteristics of 41 patients with a hospital admission with serious adverse events after starting a study DMARD in the matched cohort

Characteristics	Composite outcome (n=41)
Median [IQR] length of the hospital stays	9 (5-16) days
Intensive care unit	15 (37%)
Mechanical ventilation	11 (27%)
Acute dialysis	<6 (<15%)
CT head	<6 (<15%)

© 2023 Muanda FT et al. *JAMA Network Open.*

eTable 11. Frequency of each component in the matched cohort

Event type	Aggregate event rates	
Pneumotoxicity	82	1.78%
Sepsis	32	0.69%
Myelotoxicity	27	0.58%
Hepatotoxicity	<6	<0.13%

© 2023 Muanda FT et al. *JAMA Network Open.*

eTable 12. Baseline characteristics[a] of older adults with an eGFR>60 mL/min/1.73m^2 newly prescribed low-dose methotrexate (LD MTX) vs those newly prescribed hydroxychloroquine (HCQ) in Ontario, Canada (2008–2021)

		Unmatched data (N=29335)					Matched data (N=20554)[b]				
		LD-MTX (n=13 127)		HCQ (n=16 208)		Standardized Difference[c]	LD-MTX (n=10 277)		HCQ (n=10 277)		Standardized Difference[c]
**Demographics**											
Age at cohort entry	Mean ± SD	72.8	5.9	72.9	5.9	1%	72.9	5.9	73.0	6.0	1%
	Median (IQR)	71	(68-77)	72	(68-77)	.	72	(68-77)	72	(68-77)	.
	66-<70	4954	37.7%	6010	37.1%	1%	3755	36.5%	3778	36.8%	1%
	70-<75	3750	28.6%	4655	28.7%	0%	2974	28.9%	2933	28.5%	1%
	75-<80	2447	18.6%	3077	19.0%	1%	1975	19.2%	1969	19.2%	0%
	80-<85	1400	10.7%	1688	10.4%	1%	1125	10.9%	1095	10.7%	1%
	85-<90	468	3.6%	640	3.9%	2%	365	3.6%	408	4.0%	2%
	90+	108	0.8%	138	0.9%	1%	83	0.8%	94	0.9%	1%
sex	F	7995	60.9%	11416	70.4%	20%	6680	65.0%	6716	65.3%	1%
	M	5132	39.1%	4792	29.6%	20%	3597	35.0%	3561	34.7%	1%
Year of cohort entry	2008	366	2.8%	305	1.9%	6%	251	2.4%	255	2.5%	1%
	2009	691	5.3%	697	4.3%	5%	511	5.0%	506	4.9%	0%
	2010	927	7.1%	935	5.8%	5%	708	6.9%	683	6.6%	1%
	2011	961	7.3%	1125	6.9%	2%	780	7.6%	775	7.5%	0%
	2012	1060	8.1%	1200	7.4%	3%	814	7.9%	810	7.9%	0%
	2013	1036	7.9%	1228	7.6%	1%	804	7.8%	821	8.0%	1%
	2014	1000	7.6%	1303	8.0%	1%	772	7.5%	788	7.7%	1%
	2015	1037	7.9%	1371	8.5%	2%	818	8.0%	812	7.9%	0%
	2016	1068	8.1%	1405	8.7%	2%	851	8.3%	858	8.3%	0%

© 2023 Muanda FT et al. *JAMA Network Open.*

		Unmatched data (N=29335)					Matched data (N=20554) [b]				
		LD-MTX (n=13 127)		HCQ (n=16 208)		Standardized Difference[c]	LD-MTX (n=10 277)		HCQ (n=10 277)		Standardized Difference[c]
	2017	1068	8.1%	1333	8.2%	0%	833	8.1%	838	8.2%	0%
	2018	1058	8.1%	1415	8.7%	2%	845	8.2%	863	8.4%	1%
	2019	1188	9.1%	1541	9.5%	1%	924	9.0%	899	8.7%	1%
	2020	1053	8.0%	1534	9.5%	5%	878	8.5%	884	8.6%	0%
	2021	614	4.7%	816	5.0%	1%	488	4.7%	485	4.7%	0%
Location	Urban	11504	87.6%	14178	87.5%	0%	8994	87.5%	8994	87.5%	0%
	Rural	1623	12.4%	2030	12.5%	0%	1283	12.5%	1283	12.5%	0%
Residence	1	80	0.6%	65	0.4%	3%	60	0.6%	52	0.5%	1%
LHIN	1	597	4.5%	540	3.3%	6%	421	4.1%	412	4.0%	1%
	2	877	6.7%	1200	7.4%	3%	730	7.1%	747	7.3%	1%
	3	849	6.5%	750	4.6%	8%	584	5.7%	560	5.4%	1%
	4	1983	15.1%	2969	18.3%	9%	1675	16.3%	1672	16.3%	0%
	5	927	7.1%	938	5.8%	5%	714	6.9%	703	6.8%	0%
	6	1030	7.8%	1173	7.2%	2%	822	8.0%	819	8.0%	0%
	7	842	6.4%	920	5.7%	3%	653	6.4%	634	6.2%	1%
	8	1241	9.5%	2009	12.4%	9%	1022	9.9%	1025	10.0%	0%
	9	1392	10.6%	1618	10.0%	2%	1056	10.3%	1056	10.3%	0%
	10	819	6.2%	709	4.4%	8%	573	5.6%	561	5.5%	0%
	11	1338	10.2%	1648	10.2%	0%	1040	10.1%	1082	10.5%	1%
	12	412	3.1%	815	5.0%	10%	372	3.6%	399	3.9%	2%
	13	623	4.7%	666	4.1%	3%	448	4.4%	449	4.4%	0%
	14	197	1.5%	253	1.6%	1%	167	1.6%	158	1.5%	1%

© 2023 Muanda FT et al. *JAMA Network Open.*

		Unmatched data (N=29335)					Matched data (N=20554)[b]				
		LD-MTX (n=13 127)		HCQ (n=16 208)		Standardized Difference[c]	LD-MTX (n=10 277)		HCQ (n=10 277)		Standardized Difference[c]
Socio-economic status[d]	1	2348	17.9%	2721	16.8%	3%	1797	17.5%	1752	17.0%	1%
	2	2691	20.5%	3219	19.9%	1%	2117	20.6%	2111	20.5%	0%
	3	2659	20.3%	3298	20.3%	0%	2097	20.4%	2114	20.6%	0%
	4	2581	19.7%	3341	20.6%	2%	2019	19.6%	2024	19.7%	0%
	5	2848	21.7%	3629	22.4%	2%	2247	21.9%	2276	22.1%	0%
**Prescriber**											
Rheumatologist		7410	56.4%	10292	63.5%	15%	6450	62.8%	6388	62.2%	1%
General practitioner		1580	12.0%	2179	13.4%	4%	1284	12.5%	1286	12.5%	0%
Internist		899	6.8%	1056	6.5%	1%	719	7.0%	735	7.2%	1%
Dermatologist		1428	10.9%	632	3.9%	27%	473	4.6%	521	5.1%	2%
Other		654	5.0%	685	4.2%	4%	446	4.3%	434	4.2%	0%
Missing		1156	8.8%	1364	8.4%	1%	905	8.8%	913	8.9%	0%
**Comorbidities[e]**											
Acute kidney injury		121	0.9%	152	0.9%	0%	96	0.9%	84	0.8%	1%
Alcoholism		124	0.9%	184	1.1%	2%	104	1.0%	97	0.9%	1%
Angina		1454	11.1%	1885	11.6%	2%	1150	11.2%	1179	11.5%	1%
Atrial fibrillation/flutter		437	3.3%	495	3.1%	1%	340	3.3%	339	3.3%	0%
Bipolar disorder		197	1.5%	285	1.8%	2%	154	1.5%	147	1.4%	1%
Chronic liver disease		540	4.1%	836	5.2%	5%	423	4.1%	435	4.2%	1%
Chronic obstructive pulmonary disease		3230	24.6%	4366	26.9%	5%	2559	24.9%	2594	25.2%	1%
Cirrhosis		379	2.9%	581	3.6%	4%	299	2.9%	301	2.9%	0%

© 2023 Muanda FT et al. *JAMA Network Open.*

	Unmatched data (N=29335)					Matched data (N=20554) [b]				
	LD-MTX (n=13 127)		HCQ (n=16 208)		Standardized Difference[c]	LD-MTX (n=10 277)		HCQ (n=10 277)		Standardized Difference[c]
Coronary artery disease (minus angina)	2434	18.5%	2997	18.5%	0%	1873	18.2%	1882	18.3%	0%
Dementia	670	5.1%	891	5.5%	2%	527	5.1%	505	4.9%	1%
Anemia	2381	18.1%	2874	17.7%	1%	1860	18.1%	1835	17.9%	1%
Glaucoma	1372	10.5%	1847	11.4%	3%	1089	10.6%	1073	10.4%	1%
Major hemorrhage	626	4.8%	889	5.5%	3%	500	4.9%	508	4.9%	0%
Congestive heart failure	864	6.6%	988	6.1%	2%	670	6.5%	692	6.7%	1%
Hypertension	7655	58.3%	9644	59.5%	2%	6007	58.5%	6011	58.5%	0%
Hypokalemia	132	1.0%	158	1.0%	0%	103	1.0%	100	1.0%	0%
Hyponatremia	133	1.0%	173	1.1%	1%	106	1.0%	102	1.0%	0%
Hypothyroidism	1551	11.8%	2301	14.2%	7%	1288	12.5%	1275	12.4%	0%
Migraine	476	3.6%	703	4.3%	4%	394	3.8%	392	3.8%	0%
Obesity	723	5.5%	1020	6.3%	3%	569	5.5%	580	5.6%	0%
Parkinson disease	131	1.0%	160	1.0%	0%	105	1.0%	98	1.0%	0%
Peripheral vascular disease	98	0.7%	111	0.7%	0%	78	0.8%	72	0.7%	1%
Schizophrenia	185	1.4%	164	1.0%	4%	117	1.1%	115	1.1%	0%
Ischaemic stroke	105	0.8%	92	0.6%	2%	75	0.7%	68	0.7%	0%
Depression	972	7.4%	1303	8.0%	2%	779	7.6%	760	7.4%	1%
Rheumatoid arthritis	7083	54.0%	8055	49.7%	9%	17	0.2%	14	0.1%	3%
Syncope	112	0.9%	160	1.0%	1%	5938	57.8%	5915	57.6%	0%
Arrhythmia	645	4.9%	734	4.5%	2%	95	0.9%	93	0.9%	0%
Inflammatory bowel disease	291	2.2%	127	0.8%	12%	494	4.8%	493	4.8%	0%

© 2023 Muanda FT et al. *JAMA Network Open.*

	Unmatched data (N=29335)					Matched data (N=20554) [b]				
	LD-MTX (n=13 127)		HCQ (n=16 208)		Standardized Difference[c]	LD-MTX (n=10 277)		HCQ (n=10 277)		Standardized Difference[c]
Major cancer	4578	34.9%	5870	36.2%	3%	127	1.2%	121	1.2%	0%
Prostatic hyperplasia	1458	11.1%	1486	9.2%	6%	3584	34.9%	3588	34.9%	0%
Fracture	804	6.1%	1067	6.6%	2%	1074	10.5%	1046	10.2%	1%
Falls	336	2.6%	426	2.6%	0%	649	6.3%	645	6.3%	0%
Hyperkaliemia	14	0.1%	27	0.2%	3%	267	2.6%	257	2.5%	1%
Prostatitis	332	2.5%	334	2.1%	3%	13	0.1%	13	0.1%	0%
Hypotension	108	0.8%	160	1.0%	2%	230	2.2%	215	2.1%	1%
Gallstones /biliary stones	521	4.0%	734	4.5%	2%	86	0.8%	72	0.7%	1%
Prior pneumotoxcity	295	2.2%	338	2.1%	1%	420	4.1%	426	4.1%	0%
Prior myelotoxicity	69	0.5%	98	0.6%	1%	240	2.3%	220	2.1%	1%
Prior sepsis	80	0.6%	106	0.7%	1%	54	0.5%	54	0.5%	0%
Prior methotrexate toxicity	401	3.1%	498	3.1%	0%	71	0.7%	61	0.6%	1%
Psoriasis	2347	17.9%	595	3.7%	47%	327	3.2%	305	3.0%	1%
Dermatomyositis	855	6.5%	2287	14.1%	25%	563	5.5%	586	5.7%	1%
Sarcoidosis	106	0.8%	67	0.4%	5%	814	7.9%	802	7.8%	0%
Systemic sclerosis or scleroderma	962	7.3%	1149	7.1%	1%	68	0.7%	60	0.6%	1%
Lymphoma	30	0.2%	32	0.2%	0%	724	7.0%	712	6.9%	0%
Systemic lupus erythematosus	1215	9.3%	2809	17.3%	24%	1086	10.6%	1096	10.7%	0%
Atopic dermatitis or eczema	3941	30.0%	4633	28.6%	3%	2851	27.7%	2806	27.3%	1%
Hypoglycemia	21	0.2%	21	0.1%	3%	14	0.1%	15	0.1%	0%

© 2023 Muanda FT et al. *JAMA Network Open.*

		Unmatched data (N=29335)					Matched data (N=20554)[b]				
		LD-MTX (n=13 127)		HCQ (n=16 208)		Standardized Difference[c]	LD-MTX (n=10 277)		HCQ (n=10 277)		Standardized Difference[c]
Coeliac disease		56	0.4%	70	0.4%	0%	40	0.4%	42	0.4%	0%
Ulcerative colitis (UC)		276	2.1%	216	1.3%	6%	182	1.8%	165	1.6%	2%
Crohn disease		287	2.2%	135	0.8%	12%	118	1.1%	120	1.2%	1%
Acute urinary retention		148	1.1%	174	1.1%	0%	112	1.1%	111	1.1%	0%
Myocardial infarction		234	1.8%	249	1.5%	2%	176	1.7%	183	1.8%	1%
Dyslipidemia		3496	26.6%	4444	27.4%	2%	2729	26.6%	2741	26.7%	0%
Macula degeneration		390	3.0%	312	1.9%	7%	250	2.4%	244	2.4%	0%
Gastroesophageal reflux disease		2866	21.8%	4114	25.4%	8%	2329	22.7%	2328	22.7%	0%
Osteoarthritis		1133	8.6%	1642	10.1%	5%	942	9.2%	937	9.1%	0%
Major surgery		522	4.0%	638	3.9%	1%	398	3.9%	403	3.9%	0%
Prostate cancer		479	3.6%	519	3.2%	2%	346	3.4%	377	3.7%	2%
Diabetes		2174	16.6%	2397	14.8%	5%	1619	15.8%	1635	15.9%	0%
Urinary tract infection		405	3.1%	539	3.3%	1%	321	3.1%	322	3.1%	0%
Gout		962	7.3%	1264	7.8%	2%	760	7.4%	763	7.4%	0%
Charlson comorbidity index[g]	Mean ± SD	0.22	0.77	0.19	0.69	4%	0.21	0.75	0.21	72.0%	0%
	Median (IQR)	0	(0-0)	0	(0-0)	.	0	(0-0)	0	(0-0)	.
	0	11683	89.0%	14540	89.7%	2%	9170	89.2%	9153	89.1%	0%
	1	719	5.5%	860	5.3%	1%	563	5.5%	566	5.5%	0%
	2	393	3.0%	482	3.0%	0%	293	2.9%	324	3.2%	2%
	3+	332	2.5%	326	2.0%	3%	251	2.4%	234	2.3%	1%
Medication use[h]											

© 2023 Muanda FT et al. *JAMA Network Open.*

	Unmatched data (N=29335)					Matched data (N=20554)[b]				
	LD-MTX (n=13 127)		HCQ (n=16 208)		Standardized Difference[c]	LD-MTX (n=10 277)		HCQ (n=10 277)		Standardized Difference[c]
Alpha-adrenergic blocking agents	176	1.3%	224	1.4%	1%	140	1.4%	145	1.4%	0%
Anti-arrhythmic	34	0.3%	32	0.2%	2%	26	0.3%	26	0.3%	0%
Antibiotics	3181	24.2%	4101	25.3%	3%	2465	24.0%	2449	23.8%	0%
Ace inhibitor	3196	24.3%	3643	22.5%	4%	2400	23.4%	2432	23.7%	1%
Anticoagulants	866	6.6%	998	6.2%	2%	663	6.5%	654	6.4%	0%
Anticonvulsants	770	5.9%	1204	7.4%	6%	634	6.2%	657	6.4%	1%
Angiotensin II receptor blockers	2549	19.4%	3567	22.0%	6%	2076	20.2%	2060	20.0%	0%
Aromatase inhibitors	73	0.6%	111	0.7%	1%	58	0.6%	69	0.7%	1%
Aspirin	192	1.5%	177	1.1%	4%	132	1.3%	130	1.3%	0%
Antiplatelet agents	467	3.6%	537	3.3%	2%	355	3.5%	365	3.6%	1%
Antifungals	158	1.2%	227	1.4%	2%	119	1.2%	122	1.2%	0%
Tricyclic antidepressant	1376	10.5%	2027	12.5%	6%	1092	10.6%	1143	11.1%	2%
Gastrointestinal drugs	651	5.0%	659	4.1%	4%	481	4.7%	469	4.6%	0%
Beta-blockers	2423	18.5%	2979	18.4%	0%	1887	18.4%	1909	18.6%	1%
Bone Calcium regulators	195	1.5%	335	2.1%	5%	169	1.6%	172	1.7%	1%
Benzodiazepine	1384	10.5%	1896	11.7%	4%	1098	10.7%	1073	10.4%	1%
Bisphosphonates	2506	19.1%	2705	16.7%	6%	1965	19.1%	1971	19.2%	0%
Beta agonist	971	7.4%	1337	8.2%	3%	759	7.4%	753	7.3%	0%
H2 blockers	455	3.5%	571	3.5%	0%	368	3.6%	374	3.6%	0%
Channel calcium blockers	2841	21.6%	3645	22.5%	2%	2223	21.6%	2210	21.5%	0%
Cholinesterase inhibitors	147	1.1%	142	0.9%	2%	101	1.0%	99	1.0%	0%

© 2023 Muanda FT et al. *JAMA Network Open.*

		Unmatched data (N=29335)					Matched data (N=20554)[b]				
		LD-MTX (n=13 127)		HCQ (n=16 208)		Standardized Difference[c]	LD-MTX (n=10 277)		HCQ (n=10 277)		Standardized Difference[c]
Glucocorticoid[i]		7566	57.6%	7569	46.7%	22%	5507	53.6%	5490	53.4%	0%
Loop diuretics		777	5.9%	836	5.2%	3%	579	5.6%	572	5.6%	0%
Nitrates		278	2.1%	346	2.1%	0%	216	2.1%	218	2.1%	0%
Fibrates		95	0.7%	125	0.8%	1%	71	0.7%	80	0.8%	1%
Nsaids (excluding ASA)		3890	29.6%	5086	31.4%	4%	3240	31.5%	3254	31.7%	0%
Insulin		423	3.2%	511	3.2%	0%	319	3.1%	329	3.2%	1%
Opioids		2695	20.5%	3245	20.0%	1%	2129	20.7%	2134	20.8%	0%
Over-active bladder medication		249	1.9%	398	2.5%	4%	203	2.0%	217	2.1%	1%
Potassium Sparing diuretics		320	2.4%	409	2.5%	1%	256	2.5%	257	2.5%	0%
Allopurinol		423	3.2%	540	3.3%	1%	336	3.3%	349	3.4%	1%
Anti-psychotics		210	1.6%	249	1.5%	1%	156	1.5%	158	1.5%	0%
Proton pump inhibitors		4317	32.9%	5669	35.0%	4%	3496	34.0%	3490	34.0%	0%
5 alpha-reductase		848	6.5%	842	5.2%	6%	632	6.1%	621	6.0%	0%
Selective serotonin reuptake inhibitors		1251	9.5%	1734	10.7%	4%	982	9.6%	989	9.6%	0%
Statins		5049	38.5%	6583	40.6%	4%	3926	38.2%	3959	38.5%	1%
Thiazide diuretics		1705	13.0%	2106	13.0%	0%	1324	12.9%	1344	13.1%	1%
Oral antidiabetics		2044	15.6%	2247	13.9%	5%	1519	14.8%	1538	15.0%	1%
Number of unique drug names	Mean ± SD	6.36	4.14	6.13	3.96	6%	6.2	4.04	6.21	4.02	0%
	Median (IQR)	6	(3-9)	6	(3-8)	.	6	(3-8)	6	(3-8)	.
	0-4	4758	36.2%	6273	38.7%	5%	3852	37.5%	3939	38.3%	2%
	5-9	5767	43.9%	7019	43.3%	1%	4503	43.8%	4419	43.0%	2%

© 2023 Muanda FT et al. *JAMA Network Open.*

		Unmatched data (N=29335)					Matched data (N=20554)[b]				
		LD-MTX (n=13 127)		HCQ (n=16 208)		Standardized Difference[c]	LD-MTX (n=10 277)		HCQ (n=10 277)		Standardized Difference[c]
	10-14	2059	15.7%	2324	14.3%	4%	1542	15.0%	1515	14.7%	1%
	15-19	440	3.4%	501	3.1%	2%	317	3.1%	340	3.3%	1%
	20+	103	0.8%	91	0.6%	2%	63	0.6%	64	0.6%	0%
Number of unique dins	Mean ± SD	6.79	4.58	6.51	434.0%	6%	6.61	4.44	6.61	4.43	0%
	Median (IQR)	6	(3-9)	6	(3-9)	.	6	(3-9)	6	(3-9)	.
	0-4	4510	34.4%	5924	36.5%	4%	3662	35.6%	3713	36.1%	1%
	5-9	5538	42.2%	6851	42.3%	0%	4329	42.1%	4303	41.9%	0%
	10-14	2285	17.4%	2583	15.9%	4%	1729	16.8%	1673	16.3%	1%
	15-19	580	4.4%	674	4.2%	1%	417	4.1%	464	4.5%	2%
	20+	214	1.6%	176	1.1%	4%	140	1.4%	124	1.2%	2%
Healthcare use[j]											
Primary care visits	Mean ± SD	9.05	7.98	8.82	7.13	3%	8.91	7.63	8.89	7.37	0%
	Median (IQR)	7	(4-12)	7	(4-11)	.	7	(4-12)	7	(4-11)	.
	0-4	3685	28.1%	4254	26.2%	4%	2901	28.2%	2723	26.5%	4%
	5-9	4899	37.3%	6422	39.6%	5%	3865	37.6%	4023	39.1%	3%
	10-14	2476	18.9%	3229	19.9%	3%	1956	19.0%	2039	19.8%	2%
	15-19	1086	8.3%	1251	7.7%	2%	826	8.0%	799	7.8%	1%
	20+	981	7.5%	1052	6.5%	4%	729	7.1%	693	6.7%	2%
Hospitalizations	Mean ± SD	0.12	0.45	0.1	0.41	5%	0.12	0.44	0.11	43.0%	2%
	Median (IQR)	0	(0-0)	0	(0-0)	.	0	(0-0)	0	(0-0)	.
	0	11957	91.1%	14968	92.3%	4%	9406	91.5%	9412	91.6%	0%
	1	897	6.8%	963	5.9%	4%	670	6.5%	670	6.5%	0%

© 2023 Muanda FT et al. *JAMA Network Open.*

		Unmatched data (N=29335)					Matched data (N=20554) [b]				
		LD-MTX (n=13 127)		HCQ (n=16 208)		Standardized Difference[c]	LD-MTX (n=10 277)		HCQ (n=10 277)		Standardized Difference[c]
	2	172	1.3%	191	1.2%	1%	126	1.2%	128	1.2%	0%
	3+	101	0.8%	86	0.5%	4%	75	0.7%	67	0.7%	0%
Emergency departments visits	Mean ± SD	0.57	1.16	0.54	1.15	3%	0.57	1.17	0.55	1.15	2%
	Median (IQR)	0	(0-1)	0	(0-1)	.	0	(0-1)	0	(0-1)	.
	0	9048	68.9%	11400	70.3%	3%	7103	69.1%	7128	69.4%	1%
	1	2381	18.1%	2863	17.7%	1%	1849	18.0%	1883	18.3%	1%
	2	903	6.9%	1034	6.4%	2%	696	6.8%	669	6.5%	1%
	3+	795	6.1%	911	5.6%	2%	629	6.1%	597	5.8%	1%
TSH test		9073	69.1%	12277	75.7%	15%	7362	71.6%	7398	72.0%	1%
At-home physician service		214	1.6%	220	1.4%	2%	159	1.5%	155	1.5%	0%
Bone mineral density test		2218	16.9%	2885	17.8%	2%	1820	17.7%	1836	17.9%	1%
Cardiac catheterization		137	1.0%	176	1.1%	1%	104	1.0%	110	1.1%	1%
Cardiac stress test		8921	68.0%	11755	72.5%	10%	1311	12.8%	1310	12.7%	0%
Carotid ultrasound		1651	12.6%	2123	13.1%	1%	395	3.8%	400	3.9%	1%
Chest-x-ray		503	3.8%	655	4.0%	1%	4163	40.5%	4176	40.6%	0%
Cataract surgery		5514	42.0%	6103	37.7%	9%	500	4.9%	483	4.7%	1%
Cervical cancer screening		643	4.9%	751	4.6%	1%	657	6.4%	655	6.4%	0%
Colorectal cancer screening		759	5.8%	1189	7.3%	6%	1951	19.0%	1916	18.6%	1%
Cholesterol test (total cholesterol, HDL)		2454	18.7%	3094	19.1%	1%	6950	67.6%	6982	67.9%	1%
CT abdomen		8871	67.6%	11269	69.5%	4%	925	9.0%	909	8.8%	1%

© 2023 Muanda FT et al. *JAMA Network Open.*

		Unmatched data (N=29335)					Matched data (N=20554)[b]				
		LD-MTX (n=13 127)		HCQ (n=16 208)		Standardized Difference[c]	LD-MTX (n=10 277)		HCQ (n=10 277)		Standardized Difference[c]
CT extremities		1242	9.5%	1400	8.6%	3%	120	1.2%	112	1.1%	1%
CT head		140	1.1%	175	1.1%	0%	744	7.2%	740	7.2%	0%
CT neck		971	7.4%	1082	6.7%	3%	147	1.4%	150	1.5%	1%
CT pelvis		203	1.5%	208	1.3%	2%	865	8.4%	853	8.3%	0%
CT spine		1176	9.0%	1291	8.0%	4%	197	1.9%	189	1.8%	1%
CT thorax		250	1.9%	284	1.8%	1%	960	9.3%	956	9.3%	0%
Echocardiography		1256	9.6%	1527	9.4%	1%	1823	17.7%	1817	17.7%	0%
Flu shot		2262	17.2%	2998	18.5%	3%	4434	43.1%	4435	43.2%	0%
Cystoscopy		5564	42.4%	7231	44.6%	4%	336	3.3%	341	3.3%	0%
Hearing test		430	3.3%	542	3.3%	0%	482	4.7%	497	4.8%	0%
Mammography		603	4.6%	852	5.3%	3%	1801	17.5%	1796	17.5%	0%
Prostate-specific antigen (PSA) test		2120	16.1%	3400	21.0%	13%	211	2.1%	211	2.1%	0%
Holter monitoring		774	5.9%	1093	6.7%	3%	622	6.1%	632	6.1%	0%
Parathyroid hormone testing		695	5.3%	1324	8.2%	12%	622	6.1%	620	6.0%	0%
Pulmonary function test		1314	10.0%	1918	11.8%	6%	1076	10.5%	1066	10.4%	0%
Urinalysis		7180	54.7%	9483	58.5%	8%	5746	55.9%	5737	55.8%	0%
Laboratory measurement											
eGFR[k]	Mean ± SD	83.42	11.67	82.81	11.56	5%	83.36	11.69	83.32	11.49	0%
	Median (IQR)	85	(74-93)	84	(73-93)	.	85	(74-93)	85	(74-93)	.
Urine ACR available		3395	25.9%	4430	27.3%	3%	2689	26.2%	2710	26.4%	0%
	Missing	9732	74.1%	11778	72.7%	3%	7588	73.8%	7567	73.6%	0%

© 2023 Muanda FT et al. *JAMA Network Open.*

		Unmatched data (N=29335)					Matched data (N=20554)[b]				
		LD-MTX (n=13 127)		HCQ (n=16 208)		Standardized Difference[c]	LD-MTX (n=10 277)		HCQ (n=10 277)		Standardized Difference[c]
Baseline ACR categories, µg/mg											
	<3	2726	20.8%	3559	22.0%	3%	2178	21.2%	2155	21.0%	0%
	3-30	588	4.5%	751	4.6%	0%	455	4.4%	481	4.7%	1%
	>30	81	0.6%	120	0.7%	1%	56	0.5%	74	0.7%	3%

Abbreviations: LD-MTX, low-dose methotrexate; HCQ, hydroxychloroquine; ACE inhibitor, angiotensin-converting-enzyme inhibitor; H2 blockers, Histamine H2-receptor antagonists eGFR, estimated glomerular filtration rate; IQR, interquartile range; LHIN, Local Health Integration Network; ACR, urine albumin-to-creatinine ratio.

[a] Unless otherwise specified in the footnotes, baseline characteristics were assessed on the date the patient filled a low-dose methotrexate prescription or a hydroxychloroquine prescription—the cohort entry date.

[b] Propensity score matching technique was used to balance comparison groups on indicators of baseline health, including all known indications for methotrexate use (including off-label indications). The propensity score was estimated using multivariable logistic regression with 140 covariates chosen *a priori* (defined in eTable 8 in the Supplement). We use greedy matching to match low-dose methotrexate drug user (1:1) to hydroxychloroquine user based on the logit of the propensity score (within a caliper of ±0.2 standard deviations).[24]

[c] The difference between the groups divided by the pooled SD; a value greater than 10% is interpreted as a meaningful difference.[25]

[d] Income was categorized into fifths of average neighborhood income on the cohort entry date.

[e] Baseline comorbidities were assessed in the 5-year period before the cohort entry date.

[f] Cancer includes the following types of cancer: skin, mouth (lip, tonsil, etc), throat, stomach, small/large intestine, liver, gall bladder, pancreas, breast, male/female reproductive organs, heart, lung, bone, urinary system (kidney, bladder, etc), endocrine glands, as well as leukemias and lymphomas

[g] Charlson comorbidity index was calculated based on hospitalization data during the 5 years preceding the index date. For each patient, the index considers hospitalizations with the comorbidities of interest (acute myocardial infarction, congestive heart failure, peripheral vascular disease, cerebrovascular disease, dementia, chronic lung disease, rheumatic disease, peptic ulcer disease, mild and moderate/severe liver disease, diabetes mellitus with and without complications, hemiplegia/paraplegia, renal disease, cancer and metastatic solid tumour, and AIDS/HIV). It assigns a point score (1, 2, 3, or 6) for each comorbidity and sums them to generate an overall score of disease burden. The final risk scores range between 0 and 13, with higher values associated with higher mortality. Patients without a history of hospitalization received a score of 0.

[h] Medication use was examined in the 120-day period before the cohort entry date (the Ontario Drug Benefit program dispenses a maximum 100-day supply).

[l] Glucocorticoids included many medications regardless of their route of administration such as hydrocortisone acetate, dexamethasone, beclomethasone dipropionate, prednisone, hydrocortisone, flumetasone pivalate, clioquinol, betamethasone valerate, betamethasone, triamcinolone acetonide, triamcinolone diacetate, triamcinolone, flurandrenolide, betamethasone & dexamethasone sodium phosphate, cortisone acetate, dexamethasone tebutate, prednisolone,

© 2023 Muanda FT et al. *JAMA Network Open.*

dexamethasone, corticotrophin, prednisolone acetate, fluocinolone acetonide, hydrocortisone sodium succinate, methylprednisolone sodium succinate, methylprednisolone acetate, methylprednisolone disodium phosphate, methylprednisolone, fluocinonide, betamethasone disodium phosphate, medrysone & polyvinyl alcohol, prednisolone acetate & sulfacetamide sodium, dexamethasone & neomycin sulfate & polymyxin b sulfate, clioquinol & flumetasone pivalate, clioquinol & hydrocortisone, 1,2-propanediol diacetate & acetic acid & benzethonium chloride & hydrocortisone, clioquinol & triamcinolone acetonide, flurandrenolide, fluocinolone acetonide, dexamethasone & neomycin sulfate, hydrocortisone & lidocaine hcl & neomycin sulfate, haemorroidal veinous plexus, prednisone & pheniramine maleate & inositol & phosphatidyl choline & vitamin a & vitamin d2& vitamin e, chloramphenicol & hydrocortisone acetate, haemorroidal veinous plexus, dexamethasone & framycetin sulfate & gramicidin, dibucaine hcl & esculin & framycetin sulfate & hydrocortisone, betamethasone valerate & neomycin sulfate, betamethasone valerate & gentamicin sulfate, prednisolone acetate & sulfacetamide sodium, ascorbic acid & chlorpheniramine maleate & prednisone acetate, neomycin sulfate & prednisolone acetate & sulfacetamide sodium, gramicidin & neomycin sulfate & triamcinolone acetonide, methylprednisolone, acetylsalicylic acid & methyltestosterone, methylprednisolone sulfate & neomycin sulfate, hydrocortisone acetate & neomycin sulfate, aluminum chlorohydrate & methylprednisolone acetate & neomycin sulfate & sulfur, gramicidin & neomycin sulfate & nystatin & triamcinolone acetonide, hydrocortisone acetate & zinc oxide, hydrocortisone acetate & pramoxine hcl & zinc sulfate, aluminum chlorohydrate & methylprednisolone acetate & sulfur, hydrocortisone acetate & zinc oxide, hydrocortisone acetate & pramoxine hcl & zinc sulfate, desonide, clobetasol propionate, beclomethasone dipropionate & clioquinol, bacitracin zinc & hydrocortisone & neomycin sulfate & polymyxin b sulfate, hydrocortisone & neomycin sulfate & polymyxin b sulfate, gramicidin & neomycin sulfate & nystatin & triamcinolone acetonide, fluorometholone & polyvinyl alcohol, aluminum chlorohydrate & methylprednisolone acetate & sulfur, fluorometholone, lidocaine hcl & methylprednisolone acetate, flumetasone pivalate & salicylic acid, fluorometholone, lidocaine hcl & methylprednisolone acetate, aclometasone dipropionate, allantoin & chloramphenicol & hydrocortisone, amcinonide, atropine sulfate & prednisolone acetate, bacitracin & hydrocortisone & neomycin sulfate & polymyxin b sulfate, benzalkonium & dexamethasone & tobramycin , benzocaine & hydrocortisone acetate & zinc sulfate, betamethasone & sulfacetamide sodium, betamethasone acetate & betamethasone sodium phosphate, betamethasone benzoate, betamethasone dipropionate, betamethasone dipropionate &calcipotriene, betamethasone dipropionate &clotrimazole, betamethasone dipropionate & gentamicin sulfate, betamethasone dipropionate & salicylic acid, betamethasone disodium phosphate, betamethasone valerate & salicylic acid, betamethasone valerate & gentamycin sulfate, betamethasone valerate & neomycin sulfate, budesonide, camphor & hydrocortisone & menthol, chlorbutol & dexamethasone &tobramycin, ciclesonide.

[j] Total number of healthcare visits/tests in the 12-month period before the cohort entry date.

[k] The most recent eGFR measurement in the 365-day period before the cohort entry date (including the cohort entry date); eGFR was calculated using the new Chronic Kidney Disease (CKD)–Epidemiology (EPI) equation: $142 \times \min([\text{serum creatinine concentration in } \mu\text{mol/L}/88.4]/\kappa, 1)^{\alpha} \times \max([\text{serum creatinine concentration in } \mu\text{mol/L}/88.4]/\kappa, 1)^{-1.200} \times 0.9938^{\text{Age}} \times 1.012$ [if female]; κ=0.7 if female and 0.9 if male; α=-0.241 if female and -0.302 if male; min=the minimum of serum creatinine concentration/κ or 1; max=the maximum of serum creatinine concentration/κ or 1.

© 2023 Muanda FT et al. *JAMA Network Open.*

eTable 13. Baseline characteristics[a] of older adults with an eGFR between 45 and <60 mL/min/1.73m^2 newly prescribed low-dose methotrexate (LD MTX) vs those newly prescribed hydroxychloroquine (HCQ) in Ontario, Canada (2008–2021)

		Unmatched data (N=4661)					Matched data (N=3072)[b]				
		LD-MTX (n=2018)		HCQ (n=2643)		Standardized Difference[c]	LD-MTX (n=1536)		HCQ (n=1536)		Standardized Difference[c]
**Demographics**											
Age at cohort entry	Mean ± SD	76.6	6.8	76.2	6.6	7%	76.6	6.7	76.6	6.6	0%
	Median (IQR)	76	(71-82)	76	(71-81)	.	76	(71-81)	76	(71-81)	.
	66-<70	359	17.8%	492	18.6%	2%	265	17.3%	251	16.3%	3%
	70-<75	491	24.3%	672	25.4%	3%	374	24.3%	397	25.8%	3%
	75-<80	490	24.3%	655	24.8%	1%	392	25.5%	377	24.5%	2%
	80-<85	370	18.3%	498	18.8%	1%	286	18.6%	302	19.7%	3%
	85-<90	239	11.8%	249	9.4%	8%	169	11.0%	156	10.2%	3%
	90+	69	3.4%	77	2.9%	3%	50	3.3%	53	3.5%	1%
sex	F	1327	65.8%	1939	73.4%	17%	1073	69.9%	1070	69.7%	0%
	M	691	34.2%	704	26.6%	17%	463	30.1%	466	30.3%	0%
Year of cohort entry	2008	48	2.4%	67	2.5%	1%	40	2.6%	34	2.2%	3%
	2009	133	6.6%	150	5.7%	4%	100	6.5%	92	6.0%	2%
	2010	146	7.2%	164	6.2%	4%	104	6.8%	109	7.1%	1%
	2011	149	7.4%	170	6.4%	4%	104	6.8%	105	6.8%	0%
	2012	155	7.7%	159	6.0%	7%	112	7.3%	115	7.5%	1%
	2013	150	7.4%	208	7.9%	2%	116	7.6%	118	7.7%	0%
	2014	140	6.9%	193	7.3%	2%	102	6.6%	106	6.9%	1%
	2015	130	6.4%	195	7.4%	4%	103	6.7%	105	6.8%	0%
	2016	154	7.6%	182	6.9%	3%	120	7.8%	115	7.5%	1%
	2017	156	7.7%	209	7.9%	1%	111	7.2%	112	7.3%	0%

© 2023 Muanda FT et al. *JAMA Network Open.*

		Unmatched data (N=4661)					Matched data (N=3072)[b]				
		LD-MTX (n=2018)		HCQ (n=2643)		Standardized Difference[c]	LD-MTX (n=1536)		HCQ (n=1536)		Standardized Difference[c]
	2018	177	8.8%	261	9.9%	4%	141	9.2%	136	8.9%	1%
	2019	184	9.1%	257	9.7%	2%	143	9.3%	151	9.8%	2%
	2020	164	8.1%	247	9.3%	4%	123	8.0%	123	8.0%	0%
	2021	132	6.5%	181	6.8%	1%	117	7.6%	115	7.5%	0%
Location	Urban	1741	86.3%	2331	88.2%	6%	1344	87.5%	1322	86.1%	4%
	Rural	277	13.7%	312	11.8%	6%	192	12.5%	214	13.9%	4%
Residence	1	20	1.0%	19	0.7%	3%	14	0.9%	14	0.9%	0%
LHIN	1	109	5.4%	119	4.5%	4%	76	4.9%	77	5.0%	0%
	2	166	8.2%	156	5.9%	9%	117	7.6%	120	7.8%	1%
	3	119	5.9%	145	5.5%	2%	91	5.9%	92	6.0%	0%
	4	304	15.1%	577	21.8%	17%	270	17.6%	284	18.5%	2%
	5	135	6.7%	132	5.0%	7%	103	6.7%	95	6.2%	2%
	6	140	6.9%	160	6.1%	3%	107	7.0%	112	7.3%	1%
	7	105	5.2%	142	5.4%	1%	84	5.5%	79	5.1%	2%
	8	172	8.5%	287	10.9%	8%	145	9.4%	142	9.2%	1%
	9	205	10.2%	264	10.0%	1%	156	10.2%	148	9.6%	2%
	10	124	6.1%	94	3.6%	12%	70	4.6%	77	5.0%	2%
	11	250	12.4%	289	10.9%	5%	175	11.4%	168	10.9%	2%
	12	62	3.1%	135	5.1%	10%	51	3.3%	50	3.3%	0%
	13	87	4.3%	112	4.2%	0%	72	4.7%	65	4.2%	2%
	14	40	2.0%	31	1.2%	6%	19	1.2%	27	1.8%	5%
Socio-economic status[d]	1	388	19.2%	497	18.8%	1%	292	19.0%	282	18.4%	2%

© 2023 Muanda FT et al. *JAMA Network Open.*

		Unmatched data (N=4661)					Matched data (N=3072)[b]				
		LD-MTX (n=2018)		HCQ (n=2643)		Standardized Difference[c]	LD-MTX (n=1536)		HCQ (n=1536)		Standardized Difference[c]
	2	425	21.1%	566	21.4%	1%	329	21.4%	324	21.1%	1%
	3	440	21.8%	558	21.1%	2%	339	22.1%	335	21.8%	1%
	4	369	18.3%	524	19.8%	4%	285	18.6%	303	19.7%	3%
	5	396	19.6%	498	18.8%	2%	291	18.9%	292	19.0%	0%
**Prescriber**											
Rheumatologist		1092	54.1%	1654	62.6%	17%	924	60.2%	938	61.1%	2%
General practitioner		286	14.2%	389	14.7%	1%	221	14.4%	220	14.3%	0%
Internist		141	7.0%	176	6.7%	1%	116	7.6%	114	7.4%	1%
Dermatologist		228	11.3%	80	3.0%	33%	68	4.4%	71	4.6%	1%
Other		106	5.3%	122	4.6%	3%	71	4.6%	70	4.6%	0%
Missing		165	8.2%	222	8.4%	1%	136	8.9%	123	8.0%	3%
**Comorbidities[e]**											
Acute kidney injury		97	4.8%	96	3.6%	6%	55	3.6%	59	3.8%	1%
Alcoholism		19	0.9%	25	0.9%	0%	14	0.9%	12	0.8%	1%
Angina		329	16.3%	437	16.5%	1%	233	15.2%	242	15.8%	2%
Atrial fibrillation/flutter		132	6.5%	157	5.9%	2%	86	5.6%	98	6.4%	3%
Bipolar disorder		41	2.0%	58	2.2%	1%	30	2.0%	25	1.6%	3%
Chronic liver disease		76	3.8%	134	5.1%	6%	56	3.6%	58	3.8%	1%
Chronic obstructive pulmonary disease		572	28.3%	777	29.4%	2%	447	29.1%	432	28.1%	2%
Cirrhosis		49	2.4%	92	3.5%	7%	37	2.4%	38	2.5%	1%
Coronary artery disease (minus angina)		532	26.4%	675	25.5%	2%	393	25.6%	395	25.7%	0%

© 2023 Muanda FT et al. *JAMA Network Open.*

	Unmatched data (N=4661)					Matched data (N=3072)[b]				
	LD-MTX (n=2018)		HCQ (n=2643)		Standardized Difference[c]	LD-MTX (n=1536)		HCQ (n=1536)		Standardized Difference[c]
Dementia	165	8.2%	199	7.5%	3%	127	8.3%	120	7.8%	2%
Anemia	554	27.5%	718	27.2%	1%	417	27.1%	402	26.2%	2%
Glaucoma	217	10.8%	320	12.1%	4%	176	11.5%	176	11.5%	0%
Major hemorrhage	117	5.8%	152	5.8%	0%	87	5.7%	87	5.7%	0%
Congestive heart failure	288	14.3%	354	13.4%	3%	211	13.7%	217	14.1%	1%
Hypertension	1556	77.1%	2091	79.1%	5%	1194	77.7%	1195	77.8%	0%
Hypokalemia	36	1.8%	45	1.7%	1%	25	1.6%	24	1.6%	0%
Hyponatremia	35	1.7%	41	1.6%	1%	21	1.4%	24	1.6%	2%
Hypothyroidism	288	14.3%	382	14.5%	1%	213	13.9%	213	13.9%	0%
Migraine	55	2.7%	113	4.3%	9%	44	2.9%	43	2.8%	1%
Obesity	113	5.6%	163	6.2%	3%	83	5.4%	90	5.9%	2%
Parkinson disease	30	1.5%	30	1.1%	4%	17	1.1%	19	1.2%	1%
Peripheral vascular disease	24	1.2%	34	1.3%	1%	16	1.0%	20	1.3%	3%
Schizophrenia	31	1.5%	38	1.4%	1%	24	1.6%	21	1.4%	2%
Ischaemic stroke	29	1.4%	35	1.3%	1%	18	1.2%	20	1.3%	1%
Depression	148	7.3%	255	9.6%	8%	121	7.9%	114	7.4%	2%
Rheumatoid arthritis	1044	51.7%	1274	48.2%	7%	852	55.5%	855	55.7%	0%
Syncope	36	1.8%	32	1.2%	5%	24	1.6%	22	1.4%	2%
Arrhythmia	187	9.3%	221	8.4%	3%	125	8.1%	133	8.7%	2%
Inflammatory bowel disease	39	1.9%	22	0.8%	10%	14	0.9%	18	1.2%	3%
Major cancer[f]	799	39.6%	1032	39.0%	1%	598	38.9%	604	39.3%	1%

© 2023 Muanda FT et al. *JAMA Network Open.*

	Unmatched data (N=4661)					Matched data (N=3072)[b]				
	LD-MTX (n=2018)		HCQ (n=2643)		Standardized Difference[c]	LD-MTX (n=1536)		HCQ (n=1536)		Standardized Difference[c]
Prostatic hyperplasia	217	10.8%	250	9.5%	4%	151	9.8%	158	10.3%	2%
Fracture	140	6.9%	205	7.8%	3%	107	7.0%	113	7.4%	2%
Falls	84	4.2%	116	4.4%	1%	63	4.1%	64	4.2%	1%
Hyperkaliema	11	0.5%	17	0.6%	1%	9	0.6%	9	0.6%	0%
Prostatitis	43	2.1%	48	1.8%	2%	31	2.0%	31	2.0%	0%
Hypotension	34	1.7%	36	1.4%	2%	24	1.6%	19	1.2%	3%
Gallstones /biliary stones	98	4.9%	112	4.2%	3%	67	4.4%	62	4.0%	2%
Prior pneumotoxcity	68	3.4%	96	3.6%	1%	53	3.5%	54	3.5%	0%
Prior myelotoxicity	18	0.9%	20	0.8%	1%	10	0.7%	8	0.5%	3%
Prior sepsis	27	1.3%	31	1.2%	1%	18	1.2%	18	1.2%	0%
Prior methotrexate toxicity	94	4.7%	139	5.3%	3%	70	4.6%	72	4.7%	0%
Psoriasis	325	16.1%	85	3.2%	45%	98	6.4%	82	5.3%	5%
Dermatomyositis	133	6.6%	373	14.1%	25%	123	8.0%	122	7.9%	0%
Sarcoidosis	18	0.9%	13	0.5%	5%	10	0.7%	11	0.7%	0%
Systemic sclerosis or scleroderma	153	7.6%	202	7.6%	0%	112	7.3%	109	7.1%	1%
Systemic lupus erythematosus	183	9.1%	457	17.3%	24%	158	10.3%	152	9.9%	1%
Atopic dermatitis or eczema	629	31.2%	759	28.7%	5%	424	27.6%	414	27.0%	1%
Ulcerative colitis (UC)	40	2.0%	37	1.4%	5%	23	1.5%	23	1.5%	0%
Crohn disease	39	1.9%	22	0.8%	10%	16	1.0%	19	1.2%	2%
Acute urinary retention	38	1.9%	39	1.5%	3%	23	1.5%	27	1.8%	2%

© 2023 Muanda FT et al. *JAMA Network Open.*

		Unmatched data (N=4661)					Matched data (N=3072)[b]				
		LD-MTX (n=2018)		HCQ (n=2643)		Standardized Difference[c]	LD-MTX (n=1536)		HCQ (n=1536)		Standardized Difference[c]
Myocardial infarction		66	3.3%	77	2.9%	2%	44	2.9%	46	3.0%	1%
Dyslipidemia		526	26.1%	699	26.4%	1%	376	24.5%	386	25.1%	1%
Macula degeneration		92	4.6%	71	2.7%	10%	59	3.8%	61	4.0%	1%
Gastroesophageal reflux disease		520	25.8%	751	28.4%	6%	419	27.3%	417	27.1%	0%
Osteoarthritis		194	9.6%	310	11.7%	7%	153	10.0%	152	9.9%	0%
Major surgery		114	5.6%	148	5.6%	0%	81	5.3%	83	5.4%	0%
Prostate cancer		78	3.9%	80	3.0%	5%	47	3.1%	60	3.9%	4%
Diabete		466	23.1%	583	22.1%	2%	340	22.1%	357	23.2%	3%
Urinary tract infection		105	5.2%	147	5.6%	2%	79	5.1%	75	4.9%	1%
Gout		250	12.4%	357	13.5%	3%	197	12.8%	209	13.6%	2%
Modified Charlson comorbidity index[g]	Mean ± SD	2.43	1.09	2.33	0.98	10%	2.37	1.01	2.38	1.06	1%
	Median (IQR)	2	(2-2)	2	(2-2)	.	2	(2-2)	2	(2-2)	.
	2	1626	80.6%	2230	84.4%	10%	1272	0.828	1280	83.3%	1%
	3+	392	19.4%	413	15.6%	10%	264	0.172	256	16.7%	1%
**Medication use[h]**											
Alpha-adrenergic blocking agents		57	2.8%	60	2.3%	3%	14	0.9%	14	0.9%	0%
Anti-arrhythmic		26	1.3%	15	0.6%	7%	428	27.9%	421	27.4%	1%
Antibiotics		579	28.7%	714	27.0%	4%	457	29.8%	437	28.5%	3%
Ace inhibitor		580	28.7%	800	30.3%	4%	174	11.3%	191	12.4%	3%
Anticoagulants		233	11.5%	315	11.9%	1%	134	8.7%	121	7.9%	3%
Anticonvulsants		164	8.1%	248	9.4%	5%	486	31.6%	495	32.2%	1%

© 2023 Muanda FT et al. *JAMA Network Open.*

	Unmatched data (N=4661)					Matched data (N=3072)[b]				
	LD-MTX (n=2018)		HCQ (n=2643)		Standardized Difference[c]	LD-MTX (n=1536)		HCQ (n=1536)		Standardized Difference[c]
Angiotensin II receptor blockers	633	31.4%	836	31.6%	0%	12	0.8%	6	0.4%	5%
Aspirin	36	1.8%	56	2.1%	2%	30	2.0%	23	1.5%	4%
Antiplatelet agents	101	5.0%	172	6.5%	6%	73	4.8%	72	4.7%	0%
Antifungals	36	1.8%	51	1.9%	1%	27	1.8%	34	2.2%	3%
Tricyclic antidepressant	298	14.8%	428	16.2%	4%	224	14.6%	218	14.2%	1%
Gastrointestinal drugs	111	5.5%	129	4.9%	3%	84	5.5%	82	5.3%	1%
Beta-blockers	578	28.6%	803	30.4%	4%	440	28.6%	452	29.4%	2%
Bone Calcium regulators	31	1.5%	60	2.3%	6%	28	1.8%	34	2.2%	3%
Benzodiazepine	239	11.8%	374	14.2%	7%	194	12.6%	194	12.6%	0%
Bisphophonates	440	21.8%	474	17.9%	10%	341	22.2%	340	22.1%	0%
Beta agonist	198	9.8%	257	9.7%	0%	158	10.3%	161	10.5%	1%
H2 blockers	68	3.4%	104	3.9%	3%	54	3.5%	54	3.5%	0%
Channel calcium blockers	585	29.0%	842	31.9%	6%	464	30.2%	462	30.1%	0%
Cholinesterase inhibitors	31	1.5%	31	1.2%	3%	24	1.6%	16	1.0%	5%
Glucocorticoid[i]	1226	60.8%	1278	48.4%	25%	862	56.1%	867	56.4%	1%
Loop diuretics	267	13.2%	353	13.4%	1%	195	12.7%	198	12.9%	1%
Nitrates	74	3.7%	107	4.0%	2%	58	3.8%	57	3.7%	1%
Fibrates	37	1.8%	56	2.1%	2%	26	1.7%	26	1.7%	0%
NSAIDs (excluding ASA)	470	23.3%	708	26.8%	8%	390	25.4%	391	25.5%	0%
Insulin	129	6.4%	171	6.5%	0%	96	6.3%	105	6.8%	2%
Opioids	442	21.9%	610	23.1%	3%	358	23.3%	342	22.3%	2%

© 2023 Muanda FT et al. *JAMA Network Open.*

		Unmatched data (N=4661)					Matched data (N=3072)[b]				
		LD-MTX (n=2018)		HCQ (n=2643)		Standardized Difference[c]	LD-MTX (n=1536)		HCQ (n=1536)		Standardized Difference[c]
Over-active bladder medication		47	2.3%	100	3.8%	9%	41	2.7%	39	2.5%	1%
Potassium Sparing diuretics		112	5.6%	162	6.1%	2%	80	5.2%	92	6.0%	3%
Allopurinol		145	7.2%	243	9.2%	7%	118	7.7%	118	7.7%	0%
Anti-psychotics		43	2.1%	63	2.4%	2%	35	2.3%	31	2.0%	2%
Proton pump inhibitors		870	43.1%	1212	45.9%	6%	691	45.0%	677	44.1%	2%
5 alpha-reductase		121	6.0%	147	5.6%	2%	85	5.5%	88	5.7%	1%
Selective serotonin reuptake inhibitors		219	10.9%	315	11.9%	3%	174	11.3%	173	11.3%	0%
Statins		972	48.2%	1366	51.7%	7%	743	48.4%	736	47.9%	1%
Thiazide diuretics		380	18.8%	464	17.6%	3%	288	18.8%	283	18.4%	1%
Oral antidiabetics		430	21.3%	525	19.9%	3%	309	20.1%	327	21.3%	3%
Number of unique drug names	Mean ± SD	7.92	4.34	7.76	4.19	4%	7.84	4.33	7.79	419.0%	1%
	Median (IQR)	7	(5-10)	7	(5-10)	.	7	(5-10)	7	(5-10)	.
	0-4	426	21.1%	585	22.1%	2%	343	22.3%	336	21.9%	1%
	5-9	940	46.6%	1253	47.4%	2%	697	45.4%	728	47.4%	4%
	10-14	512	25.4%	632	23.9%	3%	392	25.5%	369	24.0%	3%
	15-19	102	5.1%	136	5.1%	0%	80	5.2%	83	5.4%	1%
	20+	38	1.9%	37	1.4%	4%	24	1.6%	20	1.3%	3%
Number of unique dins	Mean ± SD	8.5	4.86	8.31	4.7	4%	8.4	4.83	8.34	4.71	1%
	Median (IQR)	8	(5-11)	8	(5-11)	.	8	(5-11)	8	(5-11)	.
	0-4	396	19.6%	545	20.6%	2%	319	20.8%	313	20.4%	1%
	5-9	880	43.6%	1173	44.4%	2%	658	42.8%	680	44.3%	3%

© 2023 Muanda FT et al. *JAMA Network Open.*

		Unmatched data (N=4661)					Matched data (N=3072)[b]				
		LD-MTX (n=2018)		HCQ (n=2643)		Standardized Difference[c]	LD-MTX (n=1536)		HCQ (n=1536)		Standardized Difference[c]
	10-14	533	26.4%	680	25.7%	2%	402	26.2%	394	25.7%	1%
	15-19	151	7.5%	174	6.6%	4%	117	7.6%	107	7.0%	2%
	20+	58	2.9%	71	2.7%	1%	40	2.6%	42	2.7%	1%
**Healthcare use[j]**											
Primary care visits	Mean ± SD	10.04	8.94	9.92	8.71	1%	10.04	9.13	9.92	8.92	1%
	Median (IQR)	8	(5-13)	8	(5-13)	.	8	(5-13)	8	(5-13)	.
	0-4	465	23.0%	596	22.6%	1%	367	23.9%	361	23.5%	1%
	5-9	755	37.4%	1014	38.4%	2%	565	36.8%	575	37.4%	1%
	14-Oct	419	20.8%	550	20.8%	0%	311	20.2%	330	21.5%	3%
	15-19	184	9.1%	252	9.5%	1%	140	9.1%	136	8.9%	1%
	20+	195	9.7%	231	8.7%	3%	153	10.0%	134	8.7%	4%
Hospitalizations	Mean ± SD	0.17	0.53	0.13	0.47	8%	0.14	0.48	0.15	0.47	2%
	Median (IQR)	0	(0-0)	0	(0-0)	.	0	(0-0)	0	(0-0)	.
	0	1766	87.5%	2390	90.4%	9%	1376	89.6%	1371	89.3%	1%
	1	192	9.5%	184	7.0%	9%	124	8.1%	117	7.6%	2%
	2	34	1.7%	57	2.2%	4%	19	1.2%	40	2.6%	10%
	3+	26	1.3%	12	0.5%	8%	17	1.1%	8	0.5%	7%
Emergency departments visits	Mean ± SD	0.68	1.31	0.65	1.33	2%	0.66	1.3	0.64	1.24	2%
	Median (IQR)	0	(0-1)	0	(0-1)	.	0	(0-1)	0	(0-1)	.
	0	1301	64.5%	1741	65.9%	3%	998	65.0%	1008	65.6%	1%
	1	407	20.2%	508	19.2%	3%	309	20.1%	298	19.4%	2%
	2	166	8.2%	218	8.2%	0%	128	8.3%	127	8.3%	0%

© 2023 Muanda FT et al. *JAMA Network Open.*

		Unmatched data (N=4661)					Matched data (N=3072)[b]				
		LD-MTX (n=2018)		HCQ (n=2643)		Standardized Difference[c]	LD-MTX (n=1536)		HCQ (n=1536)		Standardized Difference[c]
	3+	144	7.1%	176	6.7%	2%	101	6.6%	103	6.7%	0%
TSH test		1391	68.9%	2008	76.0%	16%	1104	71.9%	1091	71.0%	2%
At-home physician service		47	2.3%	55	2.1%	1%	33	2.1%	37	2.4%	2%
Bone mineral density test		292	14.5%	449	17.0%	7%	247	16.1%	245	16.0%	0%
Cardiac catheterization		34	1.7%	25	0.9%	7%	19	1.2%	21	1.4%	2%
Cardiac stress test		276	13.7%	362	13.7%	0%	205	13.3%	202	13.2%	0%
Carotid ultrasound		99	4.9%	132	5.0%	0%	76	4.9%	71	4.6%	1%
Chest X-ray		925	45.8%	1054	39.9%	12%	667	43.4%	672	43.8%	1%
Cataract surgery		105	5.2%	139	5.3%	0%	85	5.5%	95	6.2%	3%
Cervical cancer screening		80	4.0%	96	3.6%	2%	59	3.8%	58	3.8%	0%
Colorectal cancer screening		323	16.0%	400	15.1%	2%	241	15.7%	231	15.0%	2%
Cholesterol test (total cholesterol, HDL)		1302	64.5%	1762	66.7%	5%	982	63.9%	966	62.9%	2%
CT abdomen		227	11.2%	251	9.5%	6%	164	10.7%	166	10.8%	0%
CT extremities		20	1.0%	40	1.5%	5%	19	1.2%	15	1.0%	2%
CT head		183	9.1%	224	8.5%	2%	133	8.7%	136	8.9%	1%
CT neck		31	1.5%	30	1.1%	4%	18	1.2%	18	1.2%	0%
CT pelvis		212	10.5%	239	9.0%	5%	152	9.9%	157	10.2%	1%
CT spine		34	1.7%	57	2.2%	4%	26	1.7%	26	1.7%	0%
CT thorax		218	10.8%	287	10.9%	0%	160	10.4%	159	10.4%	0%
Echocardiography		486	24.1%	648	24.5%	1%	359	23.4%	362	23.6%	0%

© 2023 Muanda FT et al. *JAMA Network Open.*

		Unmatched data (N=4661)					Matched data (N=3072)[b]				
		LD-MTX (n=2018)		HCQ (n=2643)		Standardized Difference[c]	LD-MTX (n=1536)		HCQ (n=1536)		Standardized Difference[c]
Flu shot		979	48.5%	1279	48.4%	0%	752	49.0%	742	48.3%	1%
Cystoscopy		84	4.2%	111	4.2%	0%	61	4.0%	67	4.4%	2%
Hearing test		84	4.2%	138	5.2%	5%	65	4.2%	69	4.5%	1%
Mammography		247	12.2%	428	16.2%	11%	213	13.9%	206	13.4%	1%
Prostate-specific antigen (PSA) test		31	1.5%	30	1.1%	4%	21	1.4%	21	1.4%	0%
Holter monitoring		171	8.5%	244	9.2%	2%	134	8.7%	133	8.7%	0%
Parathyroid hormone testing		146	7.2%	322	12.2%	17%	132	8.6%	134	8.7%	0%
Pulmonary function test		244	12.1%	350	13.2%	3%	185	12.0%	190	12.4%	1%
Urinalysis		1100	54.5%	1581	59.8%	11%	864	56.3%	837	54.5%	4%
**Laboratory measurement**											
eGFR[K]	Mean ± SD	53.46	4.23	53.5	4.25	1%	53.5	4.2	53.47	4.29	1%
	Median (IQR)	54	(50-57)	54	(50-57)	.	54	(50-57)	54	(50-57)	.
Urine ACR available		669	33.2%	945	35.8%	5%	507	33.0%	506	32.9%	0%
	Missing	1349	66.8%	1698	64.2%	5%	1029	67.0%	1030	67.1%	0%
Baseline ACR categories, µg/mg	<3	446	22.1%	675	25.5%	8%	345	22.5%	357	23.2%	2%
	3-30	183	9.1%	219	8.3%	3%	130	8.5%	122	7.9%	2%
	>30	40	2.0%	51	1.9%	1%	32	2.1%	27	1.8%	2%

© 2023 Muanda FT et al. *JAMA Network Open.*

Abbreviations: LD-MTX, low-dose methotrexate; HCQ, hydroxychloroquine; ACE inhibitor, angiotensin-converting-enzyme inhibitor; H2 blockers, Histamine H2-receptor antagonists; eGFR, estimated glomerular filtration rate; IQR, interquartile range; LHIN, Local Health Integration Network; ACR, urine albumin-to-creatinine ratio.

[a] Unless otherwise specified in the footnotes, baseline characteristics were assessed on the date the patient filled a low-dose methotrexate prescription or a hydroxychloroquine prescription—the cohort entry date.

[b] Propensity score matching technique was used to balance comparison groups on indicators of baseline health, including all known indications for methotrexate use (including off-label indications). The propensity score was estimated using multivariable logistic regression with 140 covariates chosen *a priori* (defined in eTable 8 in the Supplement). We use greedy matching, to match low-dose methotrexate drug user (1:1) to hydroxychloroquine user based on the logit of the propensity score (within a caliper of ±0.2 standard deviations).[24]

[c] the difference between the groups divided by the pooled SD; a value greater than 10% is interpreted as a meaningful difference.[25]

[d] Income was categorized into fifths of average neighborhood income on the cohort entry date.

[e] Baseline comorbidities were assessed in the 5-year period before the cohort entry date.

[f] Cancer includes the following types of cancer: skin, mouth (lip, tonsil, etc), throat, stomach, small/large intestine, liver, gall bladder, pancreas, breast, male/female reproductive organs, heart, lung, bone, urinary system (kidney, bladder, etc), endocrine glands, as well as leukemias and lymphomas

[g] Presence of kidney disease is a variable in the Charlson comorbidity index, which automatically results in all individuals receiving a minimum score of 2

[h] Medication use was examined in the 120-day period before the cohort entry date (the Ontario Drug Benefit program dispenses a maximum 100-day supply).

[I] Glucocorticoids included many medications regardless of their route of administration such as hydrocortisone acetate, dexamethasone, beclomethasone dipropionate, prednisone, hydrocortisone, flumetasone pivalate, clioquinol, betamethasone valerate, betamethasone, triamcinolone acetonide, triamcinolone diacetate, triamcinolone, flurandrenolide, betamethasone & dexamethasone sodium phosphate, cortisone acetate, dexamethasone tebutate, prednisolone, dexamethasone, corticotrophin, prednisolone acetate, fluocinolone acetonide, hydrocortisone sodium succinate, methylprednisolone sodium succinate, methylprednisolone acetate, methylprednisolone disodium phosphate, methylprednisolone, fluocinonide, betamethasone disodium phosphate, medrysone & polyvinyl alcohol, prednisolone acetate & sulfacetamide sodium, dexamethasone & neomycin sulfate & polymyxin b sulfate, clioquinol & flumetasone pivalate, clioquinol & hydrocortisone, 1,2-propanediol diacetate & acetic acid & benzethonium chloride & hydrocortisone, clioquinol & triamcinolone acetonide, flurandrenolide, fluocinolone acetonide, dexamethasone & neomycin sulfate, hydrocortisone & lidocaine hcl & neomycin sulfate, haemorroidal veinous plexus, prednisone & pheniramine maleate & inositol & phosphatidyl choline & vitamin a & vitamin d2& vitamin e, chloramphenicol & hydrocortisone acetate, haemorroidal veinous plexus, dexamethasone & framycetin sulfate & gramicidin, dibucaine hcl & esculin & framycetin sulfate & hydrocortisone, betamethasone valerate & neomycin sulfate, betamethasone valerate & gentamicin sulfate, prednisolone acetate & sulfacetamide sodium, ascorbic acid & chlorpheniramine maleate & prednisone acetate, neomycin sulfate & prednisolone acetate & sulfacetamide sodium, gramicidin & neomycin sulfate & triamcinolone acetonide, methylprednisolone, acetylsalicylic acid & methyltestosterone, methylprednisolone sulfate & neomycin sulfate, hydrocortisone acetate & neomycin sulfate, aluminum chlorohydrate & methylprednisolone acetate & neomycin sulfate & sulfur, gramicidin & neomycin sulfate & nystatin & triamcinolone acetonide, hydrocortisone acetate & zinc oxide, hydrocortisone acetate & pramoxine hcl & zinc sulfate, aluminum chlorohydrate & methylprednisolone acetate & sulfur, hydrocortisone acetate & zinc oxide, hydrocortisone acetate & pramoxine hcl & zinc sulfate, desonide, clobetasol propionate, beclomethasone dipropionate & clioquinol, bacitracin zinc & hydrocortisone & neomycin sulfate & polymyxin b sulfate, hydrocortisone & neomycin sulfate & polymyxin b sulfate, gramicidin & neomycin sulfate & nystatin & triamcinolone acetonide, fluorometholone & polyvinyl alcohol, aluminum chlorohydrate & methylprednisolone acetate & sulfur, fluorometholone, lidocaine hcl & methylprednisolone acetate, flumetasone pivalate & salicylic acid, fluorometholone, lidocaine hcl & methylprednisolone acetate, aclometasone dipropionate, allantoin & chloramphenicol & hydrocortisone, amcinonide,

© 2023 Muanda FT et al. *JAMA Network Open.*

atropine sulfate & prednisolone acetate, bacitracin & hydrocortisone & neomycin sulfate & polymyxin b sulfate, benzalkonium & dexamethasone & tobramycin , benzocaine & hydrocortisone acetate & zinc sulfate, betamethasone & sulfacetamide sodium, betamethasone acetate & betamethasone sodium phosphate, betamethasone benzoate, betamethasone dipropionate, betamethasone dipropionate &calcipotriene, betamethasone dipropionate &clotrimazole, betamethasone dipropionate & gentamicin sulfate, betamethasone dipropionate & salicylic acid, betamethasone disodium phosphate, betamethasone valerate & salicylic acid, betamethasone valerate & gentamycin sulfate, betamethasone valerate & neomycin sulfate, budesonide, camphor & hydrocortisone & menthol, chlorbutol & dexamethasone &tobramycin, ciclesonide.

[j] Total number of healthcare visits/tests in the 12-month period before the cohort entry date.

[k] The most recent eGFR measurement in the 365-day period before the cohort entry date (including the cohort entry date); eGFR was calculated using the new Chronic Kidney Disease (CKD)–Epidemiology (EPI) equation: 142× min([serum creatinine concentration in µmol/L/88.4]/κ, 1)$^{\alpha}$ × max([serum creatinine concentration in µmol/L/88.4]/κ, 1)$^{-1.200}$× 0.9938^{Age} × 1.012 [if female]; κ=0.7 if female and 0.9 if male; α=-0.241 if female and -0.302 if male; min=the minimum of serum creatinine concentration/κ or 1; max=the maximum of serum creatinine concentration/κ or 1.

© 2023 Muanda FT et al. *JAMA Network Open.*

eTable 14. Baseline characteristics[a] of older adults with an eGFR <45 mL/min/1.73m^2 newly prescribed low-dose methotrexate (LD MTX) vs those newly prescribed hydroxychloroquine (HCQ) in Ontario, Canada (2008–2021)

		Unmatched data (N=2248)					Matched data (N=1412)[b]				
		LD-MTX (n=882)		HCQ (n=1366)		Standardized difference[c]	LD-MTX (n=706)		HCQ (n=706)		Standardized difference[c]
**Demographics**											
Age at cohort entry	Mean ± SD	78.4	7.0	78.2	7.0	4%	78.6	7.0	78.6	7.01	1%
	Median (IQR)	78	(73-83)	78	(73-83)	.	78	(73-84)	78	(73-84)	.
	66-<70	115	13.0%	181	13.3%	1%	87	12.3%	85	12.0%	1%
	70-<75	158	17.9%	275	20.1%	6%	131	18.6%	133	18.8%	1%
	75-<80	213	24.1%	327	23.9%	0%	170	24.1%	167	23.7%	1%
	80-<85	212	24.0%	293	21.4%	6%	167	23.7%	163	23.1%	1%
	85-<90	131	<15%	219	16.0%	3%	107	15.2%	111	15.7%	1%
	90+	53	6.0%	71	5.2%	3%	44	6.2%	47	6.7%	2%
sex	F	591	67.0%	1019	74.6%	17%	495	70.1%	493	69.8%	1%
	M	291	33.0%	347	25.4%	17%	211	29.9%	213	30.2%	1%
Year of cohort entry	2008	33	3.7%	34	2.5%	7%	26	3.7%	24	3.4%	2%
	2009	66	7.5%	88	6.4%	4%	45	6.4%	52	7.4%	4%
	2010	81	9.2%	87	6.4%	10%	67	9.5%	65	9.2%	1%
	2011	81	9.2%	106	7.8%	5%	66	9.3%	65	9.2%	0%
	2012	81	9.2%	87	6.4%	10%	58	8.2%	55	7.8%	1%
	2013	66	7.5%	115	8.4%	3%	58	8.2%	57	8.1%	0%
	2014	71	8.0%	105	7.7%	1%	55	7.8%	52	7.4%	2%
	2015	54	6.1%	113	8.3%	9%	46	6.5%	43	6.1%	2%
	2016	52	5.9%	93	6.8%	4%	40	5.7%	48	6.8%	5%
	2017	62	7.0%	122	8.9%	7%	50	7.1%	51	7.2%	0%
	2018	66	7.5%	114	8.3%	3%	58	8.2%	56	7.9%	1%
	2019	66	7.5%	120	8.8%	5%	51	7.2%	50	7.1%	0%
	2020	50	5.7%	108	7.9%	9%	45	6.4%	42	5.9%	2%

© 2023 Muanda FT et al. *JAMA Network Open.*

		Unmatched data (N=2248)					Matched data (N=1412)[b]				
		LD-MTX (n=882)		HCQ (n=1366)		Standardized difference[c]	LD-MTX (n=706)		HCQ (n=706)		Standardized difference[c]
	2021	53	6.0%	74	5.4%	3%	41	5.8%	46	6.5%	3%
Location	Urban	776	88.0%	1182	86.5%	4%	618	87.5%	617	87.4%	0%
	Rural	106	12.0%	184	13.5%	4%	88	12.5%	89	12.6%	0%
Residence	Long term care	22	2.5%	18	1.3%	9%	11	1.6%	14	2.0%	3%
LHIN	1	50	5.7%	45	3.3%	12%	31	4.4%	31	4.4%	0%
	2	72	8.2%	111	8.1%	0%	51	7.2%	55	7.8%	2%
	3	59	6.7%	87	6.4%	1%	49	6.9%	46	6.5%	2%
	4	168	19.0%	294	21.5%	6%	148	21.0%	148	21.0%	0%
	5	56	6.3%	80	5.9%	2%	46	6.5%	49	6.9%	2%
	6	45	5.1%	79	5.8%	3%	40	5.7%	40	5.7%	0%
	7	34	3.9%	63	4.6%	3%	27	3.8%	29	4.1%	2%
	8	70	7.9%	139	10.2%	8%	58	8.2%	52	7.4%	3%
	9	84	9.5%	130	9.5%	0%	67	9.5%	67	9.5%	0%
	10	61	6.9%	57	4.2%	12%	43	6.1%	43	6.1%	0%
	11	110	12.5%	153	11.2%	4%	92	13.0%	83	11.8%	4%
	12	17	1.9%	45	3.3%	9%	14	2.0%	14	2.0%	0%
	13	40	4.5%	57	4.2%	1%	27	3.8%	35	5.0%	6%
	14	16	1.8%	26	1.9%	1%	13	1.8%	14	2.0%	1%
Socio-economic status[d]	1	178	20.2%	284	20.8%	1%	138	19.5%	155	22.0%	6%
	2	205	23.2%	308	22.5%	2%	159	22.5%	147	20.8%	4%
	3	179	20.3%	277	20.3%	0%	143	20.3%	148	21.0%	2%
	4	177	20.1%	256	18.7%	4%	147	20.8%	143	20.3%	1%
	5	143	16.2%	241	17.6%	4%	119	16.9%	113	16.0%	2%
**Prescriber, No. (%)**											
Rheumatologist		448	50.8%	817	59.8%	18%	400	56.7%	398	56.4%	1%
General practitioner		142	16.1%	230	16.8%	2%	113	16.0%	122	17.3%	3%

© 2023 Muanda FT et al. *JAMA Network Open.*

	Unmatched data (N=2248)					Matched data (N=1412)[b]				
	LD-MTX (n=882)		HCQ (n=1366)		Standardized difference[c]	LD-MTX (n=706)		HCQ (n=706)		Standardized difference[c]
Internist	69	7.8%	120	8.8%	4%	60	8.5%	54	7.6%	3%
Dermatologist	93	10.5%	36	2.6%	32%	37	5.2%	30	4.2%	5%
Other	52	5.9%	58	4.2%	8%	33	4.7%	39	5.5%	4%
Missing	78	8.8%	105	7.7%	4%	63	8.9%	63	8.9%	0%
**Comorbidities[e]**										
Acute kidney injury	107	12.1%	153	11.2%	3%	93	13.2%	88	12.5%	2%
Alcoholism	11	1.2%	20	1.5%	3%	9	1.3%	11	1.6%	3%
Angina	161	18.3%	249	18.2%	0%	125	17.7%	128	18.1%	1%
Atrial fibrillation/flutter	67	7.6%	123	9.0%	5%	58	8.2%	63	8.9%	3%
Bipolar disorder	12	1.4%	25	1.8%	3%	11	1.6%	11	1.6%	0%
Chronic liver disease	31	3.5%	67	4.9%	7%	27	3.8%	27	3.8%	0%
Chronic obstructive pulmonary disease	291	33.0%	426	31.2%	4%	221	31.3%	218	30.9%	1%
Cirrhosis	21	2.4%	49	3.6%	7%	18	2.5%	19	2.7%	1%
Coronary artery disease (minus angina)	288	32.7%	430	31.5%	3%	227	32.2%	218	30.9%	3%
Dementia	104	11.8%	130	9.5%	7%	74	10.5%	80	11.3%	3%
Anemia	310	35.1%	483	35.4%	1%	252	35.7%	270	38.2%	5%
Glaucoma	107	12.1%	156	11.4%	2%	84	11.9%	84	11.9%	0%
Major hemorrhage	67	7.6%	83	6.1%	6%	52	7.4%	49	6.9%	2%
Congestive heart failure	193	21.9%	314	23.0%	3%	161	22.8%	150	21.2%	4%
Hypertension	773	87.6%	1212	88.7%	3%	620	87.8%	615	87.1%	2%
Hypokalemia	30	3.4%	31	2.3%	7%	17	2.4%	20	2.8%	3%
Hyponatremia	20	2.3%	36	2.6%	2%	16	2.3%	23	3.3%	6%
Hypothyroidism	106	12.0%	192	14.1%	6%	86	12.2%	85	12.0%	1%
Migraine	32	3.6%	40	2.9%	4%	29	4.1%	22	3.1%	5%

© 2023 Muanda FT et al. *JAMA Network Open.*

	Unmatched data (N=2248)					Matched data (N=1412)[b]				
	LD-MTX (n=882)		HCQ (n=1366)		Standardized difference[c]	LD-MTX (n=706)		HCQ (n=706)		Standardized difference[c]
Obesity	57	6.5%	93	6.8%	1%	52	7.4%	45	6.4%	4%
Parkinson disease	15	1.7%	17	1.2%	4%	10	1.4%	10	1.4%	0%
Peripheral vascular disease	13	1.5%	13	1.0%	5%	11	1.6%	9	1.3%	3%
Schizophrenia	21	2.4%	29	2.1%	2%	15	2.1%	15	2.1%	0%
Ischaemic stroke	18	2.0%	16	1.2%	6%	14	2.0%	9	1.3%	5%
Depression	76	8.6%	109	8.0%	2%	59	8.4%	62	8.8%	1%
Rheumatoid arthritis	456	51.7%	648	47.4%	9%	387	54.8%	408	57.8%	6%
Syncope	14	1.6%	12	0.9%	6%	10	1.4%	11	1.6%	2%
Arrhythmia	95	10.8%	171	12.5%	5%	84	11.9%	81	11.5%	1%
Inflammatory bowel disease	12	1.4%	11	0.8%	6%	6	0.8%	10	1.4%	6%
Major cancer[f]	372	42.2%	585	42.8%	1%	300	42.5%	294	41.6%	2%
Prostatic hyperplasia	93	10.5%	114	8.3%	8%	64	9.1%	75	10.6%	5%
Fracture	73	8.3%	108	7.9%	1%	56	7.9%	57	8.1%	1%
Falls	57	6.5%	71	5.2%	6%	41	5.8%	44	6.2%	2%
Hyperkaliema	15	1.7%	29	2.1%	3%	13	1.8%	13	1.8%	0%
Prostatitis	18	2.0%	27	2.0%	0%	14	2.0%	11	1.6%	3%
Hypotension	31	3.5%	29	2.1%	8%	20	2.8%	21	3.0%	1%
Gallstones /biliary stones	46	5.2%	85	6.2%	4%	38	5.4%	35	5.0%	2%
Prior pneumotoxcity	74	8.4%	77	5.6%	11%	51	7.2%	51	7.2%	0%
Prior myelotoxicity	18	2.0%	21	1.5%	4%	13	1.8%	16	2.3%	4%
Prior sepsis	22	2.5%	27	2.0%	3%	17	2.4%	20	2.8%	3%
Prior methotrexate toxicity	102	11.6%	111	8.1%	12%	72	10.2%	76	10.8%	2%
Psoriasis	123	13.9%	45	3.3%	39%	48	6.8%	41	5.8%	4%

© 2023 Muanda FT et al. *JAMA Network Open.*

		Unmatched data (N=2248)					Matched data (N=1412)[b]				
		LD-MTX (n=882)		HCQ (n=1366)		Standardized difference[c]	LD-MTX (n=706)		HCQ (n=706)		Standardized difference[c]
Dermatomyositis		58	6.6%	173	12.7%	21%	53	7.5%	51	7.2%	1%
Sarcoidosis		12	1.4%	11	0.8%	6%	8	1.1%	7	1.0%	1%
Systemic sclerosis or scleroderma		68	7.7%	103	7.5%	1%	59	8.4%	55	7.8%	2%
Systemic lupus erythematosus		81	9.2%	207	15.2%	18%	73	10.3%	71	10.1%	1%
Atopic dermatitis or eczema		289	32.8%	395	28.9%	8%	213	30.2%	199	28.2%	4%
Ulcerative colitis (UC)		20	2.3%	19	1.4%	7%	14	2.0%	13	1.8%	1%
Crohn disease		18	2.0%	14	1.0%	8%	10	1.4%	9	1.3%	1%
Acute urinary retention		23	2.6%	32	2.3%	2%	20	2.8%	20	2.8%	0%
Myocardial infarction		41	4.6%	45	3.3%	7%	29	4.1%	25	3.5%	3%
Dyslipidemia		218	24.7%	310	22.7%	5%	169	23.9%	170	24.1%	0%
Macula degeneration		57	6.5%	61	4.5%	9%	39	5.5%	43	6.1%	3%
Gastroesophageal reflux disease		218	24.7%	350	25.6%	2%	172	24.4%	169	23.9%	1%
Osteoarthritis		90	10.2%	165	12.1%	6%	79	11.2%	76	10.8%	1%
Major surgery		57	6.5%	81	5.9%	2%	46	6.5%	44	6.2%	1%
Prostate cancer		29	3.3%	35	2.6%	4%	19	2.7%	22	3.1%	2%
Diabete		266	30.2%	407	29.8%	1%	208	29.5%	212	30.0%	1%
Urinary tract infection		95	10.8%	115	8.4%	8%	72	10.2%	67	9.5%	2%
Gout		168	19.0%	253	18.5%	1%	133	18.8%	122	17.3%	4%
Modified Charlson comorbidity index[g]	Mean ± SD	2.74	1.48	2.68	1.34	4%	2.73	1.46	2.72	1.37	1%
	Median (IQR)	2	(2-3)	2	(2-3)	.	2	(2-3)	2	(2-3)	.
	2	645	73.1%	988	72.3%	2%	519	73.5%	505	71.5%	4%
	3+	237	26.9%	378	27.7%	2%	187	26.5%	201	28.5%	4%
**Medication use[h]**											

© 2023 Muanda FT et al. *JAMA Network Open.*

	Unmatched data (N=2248)					Matched data (N=1412)[b]				
	LD-MTX (n=882)		HCQ (n=1366)		Standardized difference[c]	LD-MTX (n=706)		HCQ (n=706)		Standardized difference[c]
Alpha-adrenergic blocking agents	34	3.9%	73	5.3%	7%	22	3.1%	32	4.5%	7%
Anti-arrhythmic	13	1.5%	24	1.8%	2%	12	1.7%	17	2.4%	5%
Antibiotics	282	32.0%	413	30.2%	4%	217	30.7%	216	30.6%	0%
Ace inhibitor	259	29.4%	383	28.0%	3%	210	29.7%	221	31.3%	3%
Anticoagulants	123	13.9%	189	13.8%	0%	103	14.6%	103	14.6%	0%
Anticonvulsants	72	8.2%	157	11.5%	11%	66	9.3%	63	8.9%	1%
Angiotensin II receptor blockers	303	34.4%	543	39.8%	11%	258	36.5%	236	33.4%	7%
Aspirin	29	3.3%	35	2.6%	4%	21	3.0%	18	2.5%	3%
Antiplatelet agents	69	7.8%	103	7.5%	1%	50	7.1%	58	8.2%	4%
Antifungals	18	2.0%	22	1.6%	3%	14	2.0%	10	1.4%	5%
Tricyclic antidepressant	118	13.4%	248	18.2%	13%	100	14.2%	99	14.0%	1%
Gastrointestinal drugs	53	6.0%	62	4.5%	7%	39	5.5%	33	4.7%	4%
Beta-blockers	341	38.7%	560	41.0%	5%	267	37.8%	279	39.5%	3%
Bone Calcium regulators	19	2.2%	38	2.8%	4%	16	2.3%	17	2.4%	1%
Benzodiazepine	146	16.6%	195	14.3%	6%	114	16.1%	104	14.7%	4%
Bisphosphonates	178	20.2%	242	17.7%	6%	143	20.3%	129	18.3%	5%
Beta agonist	100	11.3%	143	10.5%	3%	74	10.5%	73	10.3%	1%
H2 blockers	42	4.8%	61	4.5%	1%	29	4.1%	31	4.4%	1%
Channel calcium blockers	377	42.7%	577	42.2%	1%	302	42.8%	299	42.4%	1%
Cholinesterase inhibitors	15	1.7%	23	1.7%	0%	9	1.3%	8	1.1%	2%
Glucocorticoid[i]	574	65.1%	720	52.7%	25%	425	60.2%	430	60.9%	1%
Loop diuretics	238	27.0%	361	26.4%	1%	192	27.2%	175	24.8%	5%

© 2023 Muanda FT et al. *JAMA Network Open.*

		Unmatched data (N=2248)					Matched data (N=1412)[b]				
		LD-MTX (n=882)		HCQ (n=1366)		Standardized difference[c]	LD-MTX (n=706)		HCQ (n=706)		Standardized difference[c]
Nitrates		60	6.8%	101	7.4%	2%	48	6.8%	55	7.8%	4%
Fibrates		20	2.3%	29	2.1%	1%	18	2.5%	15	2.1%	3%
NSAIDs (excluding ASA)		164	18.6%	257	18.8%	1%	137	19.4%	139	19.7%	1%
Insulin		104	11.8%	169	12.4%	2%	82	11.6%	86	12.2%	2%
Opioids		231	26.2%	392	28.7%	6%	192	27.2%	184	26.1%	2%
Over-active bladder medication		26	2.9%	49	3.6%	4%	22	3.1%	26	3.7%	3%
Potassium Sparing diuretics		65	7.4%	114	8.3%	3%	51	7.2%	65	9.2%	7%
Allopurinol		119	13.5%	207	15.2%	5%	96	13.6%	88	12.5%	3%
Anti-psychotics		23	2.6%	28	2.0%	4%	16	2.3%	18	2.5%	1%
Proton pump inhibitors		389	44.1%	651	47.7%	7%	317	44.9%	326	46.2%	3%
5 alpha-reductase		49	5.6%	81	5.9%	1%	37	5.2%	51	7.2%	8%
Selective serotonin reuptake inhibitors		106	12.0%	163	11.9%	0%	80	11.3%	78	11.0%	1%
Statins		467	52.9%	762	55.8%	6%	369	52.3%	391	55.4%	6%
Thiazide diuretics		189	21.4%	272	19.9%	4%	155	22.0%	141	20.0%	5%
Oral antidiabetics		212	24.0%	322	23.6%	1%	167	23.7%	160	22.7%	2%
Number of unique drug names	Mean ± SD	9.37	4.5	9.16	4.59	5%	9.17	4.37	9.17	4.65	0%
	Median (IQR)	9	(6-12)	9	(6-12)	.	9	(6-12)	9	(6-12)	.
	0-4	113	12.8%	191	14.0%	4%	94	13.3%	106	15.0%	5%
	5-9	362	41.0%	575	42.1%	2%	299	42.4%	285	40.4%	4%
	10-14	299	33.9%	441	32.3%	3%	239	33.9%	238	33.7%	0%
	15-19	88	10.0%	127	9.3%	2%	59	8.4%	61	8.6%	1%
	20+	20	2.3%	32	2.3%	0%	15	2.1%	16	2.3%	1%
Number of unique dins	Mean ± SD	10.05	5.06	9.82	5.17	4%	9.87	4.95	9.82	5.23	1%

© 2023 Muanda FT et al. *JAMA Network Open.*

		Unmatched data (N=2248)					Matched data (N=1412)[b]				
		LD-MTX (n=882)		HCQ (n=1366)		Standardized difference[c]	LD-MTX (n=706)		HCQ (n=706)		Standardized difference[c]
	Median (IQR)	10	(6-13)	9	(6-13)	.	10	(6-13)	9	(6-13)	.
	0-4	104	11.8%	180	13.2%	4%	86	12.2%	101	14.3%	6%
	5-9	323	36.6%	525	38.4%	4%	266	37.7%	257	36.4%	3%
	10-14	308	34.9%	441	32.3%	6%	250	35.4%	235	33.3%	4%
	15-19	103	11.7%	152	11.1%	2%	70	9.9%	81	11.5%	5%
	20+	44	5.0%	68	5.0%	0%	34	4.8%	32	4.5%	1%
**Healthcare use[j]**											
Primary care visits	Mean ± SD	11.43	9.91	11.02	11.69	4%	11.21	9.46	11.94	13.05	6%
	Median (IQR)	9	(5-14)	8	(5-14)	.	9	(5-14)	9	(5-14)	.
	0-4	166	18.8%	307	22.5%	9%	138	19.5%	136	19.3%	1%
	5-9	310	35.1%	460	33.7%	3%	249	35.3%	237	33.6%	4%
	10-14	192	21.8%	294	21.5%	1%	148	21.0%	158	22.4%	3%
	15-19	90	10.2%	142	10.4%	1%	70	9.9%	79	11.2%	4%
	20+	124	14.1%	163	11.9%	7%	101	14.3%	96	13.6%	2%
Hospitalizations	Mean ± SD	0.26	0.68	0.22	0.64	6%	0.25	0.65	0.26	0.74	1%
	Median (IQR)	0	(0-0)	0	(0-0)	.	0	(0-0)	0	(0-0)	.
Emergency departments visits	Mean ± SD	0.97	1.59	0.79	1.52	12%	0.92	1.52	0.9	1.67	1%
	Median (IQR)	0	(0-1)	0	(0-1)	.	0	(0-1)	0	(0-1)	.
TSH test		610	69.2%	990	72.5%	7%	496	70.3%	510	72.2%	4%
At-home physician service		25	2.8%	34	2.5%	2%	18	2.5%	17	2.4%	1%
Bone mineral density test		117	13.3%	208	15.2%	5%	91	12.9%	115	16.3%	10%
Cardiac stress test		121	13.7%	204	<15%	3%	94	13.3%	96	13.6%	1%
Carotid ultrasound		57	6.5%	61	4.5%	9%	43	6.1%	34	4.8%	6%
Chest X-ray		462	52.4%	640	46.9%	11%	360	51.0%	340	48.2%	6%

© 2023 Muanda FT et al. *JAMA Network Open.*

		Unmatched data (N=2248)					Matched data (N=1412)[b]				
		LD-MTX (n=882)		HCQ (n=1366)		Standardized difference[c]	LD-MTX (n=706)		HCQ (n=706)		Standardized difference[c]
Cataract surgery		54	6.1%	66	4.8%	6%	42	5.9%	34	4.8%	5%
Cervical cancer screening		24	2.7%	34	2.5%	1%	19	2.7%	15	2.1%	4%
Colorectal cancer screening		118	13.4%	207	15.2%	5%	93	13.2%	109	15.4%	6%
Cholesterol test (total cholesterol, HDL)		587	66.6%	901	66.0%	1%	467	66.1%	442	62.6%	7%
CT abdomen		110	12.5%	136	10.0%	8%	87	12.3%	72	10.2%	7%
CT extremities		11	1.2%	20	1.5%	3%	8	1.1%	10	1.4%	3%
CT head		101	11.5%	131	9.6%	6%	81	11.5%	64	9.1%	8%
CT pelvis		104	11.8%	122	8.9%	10%	83	11.8%	62	8.8%	10%
CT spine		19	2.2%	47	3.4%	7%	14	2.0%	22	3.1%	7%
CT thorax		117	13.3%	177	13.0%	1%	97	13.7%	103	14.6%	3%
Echocardiography		230	26.1%	398	29.1%	7%	185	26.2%	197	27.9%	4%
Flu shot		460	52.2%	672	49.2%	6%	378	53.5%	356	50.4%	6%
Cystoscopy		39	4.4%	61	4.5%	0%	31	4.4%	40	5.7%	6%
Hearing test		38	4.3%	72	5.3%	5%	36	5.1%	43	6.1%	4%
Mammography		76	8.6%	167	12.2%	12%	66	9.3%	63	8.9%	1%
Holter monitoring		78	8.8%	117	8.6%	1%	60	8.5%	61	8.6%	0%
Parathyroid hormone testing		181	20.5%	364	26.6%	14%	157	22.2%	158	22.4%	0%
Pulmonary function test		122	13.8%	194	14.2%	1%	98	13.9%	113	16.0%	6%
Urinalysis		562	63.7%	908	66.5%	6%	453	64.2%	454	64.3%	0%
**Laboratory measurement**											
eGFR[k]	Mean ± SD	37.01	6.73	36.02	7.02	14%	36.68	6.9	36.7	6.67	0%

© 2023 Muanda FT et al. *JAMA Network Open.*

		Unmatched data (N=2248)					Matched data (N=1412)[b]				
		LD-MTX (n=882)		HCQ (n=1366)		Standardized difference[c]	LD-MTX (n=706)		HCQ (n=706)		Standardized difference[c]
	Median (IQR)	39	(34-42)	38	(32-42)	.	38	(33-42)	38	(33-42)	.
Urine ACR available		404	45.8%	652	47.7%	4%	330	46.7%	326	46.2%	1%
	Missing	478	54.2%	714	52.3%	4%	376	53.3%	380	53.8%	1%
Baseline ACR categories, µg/mg	<3	204	23.1%	319	23.4%	1%	169	23.9%	152	21.5%	6%
	3-30	140	15.9%	214	15.7%	1%	117	16.6%	116	16.4%	1%
	>30	60	6.8%	119	8.7%	7%	44	6.2%	58	8.2%	8%

Abbreviations: LD-MTX, low-dose methotrexate; HCQ, hydroxychloroquine; ACE inhibitor, angiotensin-converting-enzyme inhibitor; H2 blockers, Histamine H2-receptor antagonists; eGFR, estimated glomerular filtration rate; IQR, interquartile range; LHIN, Local Health Integration Network; ACR, urine albumin-to-creatinine ratio.

[a] Unless otherwise specified in the footnotes, baseline characteristics were assessed on the date the patient filled a low-dose methotrexate prescription or a hydroxychloroquine prescription—the cohort entry date.

[b] Propensity score matching technique was used to balance comparison groups on indicators of baseline health, including all known indications for methotrexate use (including off-label indications). The propensity score was estimated using multivariable logistic regression with 80 covariates chosen *a priori* (defined in eTable 8 in the Supplement). We use greedy matching, to match low-dose methotrexate drug user (1:1) to hydroxychloroquine user based on the logit of the propensity score (within a caliper of ±0.2 standard deviations).[24]

[c] The difference between the groups divided by the pooled SD; a value greater than 10% is interpreted as a meaningful difference.[25]

[d] Income was categorized into fifths of average neighborhood income on the cohort entry date.

[e] Baseline comorbidities were assessed in the 5-year period before the cohort entry date.

[f] Cancer includes the following types of cancer: skin, mouth (lip, tonsil, etc), throat, stomach, small/large intestine, liver, gall bladder, pancreas, breast, male/female reproductive organs, heart, lung, bone, urinary system (kidney, bladder, etc), endocrine glands, as well as leukemias and lymphomas

[g] Presence of kidney disease is a variable in the Charlson comorbidity index, which automatically results in all individuals receiving a minimum score of 2

[h] Medication use was examined in the 120-day period before the cohort entry date (the Ontario Drug Benefit program dispenses a maximum 100-day supply).

[l] Glucocorticoids included many medications regardless of their route of administration such as hydrocortisone acetate, dexamethasone, beclomethasone dipropionate, prednisone, hydrocortisone, flumetasone pivalate, clioquinol, betamethasone valerate, betamethasone, triamcinolone acetonide, triamcinolone diacetate, triamcinolone, flurandrenolide, betamethasone & dexamethasone sodium phosphate, cortisone acetate, dexamethasone tebutate, prednisolone, dexamethasone, corticotrophin, prednisolone acetate, fluocinolone acetonide, hydrocortisone sodium succinate, methylprednisolone sodium succinate, methylprednisolone acetate, methylprednisolone disodium phosphate, methylprednisolone, fluocinonide, betamethasone disodium phosphate, medrysone &

© 2023 Muanda FT et al. *JAMA Network Open.*

polyvinyl alcohol, prednisolone acetate & sulfacetamide sodium, dexamethasone & neomycin sulfate & polymyxin b sulfate, clioquinol & flumetasone pivalate, clioquinol & hydrocortisone, 1,2-propanediol diacetate & acetic acid & benzethonium chloride & hydrocortisone, clioquinol & triamcinolone acetonide, flurandrenolide, fluocinolone acetonide, dexamethasone & neomycin sulfate, hydrocortisone & lidocaine hcl & neomycin sulfate, haemorroidal veinous plexus, prednisone & pheniramine maleate & inositol & phosphatidyl choline & vitamin a & vitamin d2& vitamin e, chloramphenicol & hydrocortisone acetate, haemorroidal veinous plexus, dexamethasone & framycetin sulfate & gramicidin, dibucaine hcl & esculin & framycetin sulfate & hydrocortisone, betamethasone valerate & neomycin sulfate, betamethasone valerate & gentamicin sulfate, prednisolone acetate & sulfacetamide sodium, ascorbic acid & chlorpheniramine maleate & prednisone acetate, neomycin sulfate & prednisolone acetate & sulfacetamide sodium, gramicidin & neomycin sulfate & triamcinolone acetonide, methylprednisolone, acetylsalicylic acid & methyltestosterone, methylprednisolone sulfate & neomycin sulfate, hydrocortisone acetate & neomycin sulfate, aluminum chlorohydrate & methylprednisolone acetate & neomycin sulfate & sulfur, gramicidin & neomycin sulfate & nystatin & triamcinolone acetonide, hydrocortisone acetate & zinc oxide, hydrocortisone acetate & pramoxine hcl & zinc sulfate, aluminum chlorohydrate & methylprednisolone acetate & sulfur, hydrocortisone acetate & zinc oxide, hydrocortisone acetate & pramoxine hcl & zinc sulfate, desonide, clobetasol propionate, beclomethasone dipropionate & clioquinol, bacitracin zinc & hydrocortisone & neomycin sulfate & polymyxin b sulfate, hydrocortisone & neomycin sulfate & polymyxin b sulfate, gramicidin & neomycin sulfate & nystatin & triamcinolone acetonide, fluorometholone & polyvinyl alcohol, aluminum chlorohydrate & methylprednisolone acetate & sulfur, fluorometholone, lidocaine hcl & methylprednisolone acetate, flumetasone pivalate & salicylic acid, fluorometholone, lidocaine hcl & methylprednisolone acetate, aclometasone dipropionate, allantoin & chloramphenicol & hydrocortisone, amcinonide, atropine sulfate & prednisolone acetate, bacitracin & hydrocortisone & neomycin sulfate & polymyxin b sulfate, benzalkonium & dexamethasone & tobramycin , benzocaine & hydrocortisone acetate & zinc sulfate, betamethasone & sulfacetamide sodium, betamethasone acetate & betamethasone sodium phosphate, betamethasone benzoate, betamethasone dipropionate, betamethasone dipropionate &calcipotriene, betamethasone dipropionate &clotrimazole, betamethasone dipropionate & gentamicin sulfate, betamethasone dipropionate & salicylic acid, betamethasone disodium phosphate, betamethasone valerate & salicylic acid, betamethasone valerate & gentamycin sulfate, betamethasone valerate & neomycin sulfate, budesonide, camphor & hydrocortisone & menthol, chlorbutol & dexamethasone &tobramycin, ciclesonide.

[j] Total number of healthcare visits/tests in the 12-month period before the cohort entry date.

[k] The most recent eGFR measurement in the 365-day period before the cohort entry date (including the cohort entry date); eGFR was calculated using the new Chronic Kidney Disease (CKD)–Epidemiology (EPI) equation: $142\times \min([\text{serum creatinine concentration in } \mu\text{mol/L}/88.4]/\kappa, 1)^{\alpha} \times \max([\text{serum creatinine concentration in } \mu\text{mol/L}/88.4]/\kappa, 1)^{-1.200}\times 0.9938^{Age} \times 1.012$ [if female]; κ=0.7 if female and 0.9 if male; α=-0.241 if female and -0.302 if male; min=the minimum of serum creatinine concentration/κ or 1; max=the maximum of serum creatinine concentration/κ or 1.

© 2023 Muanda FT et al. *JAMA Network Open.*

eTable 15. Risk of a hospital visit with myelosuppression, sepsis, pneumotoxicity, and hepatotoxicity in older adults with chronic kidney disease within 90 days of starting a new prescription for low-dose methotrexate at 15 to 35 mg/week vs a new prescription for hydroxychloroquine[a]

	Unmatched		Matched[b]				
	No. events (%)		No. events (%)				
	LD-MTX at 15 to 35 mg/week (n = 1510)	HCQ (n = 4009)	LD-MTX at 15 to 35 mg/week (n = 1357)	HCQ (n = 1357)	Risk difference, % (95% CI)	NNH, % (95% CI)	Risk ratio (95% CI)
**Primary outcome**							
Hospital visit with myelosuppression, sepsis, pneumotoxicity, and hepatotoxicity[c]	60 (3.97)	66 (1.65)	52 (3.83)	16 (1.18)	2.65 (1.49 to 3.82)	38 (26 to 67)	3.25 (1.87 to 5.64)

Abbreviations: LD-MTX, low-dose methotrexate; HCQ, hydroxychloroquine

[a] Reference group: hydroxychloroquine

[b] The propensity score was estimated using multivariable logistic regression with 140 covariates chosen *a priori* (defined in eTable 8 in the Supplement). We use greedy matching, to match low-dose methotrexate drug user at 5 to 35 mg/week (1:1) to hydroxychloroquine user based on the logit of the propensity score (within a caliper of ±0.2 standard deviations).[24] Risk ratios and 95% CIs were obtained using modified Poisson regression[26] and weighted risk differences and 95% CIs were obtained using a binomial regression model with an identity link function.

[c] The 90-day risk of a hospital visit with myelosuppression, sepsis, pneumotoxicity, and hepatotoxicity

© 2023 Muanda FT et al. *JAMA Network Open.*

eTable 16. Baseline characteristics[a] of older adults with chronic kidney disease newly prescribed low-dose methotrexate (LD MTX) at 15 to 35 mg/week vs those newly prescribed hydroxychloroquine (HCQ) in Ontario, Canada (2008–2021)

		Unmatched data (N=5519)					Matched data[b] (N=2714)				
		LD-MTX at 15 to 35 mg/week (n=1510)		HCQ (n=4009)		Standardized difference[c]	LD-MTX at 15 to 35 mg/week (n=1357)		HCQ (n=1357)		Standardized difference[c]
**Demographics**											
Age at cohort entry	Mean ± SD	76.3	6.7	76.9	6.8	9%	76.5	6.7	76.4	6.7	1%
	Median (IQR)	76	(71-81)	76	(71-82)	.	76	(71-81)	76	(71-81)	.
	66-<70	288	19.1%	673	16.8%	6%	244	18.0%	250	18.4%	1%
	70-<75	369	24.4%	947	23.6%	2%	330	24.3%	342	25.2%	2%
	75-<80	363	24.0%	982	24.5%	1%	332	24.5%	313	23.1%	3%
	80-<85	298	19.7%	791	19.7%	0%	269	19.8%	280	20.6%	2%
	85-<90	149	9.9%	468	11.7%	6%	141	10.4%	128	9.4%	3%
	90+	43	2.8%	148	3.7%	5%	41	3.0%	44	3.2%	1%
sex	F	983	65.1%	2958	73.8%	19%	911	67.1%	920	67.8%	1%
	M	527	34.9%	1051	26.2%	19%	446	32.9%	437	32.2%	1%
Year of cohort entry	2008	40	2.6%	101	2.5%	1%	35	2.6%	28	2.1%	3%
	2009	90	6.0%	238	5.9%	0%	79	5.8%	80	5.9%	0%
	2010	97	6.4%	251	6.3%	0%	89	6.6%	95	7.0%	2%
	2011	109	7.2%	276	6.9%	1%	98	7.2%	114	8.4%	4%
	2012	117	7.7%	246	6.1%	6%	98	7.2%	86	6.3%	4%
	2013	98	6.5%	323	8.1%	6%	93	6.9%	99	7.3%	2%
	2014	104	6.9%	298	7.4%	2%	91	6.7%	80	5.9%	3%
	2015	92	6.1%	308	7.7%	6%	83	6.1%	84	6.2%	0%
	2016	111	7.4%	275	6.9%	2%	102	7.5%	96	7.1%	2%
	2017	135	8.9%	331	8.3%	2%	126	9.3%	135	9.9%	2%
	2018	132	8.7%	375	9.4%	2%	118	8.7%	115	8.5%	1%
	2019	138	9.1%	377	9.4%	1%	123	9.1%	126	9.3%	1%

© 2023 Muanda FT et al. *JAMA Network Open.*

		Unmatched data (N=5519)					Matched data[b] (N=2714)				
		LD-MTX at 15 to 35 mg/week (n=1510)		HCQ (n=4009)		Standardized difference[c]	LD-MTX at 15 to 35 mg/week (n=1357)		HCQ (n=1357)		Standardized difference[c]
	2020	135	8.9%	355	8.9%	0%	116	8.5%	114	8.4%	0%
	2021	112	7.4%	255	6.4%	4%	106	7.8%	105	7.7%	0%
	N	1314	87.0%	3513	87.6%	2%	1178	86.8%	1179	86.9%	0%
Location	Y	196	13.0%	496	12.4%	2%	179	13.2%	178	13.1%	0%
Residence	1	18	1.2%	37	0.9%	3%	15	1.1%	16	1.2%	1%
	1	87	5.8%	164	4.1%	8%	74	5.5%	77	5.7%	1%
LHIN	2	123	8.1%	267	6.7%	5%	109	8.0%	102	7.5%	2%
	3	129	8.5%	232	5.8%	10%	106	7.8%	106	7.8%	0%
	4	249	16.5%	871	21.7%	13%	237	17.5%	241	17.8%	1%
	5	110	7.3%	212	5.3%	8%	99	7.3%	93	6.9%	2%
	6	118	7.8%	239	6.0%	7%	105	7.7%	101	7.4%	1%
	7	65	4.3%	205	5.1%	4%	58	4.3%	55	4.1%	1%
	8	117	7.7%	426	10.6%	10%	110	8.1%	104	7.7%	1%
	9	134	8.9%	394	9.8%	3%	122	9.0%	135	9.9%	3%
	10	105	7.0%	151	3.8%	14%	85	6.3%	85	6.3%	0%
	11	142	9.4%	442	11.0%	5%	132	9.7%	134	9.9%	1%
	12	40	2.6%	180	4.5%	10%	37	2.7%	38	2.8%	1%
	13	59	3.9%	169	4.2%	2%	54	4.0%	57	4.2%	1%
	14	32	2.1%	57	1.4%	5%	29	2.1%	29	2.1%	0%
	1	281	18.6%	781	19.5%	2%	249	18.3%	234	17.2%	3%
Socio-economic status[d]	2	328	21.7%	874	21.8%	0%	288	21.2%	308	22.7%	4%
	3	320	21.2%	835	20.8%	1%	287	21.1%	267	19.7%	3%
	4	288	19.1%	780	19.5%	1%	268	19.7%	281	20.7%	2%
	5	293	19.4%	739	18.4%	3%	265	19.5%	267	19.7%	1%
**Prescriber information**											

© 2023 Muanda FT et al. *JAMA Network Open.*

	Unmatched data (N=5519)					Matched data[b] (N=2714)				
	LD-MTX at 15 to 35 mg/week (n=1510)		HCQ (n=4009)		Standardized difference[c]	LD-MTX at 15 to 35 mg/week (n=1357)		HCQ (n=1357)		Standardized difference[c]
Rheumatologist	913	60.5%	2471	61.6%	2%	858	63.2%	859	63.3%	0%
General practitioner	191	12.6%	619	15.4%	8%	170	12.5%	186	13.7%	4%
Internist	99	6.6%	296	7.4%	3%	94	6.9%	108	8.0%	4%
Dermatologist	128	8.5%	116	2.9%	24%	72	5.3%	60	4.4%	4%
Other	64	4.2%	180	4.5%	1%	58	4.3%	51	3.8%	3%
Missing	115	7.6%	327	8.2%	2%	105	7.7%	93	6.9%	3%
**Comorbidities[e]**										
Acute kidney injury	88	5.8%	249	6.2%	2%	72	5.3%	64	4.7%	3%
Alcoholism	17	1.1%	45	1.1%	0%	15	1.1%	16	1.2%	1%
Angina	261	17.3%	686	17.1%	1%	233	17.2%	224	16.5%	2%
Atrial fibrillation/flutter	94	6.2%	280	7.0%	3%	86	6.3%	81	6.0%	1%
Bipolar disorder	32	2.1%	83	2.1%	0%	26	1.9%	23	1.7%	2%
Chronic liver disease	49	3.2%	201	5.0%	9%	46	3.4%	47	3.5%	1%
Chronic obstructive pulmonary disease	461	30.5%	1203	30.0%	1%	411	30.3%	411	30.3%	0%
Cirrhosis	30	2.0%	141	3.5%	9%	28	2.1%	33	2.4%	2%
Coronary artery disease (minus angina)	423	28.0%	1105	27.6%	1%	377	27.8%	379	27.9%	0%
Dementia	139	9.2%	329	8.2%	4%	120	8.8%	113	8.3%	2%
Anemia	448	29.7%	1201	30.0%	1%	404	29.8%	390	28.7%	2%
Glaucoma	176	11.7%	476	11.9%	1%	158	11.6%	155	11.4%	1%
Major hemorrhage	100	6.6%	235	5.9%	3%	93	6.9%	86	6.3%	2%
Congestive heart failure	238	15.8%	668	16.7%	2%	216	15.9%	207	15.3%	2%

© 2023 Muanda FT et al. *JAMA Network Open.*

	Unmatched data (N=5519)					Matched data[b] (N=2714)				
	LD-MTX at 15 to 35 mg/week (n=1510)		HCQ (n=4009)		Standardized difference[c]	LD-MTX at 15 to 35 mg/week (n=1357)		HCQ (n=1357)		Standardized difference[c]
Hypertension	1213	80.3%	3303	82.4%	5%	1092	80.5%	1089	80.3%	1%
Hypokalemia	31	2.1%	76	1.9%	1%	26	1.9%	22	1.6%	2%
Hyponatremia	22	1.5%	77	1.9%	3%	20	1.5%	16	1.2%	3%
Hypothyroidism	194	12.8%	574	14.3%	4%	177	13.0%	184	13.6%	2%
Migraine	40	2.6%	153	3.8%	7%	38	2.8%	31	2.3%	3%
Obesity	95	6.3%	256	6.4%	0%	80	5.9%	82	6.0%	0%
Parkinson disease	17	1.1%	47	1.2%	1%	15	1.1%	13	1.0%	1%
Peripheral vascular disease	19	1.3%	47	1.2%	1%	17	1.3%	19	1.4%	1%
Schizophrenia	23	1.5%	67	1.7%	2%	19	1.4%	19	1.4%	0%
Ischaemic stroke	22	1.5%	51	1.3%	2%	20	1.5%	20	1.5%	0%
Depression	114	7.5%	364	9.1%	6%	103	7.6%	109	8.0%	1%
Rheumatoid arthritis	803	53.2%	1922	47.9%	11%	746	55.0%	764	56.3%	3%
Syncope	28	1.9%	44	1.1%	7%	18	1.3%	17	1.3%	0%
Arrhythmia	141	9.3%	392	9.8%	2%	122	9.0%	121	8.9%	0%
Inflammatory bowel disease	25	1.7%	33	0.8%	8%	21	1.5%	9	0.7%	8%
Major cancer[f]	611	40.5%	1617	40.3%	0%	549	40.5%	544	40.1%	1%
Prostatic hyperplasia	181	12.0%	364	9.1%	9%	161	11.9%	150	11.1%	3%
Fracture	102	6.8%	313	7.8%	4%	94	6.9%	91	6.7%	1%
Falls	55	3.6%	187	4.7%	6%	50	3.7%	53	3.9%	1%
Hyperkaliema	11	0.7%	46	1.1%	4%	9	0.7%	9	0.7%	0%
Prostatitis	34	2.3%	75	1.9%	3%	29	2.1%	29	2.1%	0%
Hypotension	31	2.1%	65	1.6%	4%	25	1.8%	23	1.7%	1%
Gallstones /biliary stones	74	4.9%	197	4.9%	0%	65	4.8%	65	4.8%	0%

© 2023 Muanda FT et al. *JAMA Network Open.*

	Unmatched data (N=5519)					Matched data[b] (N=2714)				
	LD-MTX at 15 to 35 mg/week (n=1510)		HCQ (n=4009)		Standardized difference[c]	LD-MTX at 15 to 35 mg/week (n=1357)		HCQ (n=1357)		Standardized difference[c]
Prior pneumotoxcity	69	4.6%	173	4.3%	1%	57	4.2%	55	4.1%	1%
Prior myelotoxicity	15	1.0%	41	1.0%	0%	14	1.0%	13	1.0%	0%
Prior sepsis	24	1.6%	58	1.4%	2%	21	1.5%	19	1.4%	1%
Prior methotrexate toxicity	90	6.0%	250	6.2%	1%	77	5.7%	76	5.6%	0%
Psoriasis	218	14.4%	130	3.2%	40%	119	8.8%	112	8.3%	2%
Dermatomyositis	85	5.6%	546	13.6%	27%	84	6.2%	70	5.2%	4%
Sarcoidosis	13	0.9%	24	0.6%	3%	9	0.7%	13	1.0%	3%
Systemic sclerosis or scleroderma	109	7.2%	305	7.6%	2%	97	7.1%	93	6.9%	1%
Systemic lupus erythematosus	123	8.1%	664	16.6%	26%	118	8.7%	104	7.7%	4%
Atopic dermatitis or eczema	436	28.9%	1154	28.8%	0%	377	27.8%	346	25.5%	5%
Ulcerative colitis (UC)	28	1.9%	56	1.4%	4%	22	1.6%	16	1.2%	3%
Crohn disease	30	2.0%	36	0.9%	9%	25	1.8%	18	1.3%	4%
Acute urinary retention	27	1.8%	71	1.8%	0%	22	1.6%	21	1.5%	1%
Myocardial infarction	61	4.0%	122	3.0%	5%	51	3.8%	44	3.2%	3%
Dyslipidemia	375	24.8%	1009	25.2%	1%	338	24.9%	344	25.4%	1%
Macula degeneration	71	4.7%	132	3.3%	7%	58	4.3%	69	5.1%	4%
Gastroesophageal reflux disease	383	25.4%	1101	27.5%	5%	339	25.0%	330	24.3%	2%
Osteoarthritis	153	10.1%	475	11.8%	5%	140	10.3%	134	9.9%	1%
Major surgery	86	5.7%	229	5.7%	0%	76	5.6%	76	5.6%	0%
Prostate cancer	56	3.7%	115	2.9%	4%	49	3.6%	46	3.4%	1%
Diabete	393	26.0%	990	24.7%	3%	352	25.9%	343	25.3%	1%

© 2023 Muanda FT et al. *JAMA Network Open.*

		Unmatched data (N=5519)					Matched data[b] (N=2714)				
		LD-MTX at 15 to 35 mg/week (n=1510)		HCQ (n=4009)		Standardized difference[c]	LD-MTX at 15 to 35 mg/week (n=1357)		HCQ (n=1357)		Standardized difference[c]
Urinary tract infection		78	5.2%	262	6.5%	6%	69	5.1%	72	5.3%	1%
Gout		215	14.2%	610	15.2%	3%	197	14.5%	200	14.7%	1%
Modified Charlson comorbidity index[g]	Mean ± SD	2.47	1.16	2.45	1.13	2%	2.45	1.16	2.44	1.1	1%
	Median (IQR)	2	(2-2)	2	(2-2)	.	2	(2-2)	2	(2-2)	.
	2	1206	79.9%	3218	80.3%	1%	1099	81.0%	1095	80.7%	1%
	3+	304	20.1%	791	19.7%	1%	258	19.0%	262	19.3%	1%
**Medication use[h]**											
Alpha-adrenergic blocking agents		44	2.9%	133	3.3%	2%	36	2.7%	34	2.5%	1%
Anti-arrhythmic		25	1.7%	39	1.0%	6%	23	1.7%	20	1.5%	2%
Antibiotics		457	30.3%	1127	28.1%	5%	402	29.6%	403	29.7%	0%
Ace inhibitor		449	29.7%	1183	29.5%	0%	390	28.7%	388	28.6%	0%
Anticoagulants		200	13.2%	504	12.6%	2%	180	13.3%	196	14.4%	3%
Anticonvulsants		125	8.3%	405	10.1%	6%	117	8.6%	110	8.1%	2%
Angiotensin II receptor blockers		495	32.8%	1379	34.4%	3%	455	33.5%	442	32.6%	2%
Aspirin		26	1.7%	91	2.3%	4%	24	1.8%	18	1.3%	4%
Antiplatelet agents		93	6.2%	275	6.9%	3%	81	6.0%	70	5.2%	3%
Antifungals		23	1.5%	73	1.8%	2%	22	1.6%	21	1.5%	1%
Tricyclic antidepressant		225	<15%	676	16.9%	5%	201	14.8%	194	14.3%	1%
Gastrointestinal drugs		78	5.2%	191	4.8%	2%	68	5.0%	61	4.5%	2%
Beta-blockers		469	31.1%	1363	34.0%	6%	431	31.8%	444	32.7%	2%
Bone Calcium regulators		21	1.4%	98	2.4%	7%	19	1.4%	12	0.9%	5%
Benzodiazepine		198	13.1%	569	14.2%	3%	182	13.4%	180	13.3%	0%

© 2023 Muanda FT et al. *JAMA Network Open.*

	Unmatched data (N=5519)					Matched data[b] (N=2714)				
	LD-MTX at 15 to 35 mg/week (n=1510)		HCQ (n=4009)		Standardized difference[c]	LD-MTX at 15 to 35 mg/week (n=1357)		HCQ (n=1357)		Standardized difference[c]
Bisphosphonates	338	22.4%	716	17.9%	11%	305	22.5%	307	22.6%	0%
Beta agonist	158	10.5%	400	10.0%	2%	144	10.6%	144	10.6%	0%
H2 blockers	57	3.8%	165	4.1%	2%	51	3.8%	52	3.8%	0%
Channel calcium blockers	501	33.2%	1419	35.4%	5%	463	34.1%	455	33.5%	1%
Cholinesterase inhibitors	25	1.7%	54	1.3%	3%	20	1.5%	20	1.5%	0%
Glucocorticoid[i]	946	62.6%	1998	49.8%	26%	826	60.9%	827	60.9%	0%
Loop diuretics	234	15.5%	714	17.8%	6%	209	15.4%	205	15.1%	1%
Nitrates	62	4.1%	208	5.2%	5%	59	4.3%	57	4.2%	0%
Fibrates	31	2.1%	85	2.1%	0%	27	2.0%	27	2.0%	0%
NSAIDs (excluding ASA)	357	23.6%	965	24.1%	1%	331	24.4%	319	23.5%	2%
Insulin	109	7.2%	340	8.5%	5%	99	7.3%	93	6.9%	2%
Opioids	356	23.6%	1002	25.0%	3%	333	24.5%	311	22.9%	4%
Over-active bladder medication	40	2.6%	149	3.7%	6%	34	2.5%	37	2.7%	1%
Potassium Sparing diuretics	89	5.9%	276	6.9%	4%	82	6.0%	93	6.9%	4%
Allopurinol	126	8.3%	450	11.2%	10%	121	8.9%	110	8.1%	3%
Anti-psychotics	27	1.8%	91	2.3%	4%	25	1.8%	22	1.6%	2%
Proton pump inhibitors	655	43.4%	1863	46.5%	6%	597	44.0%	597	44.0%	0%
5 alpha-reductase	98	6.5%	228	5.7%	3%	88	6.5%	80	5.9%	2%
Selective serotonin reuptake inhibitors	177	11.7%	478	11.9%	1%	156	11.5%	164	12.1%	2%
Statins	750	49.7%	2128	53.1%	7%	680	50.1%	655	48.3%	4%

© 2023 Muanda FT et al. *JAMA Network Open.*

		Unmatched data (N=5519)					Matched data[b] (N=2714)				
		LD-MTX at 15 to 35 mg/week (n=1510)		HCQ (n=4009)		Standardized difference[c]	LD-MTX at 15 to 35 mg/week (n=1357)		HCQ (n=1357)		Standardized difference[c]
Thiazide diuretics		304	20.1%	736	18.4%	4%	266	19.6%	277	20.4%	2%
Oral antidiabetics		362	24.0%	847	21.1%	7%	322	23.7%	313	23.1%	1%
Number of unique drug names	Mean ± SD	8.33	4.37	8.24	4.38	2%	8.3	4.33	8.16	4.39	3%
	Median (IQR)	8	(5-11)	8	(5-11)	.	8	(5-11)	8	(5-11)	.
	0-4	294	19.5%	776	19.4%	0%	263	19.4%	273	20.1%	2%
	5-9	653	43.2%	1828	45.6%	5%	588	43.3%	617	45.5%	4%
	10-14	434	28.7%	1073	26.8%	4%	397	29.3%	361	26.6%	6%
	15-19	107	7.1%	263	6.6%	2%	91	6.7%	85	6.3%	2%
	20+	22	1.5%	69	1.7%	2%	18	1.3%	21	1.5%	2%
Number of unique dins	Mean ± SD	8.96	4.88	8.82	4.92	3%	8.9	4.81	8.75	4.97	3%
	Median (IQR)	8	(5-12)	8	(5-11)	.	8	(5-12)	8	(5-11)	.
	0-4	272	18.0%	725	18.1%	0%	245	18.1%	257	18.9%	2%
	5-9	606	40.1%	1698	42.4%	5%	548	40.4%	572	42.2%	4%
	10-14	437	28.9%	1121	28.0%	2%	395	29.1%	374	27.6%	3%
	15-19	146	9.7%	326	8.1%	6%	129	9.5%	109	8.0%	5%
	20+	49	3.2%	139	3.5%	2%	40	2.9%	45	3.3%	2%
**Healthcare use[j]**											
Primary care visits	Mean ± SD	10.31	9.01	10.3	9.84	0%	10.44	9.21	10.45	10.07	0%
	Median (IQR)	8	(5-13)	8	(5-13)	.	8	(5-13)	8	(5-13)	.
	0-4	335	22.2%	903	22.5%	1%	295	21.7%	305	22.5%	2%
	5-9	556	36.8%	1474	36.8%	0%	497	36.6%	479	35.3%	3%
	10-14	319	21.1%	844	21.1%	0%	290	21.4%	291	21.4%	0%
	15-19	142	9.4%	394	9.8%	1%	130	9.6%	143	10.5%	3%
	20+	158	10.5%	394	9.8%	2%	145	10.7%	139	10.2%	2%

© 2023 Muanda FT et al. *JAMA Network Open.*

		Unmatched data (N=5519)					Matched data[b] (N=2714)				
		LD-MTX at 15 to 35 mg/week (n=1510)		HCQ (n=4009)		. Standardized difference[c]	LD-MTX at 15 to 35 mg/week (n=1357)		HCQ (n=1357)		Standardized difference[c]
Hospitalizations	Mean ± SD	0.18	0.57	0.16	0.54	4%	0.18	0.58	0.16	0.5	4%
	Median (IQR)	0	(0-0)	0	(0-0)	.	0	(0-0)	0	(0-0)	.
	0	1322	87.5%	3550	88.6%	3%	1196	88.1%	1191	87.8%	1%
	1	132	8.7%	331	8.3%	1%	110	8.1%	122	9.0%	3%
	2	32	2.1%	93	2.3%	1%	28	2.1%	35	2.6%	3%
	3+	24	1.6%	35	0.9%	6%	23	1.7%	9	0.7%	9%
Emergency departments visits	Mean ± SD	0.72	1.35	0.7	1.4	1%	0.71	1.34	0.7	1.37	1%
	Median (IQR)	0	(0-1)	0	(0-1)	.	0	(0-1)	0	(0-1)	.
	0	976	64.6%	2593	64.7%	0%	881	64.9%	865	63.7%	3%
	1	276	18.3%	782	19.5%	3%	243	17.9%	276	20.3%	6%
	2	135	8.9%	327	8.2%	3%	119	8.8%	119	8.8%	0%
	3+	123	8.1%	307	7.7%	1%	114	8.4%	97	7.1%	5%
TSH test		1040	68.9%	2998	74.8%	13%	952	70.2%	972	71.6%	3%
At-home physician service		31	2.1%	89	2.2%	1%	27	2.0%	27	2.0%	0%
Bone mineral density test		214	14.2%	657	16.4%	6%	204	15.0%	197	14.5%	1%
Cardiac catheterization		22	1.5%	48	1.2%	3%	20	1.5%	15	1.1%	4%
Cardiac stress test		206	13.6%	566	14.1%	1%	189	13.9%	174	12.8%	3%
Carotid ultrasound		80	5.3%	193	4.8%	2%	67	4.9%	66	4.9%	0%
Chest X-ray		712	47.2%	1694	42.3%	10%	629	46.4%	638	47.0%	1%
Cataract surgery		80	5.3%	205	5.1%	1%	74	5.5%	72	5.3%	1%
Cervical cancer screening		60	4.0%	130	3.2%	4%	53	3.9%	64	4.7%	4%

© 2023 Muanda FT et al. *JAMA Network Open.*

		Unmatched data (N=5519)					Matched data[b] (N=2714)				
		LD-MTX at 15 to 35 mg/week (n=1510)		HCQ (n=4009)		Standardized difference[c]	LD-MTX at 15 to 35 mg/week (n=1357)		HCQ (n=1357)		Standardized difference[c]
Colorectal cancer screening		230	15.2%	607	15.1%	0%	198	14.6%	190	14.0%	2%
Cholesterol test (total cholesterol, HDL)		993	65.8%	2663	66.4%	1%	883	65.1%	880	64.8%	1%
CT abdomen		173	11.5%	387	9.7%	6%	155	11.4%	139	10.2%	4%
CT extremities		22	1.5%	60	1.5%	0%	20	1.5%	20	1.5%	0%
CT head		146	9.7%	355	8.9%	3%	125	9.2%	132	9.7%	2%
CT neck		21	1.4%	42	1.0%	4%	15	1.1%	21	1.5%	4%
CT pelvis		161	10.7%	361	9.0%	6%	143	10.5%	130	9.6%	3%
CT spine		30	2.0%	104	2.6%	4%	29	2.1%	26	1.9%	1%
CT thorax		151	10.0%	464	11.6%	5%	138	10.2%	143	10.5%	1%
Echocardiography		357	23.6%	1046	26.1%	6%	328	24.2%	330	24.3%	0%
Flu shot		704	46.6%	1951	48.7%	4%	636	46.9%	654	48.2%	3%
Cystoscopy		56	3.7%	172	4.3%	3%	49	3.6%	49	3.6%	0%
Hearing test		62	4.1%	210	5.2%	5%	57	4.2%	63	4.6%	2%
Mammography		192	12.7%	595	14.8%	6%	178	13.1%	171	12.6%	1%
Prostate-specific antigen (PSA) test		26	1.7%	36	0.9%	7%	19	1.4%	23	1.7%	2%
Holter monitoring		128	8.5%	361	9.0%	2%	116	8.5%	116	8.5%	0%
Parathyroid hormone testing		173	11.5%	686	17.1%	16%	167	12.3%	173	12.7%	1%
Pulmonary function test		188	12.5%	544	13.6%	3%	163	12.0%	179	13.2%	4%
Urinalysis		858	56.8%	2489	62.1%	11%	782	57.6%	796	58.7%	2%
**Laboratory measurement**											
eGFR[k]	Mean ± SD	49.42	8.46	47.54	9.87	20%	49.22	8.49	49.29	9.07	1%

© 2023 Muanda FT et al. *JAMA Network Open.*

		Unmatched data (N=5519)					Matched data[b] (N=2714)				
		LD-MTX at 15 to 35 mg/week (n=1510)		HCQ (n=4009)		. Standardized difference[c]	LD-MTX at 15 to 35 mg/week (n=1357)		HCQ (n=1357)		Standardized difference[c]
	Median (IQR)	51	(45-56)	50	(41-56)	.	51	(44-56)	52	(44-57)	.
Baseline eGFR categories	45-<60	1115	73.8%	2643	65.9%	17%	990	73.0%	980	72.2%	2%
	30-<45	342	22.6%	1100	27.4%	11%	317	23.4%	318	23.4%	0%
	<30	53	3.5%	266	6.6%	14%	50	3.7%	59	4.3%	3%
Urine ACR available		578	38.3%	1597	39.8%	3%	517	38.1%	512	37.7%	1%
	Missing	932	61.7%	2412	60.2%	3%	840	61.9%	845	62.3%	1%
Baseline ACR categories, µg/mg	<3	357	23.6%	994	24.8%	3%	313	23.1%	341	25.1%	5%
	3-30	172	11.4%	433	10.8%	2%	160	11.8%	136	10.0%	6%
	>30	49	3.2%	170	4.2%	5%	44	3.2%	35	2.6%	4%

Abbreviations: LD-MTX, low-dose methotrexate; HCQ, hydroxychloroquine; ACE inhibitor, angiotensin-converting-enzyme inhibitor; H2 blockers, Histamine H2-receptor antagonists; eGFR, estimated glomerular filtration rate; IQR, interquartile range; LHIN, Local Health Integration Network; ACR, urine albumin-to-creatinine ratio.

[a] Unless otherwise specified in the footnotes, baseline characteristics were assessed on the date the patient filled a low-dose methotrexate prescription or a hydroxychloroquine prescription—the cohort entry date.

[b] Propensity score matching technique was used to balance comparison groups on indicators of baseline health, including all known indications for methotrexate use (including off-label indications). The propensity score was estimated using multivariable logistic regression with 140 covariates chosen *a priori* (defined in eTable 8 in the Supplement. We use greedy matching, to match low-dose methotrexate drug user (1:1) to hydroxychloroquine user based on the logit of the propensity score (within a caliper of ±0.2 standard deviations).[24]

[c] The difference between the groups divided by the pooled SD; a value greater than 10% is interpreted as a meaningful difference.[25]

[d] Income was categorized into fifths of average neighborhood income on the cohort entry date.

[e] Baseline comorbidities were assessed in the 5-year period before the cohort entry date.

[f] Cancer includes the following types of cancer: skin, mouth (lip, tonsil, etc), throat, stomach, small/large intestine, liver, gall bladder, pancreas, breast, male/female reproductive organs, heart, lung, bone, urinary system (kidney, bladder, etc), endocrine glands, as well as leukemias and lymphomas

[g] Presence of kidney disease is a variable in the Charlson comorbidity index, which automatically results in all individuals receiving a minimum score of 2

[h] Medication use was examined in the 120-day period before the cohort entry date (the Ontario Drug Benefit program dispenses a maximum 100-day supply).

[i] Glucocorticoids included many medications regardless of their route of administration such as hydrocortisone acetate, dexamethasone, beclomethasone dipropionate, prednisone, hydrocortisone, flumetasone pivalate, clioquinol, betamethasone valerate, betamethasone, triamcinolone acetonide, triamcinolone diacetate, triamcinolone, flurandrenolide, betamethasone & dexamethasone sodium phosphate, cortisone acetate, dexamethasone tebutate, prednisolone, dexamethasone, corticotrophin, prednisolone acetate, fluocinolone acetonide, hydrocortisone sodium succinate, methylprednisolone sodium succinate, methylprednisolone acetate, methylprednisolone disodium phosphate, methylprednisolone, fluocinonide, betamethasone disodium phosphate, medrysone & polyvinyl alcohol, prednisolone acetate & sulfacetamide sodium, dexamethasone & neomycin sulfate & polymyxin b sulfate, clioquinol & flumetasone pivalate, clioquinol & hydrocortisone, 1,2-propanediol diacetate & acetic acid & benzethonium chloride & hydrocortisone, clioquinol & triamcinolone acetonide, flurandrenolide, fluocinolone acetonide, dexamethasone & neomycin sulfate, hydrocortisone & lidocaine hcl & neomycin sulfate, haemorroidal veinous plexus, prednisone & pheniramine maleate & inositol & phosphatidyl choline & vitamin a & vitamin d2& vitamin e, chloramphenicol & hydrocortisone acetate, haemorroidal veinous plexus, dexamethasone & framycetin sulfate & gramicidin, dibucaine hcl & esculin & framycetin sulfate & hydrocortisone, betamethasone valerate & neomycin sulfate, betamethasone valerate & gentamicin sulfate, prednisolone acetate & sulfacetamide sodium, ascorbic acid & chlorpheniramine maleate & prednisone acetate, neomycin sulfate & prednisolone acetate & sulfacetamide sodium, gramicidin & neomycin sulfate & triamcinolone acetonide, methylprednisolone, acetylsalicylic acid & methyltestosterone, methylprednisolone sulfate & neomycin sulfate, hydrocortisone acetate & neomycin sulfate, aluminum chlorohydrate & methylprednisolone acetate & neomycin sulfate & sulfur, gramicidin & neomycin sulfate & nystatin & triamcinolone acetonide, hydrocortisone acetate & zinc oxide, hydrocortisone acetate & pramoxine hcl & zinc sulfate, aluminum chlorohydrate & methylprednisolone acetate & sulfur, hydrocortisone acetate & zinc oxide, hydrocortisone acetate & pramoxine hcl & zinc sulfate, desonide, clobetasol propionate, beclomethasone dipropionate & clioquinol, bacitracin zinc & hydrocortisone & neomycin sulfate & polymyxin b sulfate, hydrocortisone & neomycin sulfate & polymyxin b sulfate, gramicidin & neomycin sulfate & nystatin & triamcinolone acetonide, fluorometholone & polyvinyl alcohol, aluminum chlorohydrate & methylprednisolone acetate & sulfur, fluorometholone, lidocaine hcl & methylprednisolone acetate, flumetasone pivalate & salicylic acid, fluorometholone, lidocaine hcl & methylprednisolone acetate, aclometasone dipropionate, allantoin & chloramphenicol & hydrocortisone, amcinonide, atropine sulfate & prednisolone acetate, bacitracin & hydrocortisone & neomycin sulfate & polymyxin b sulfate, benzalkonium & dexamethasone & tobramycin , benzocaine & hydrocortisone acetate & zinc sulfate, betamethasone & sulfacetamide sodium, betamethasone acetate & betamethasone sodium phosphate, betamethasone benzoate, betamethasone dipropionate, betamethasone dipropionate &calcipotriene, betamethasone dipropionate &clotrimazole, betamethasone dipropionate & gentamicin sulfate, betamethasone dipropionate & salicylic acid, betamethasone disodium phosphate, betamethasone valerate & salicylic acid, betamethasone valerate & gentamycin sulfate, betamethasone valerate & neomycin sulfate, budesonide, camphor & hydrocortisone & menthol, chlorbutol & dexamethasone &tobramycin, ciclesonide.

[j] Total number of healthcare visits/tests in the 12-month period before the cohort entry date.

[k] The most recent eGFR measurement in the 365-day period before the cohort entry date (including the cohort entry date); eGFR was calculated using the new Chronic Kidney Disease (CKD)–Epidemiology (EPI) equation: 142× min([serum creatinine concentration in µmol/L/88.4]/κ, 1)$^{\alpha}$ × max([serum creatinine concentration in µmol/L/88.4]/κ, 1)$^{-1.200}$× 0.9938^{Age} × 1.012 [if female]; κ=0.7 if female and 0.9 if male; α=-0.241 if female and -0.302 if male; min=the minimum of serum creatinine concentration/κ or 1; max=the maximum of serum creatinine concentration/κ or 1.

© 2023 Muanda FT et al. *JAMA Network Open.*

eTable 17. Risk of a hospital visit with myelosuppression, sepsis, pneumotoxicity, and hepatotoxicity in older adults with chronic kidney disease within 90 days of starting a new prescription for low-dose methotrexate at 5 to <15 mg/week vs a new prescription for hydroxychloroquine[a]

	Unmatched		Matched[b]				
	No. events (%)		No. events (%)				
	LD-MTX at 5 to <15 mg/week (n = 1390)	HCQ (n = 4009)	LD-MTX at 5 to <15 mg/week (n = 1212)	HCQ (n = 1212)	Risk difference, % (95% CI)	NNH, % (95% CI)	Risk ratio (95% CI)
**Primary outcome**							
Hospital visit with myelosuppression, sepsis, pneumotoxicity, and hepatotoxicity[c]	37 (2.66)	66 (1.65)	34 (2.81)	22 (1.82)	0.99 (-0.22 to 2.20)	NA	1.55 (0.90 to 2.64)

Abbreviations: LD-MTX, low-dose methotrexate; HCQ, hydroxychloroquine, NA, not applicable
[a] Reference group: hydroxychloroquine
[b] The propensity score was estimated using multivariable logistic regression with 140 covariates chosen *a priori* (defined in eTable 8 in the Supplement). We use greedy matching, to match low-dose methotrexate drug user at 5 to <15 mg/week (1:1) to hydroxychloroquine user based on the logit of the propensity score (within a caliper of ±0.2 standard deviations).[24] Risk ratios and 95% CIs were obtained using modified Poisson regression[26] and weighted risk differences and 95% CIs were obtained using a binomial regression model with an identity link function.
[c] The 90-day risk of a hospital visit with myelosuppression, sepsis, pneumotoxicity, and hepatotoxicity.

© 2023 Muanda FT et al. *JAMA Network Open.*

eTable 18. Baseline characteristics[a] of older adults with chronic kidney disease newly prescribed low-dose methotrexate (LD MTX) at 5 to <15 mg/week vs those newly prescribed hydroxychloroquine (HCQ) in Ontario, Canada (2008–2021)

		Unmatched data (N=5399)					Matched data (N=2424)				
		LD-MTX at 5 to <15 mg/week (n=1390)		HCQ (n=4009)		Standardized difference[c]	LD-MTX at 5 to <15 mg/week (n=1212)		HCQ (n=1212)		Standardized difference[c]
**Demographics**											
Age at cohort entry	Mean ± SD	78.2	7.1	76.9	6.8	19%	78.1	7.0	78.0	7.0	1%
	Median (IQR)	78	(73-84)	76	(71-82)	.	78	(73-83)	78	(73-83)	.
	66-<70	186	13.4%	673	16.8%	10%	157	13.0%	166	13.7%	2%
	70-<75	280	20.1%	947	23.6%	8%	244	20.1%	238	19.6%	1%
	75-<80	340	24.5%	982	24.5%	0%	313	25.8%	294	24.3%	3%
	80-<85	284	20.4%	791	19.7%	2%	243	20.0%	275	22.7%	7%
	85-<90	221	15.9%	468	11.7%	12%	190	15.7%	175	14.4%	4%
	90+	79	5.7%	148	3.7%	9%	65	5.4%	64	5.3%	0%
Sex	F	935	67.3%	2958	73.8%	14%	826	68.2%	829	68.4%	0%
	M	455	32.7%	1051	26.2%	14%	386	31.8%	383	31.6%	0%
Year of cohort entry	2008	41	2.9%	101	2.5%	2%	39	3.2%	47	3.9%	4%
	2009	109	7.8%	238	5.9%	8%	96	7.9%	100	8.3%	1%
	2010	130	9.4%	251	6.3%	12%	110	9.1%	113	9.3%	1%
	2011	121	8.7%	276	6.9%	7%	109	9.0%	105	8.7%	1%
	2012	119	8.6%	246	6.1%	10%	102	8.4%	113	9.3%	3%
	2013	118	8.5%	323	8.1%	1%	101	8.3%	109	9.0%	2%
	2014	107	7.7%	298	7.4%	1%	93	7.7%	88	7.3%	2%
	2015	92	6.6%	308	7.7%	4%	78	6.4%	73	6.0%	2%
	2016	95	6.8%	275	6.9%	0%	81	6.7%	76	6.3%	2%
	2017	83	6.0%	331	8.3%	9%	69	5.7%	70	5.8%	0%
	2018	111	8.0%	375	9.4%	5%	98	8.1%	89	7.3%	3%
	2019	112	8.1%	377	9.4%	5%	96	7.9%	90	7.4%	2%

© 2023 Muanda FT et al. *JAMA Network Open.*

		Unmatched data (N=5399)					Matched data (N=2424)				
		LD-MTX at 5 to <15 mg/week (n=1390)		HCQ (n=4009)		Standardized difference[c]	LD-MTX at 5 to <15 mg/week (n=1212)		HCQ (n=1212)		Standardized difference[c]
	2020	79	5.7%	355	8.9%	12%	72	5.9%	69	5.7%	1%
	2021	73	5.3%	255	6.4%	5%	68	5.6%	70	5.8%	1%
Location	N	1203	86.5%	3513	87.6%	3%	1051	86.7%	1055	87.0%	1%
	Y	187	13.5%	496	12.4%	3%	161	13.3%	157	13.0%	1%
Residence	1	24	1.7%	37	0.9%	7%	19	1.6%	25	2.1%	4%
LHIN	1	72	5.2%	164	4.1%	5%	61	5.0%	54	4.5%	2%
	2	115	8.3%	267	6.7%	6%	100	8.3%	93	7.7%	2%
	3	49	3.5%	232	5.8%	11%	46	3.8%	39	3.2%	3%
	4	223	16.0%	871	21.7%	15%	208	17.2%	211	17.4%	1%
	5	81	5.8%	212	5.3%	2%	76	6.3%	84	6.9%	2%
	6	67	4.8%	239	6.0%	5%	61	5.0%	56	4.6%	2%
	7	74	5.3%	205	5.1%	1%	63	5.2%	67	5.5%	1%
	8	125	9.0%	426	10.6%	5%	111	9.2%	119	9.8%	2%
	9	155	11.2%	394	9.8%	5%	136	11.2%	146	12.0%	2%
	10	80	5.8%	151	3.8%	9%	60	5.0%	56	4.6%	2%
	11	218	15.7%	442	11.0%	14%	176	14.5%	171	14.1%	1%
	12	39	2.8%	180	4.5%	9%	37	3.1%	36	3.0%	1%
	13	68	4.9%	169	4.2%	3%	58	4.8%	57	4.7%	0%
	14	24	1.7%	57	1.4%	2%	19	1.6%	23	1.9%	2%
Socio-economic status[d]	1	285	20.5%	781	19.5%	3%	246	20.3%	222	18.3%	5%
	2	302	21.7%	874	21.8%	0%	259	21.4%	273	22.5%	3%
	3	299	21.5%	835	20.8%	2%	267	22.0%	261	21.5%	1%
	4	258	18.6%	780	19.5%	2%	220	18.2%	243	20.0%	5%
	5	246	17.7%	739	18.4%	2%	220	18.2%	213	17.6%	2%

© 2023 Muanda FT et al. *JAMA Network Open.*

	Unmatched data (N=5399)					Matched data (N=2424)				
	LD-MTX at 5 to <15 mg/week (n=1390)		HCQ (n=4009)		Standardized difference[c]	LD-MTX at 5 to <15 mg/week (n=1212)		HCQ (n=1212)		Standardized difference[c]
**Prescriber information**										
Rheumatologist	627	45.1%	2471	61.6%	34%	604	49.8%	594	49.0%	2%
General practitioner	237	17.1%	619	15.4%	5%	217	17.9%	224	18.5%	2%
Internist	111	8.0%	296	7.4%	2%	105	8.7%	108	8.9%	1%
Dermatologist	193	13.9%	116	2.9%	40%	85	7.0%	85	7.0%	0%
Other	94	6.8%	180	4.5%	10%	85	7.0%	85	7.0%	0%
Missing	128	9.2%	327	8.2%	4%	121	10.0%	126	10.4%	1%
**Comorbidities[e]**										
Acute kidney injury	116	8.3%	249	6.2%	8%	104	8.6%	98	8.1%	2%
Alcoholism	13	0.9%	45	1.1%	2%	11	0.9%	13	1.1%	2%
Angina	229	16.5%	686	17.1%	2%	195	16.1%	210	17.3%	3%
Atrial fibrillation/flutter	105	7.6%	280	7.0%	2%	94	7.8%	115	9.5%	6%
Bipolar disorder	21	1.5%	83	2.1%	5%	18	1.5%	17	1.4%	1%
Chronic liver disease	58	4.2%	201	5.0%	4%	50	4.1%	54	4.5%	2%
Chronic obstructive pulmonary disease	402	28.9%	1203	30.0%	2%	353	29.1%	358	29.5%	1%
Cirrhosis	40	2.9%	141	3.5%	3%	35	2.9%	39	3.2%	2%
Coronary artery disease (minus angina)	397	28.6%	1105	27.6%	2%	348	28.7%	342	28.2%	1%
Dementia	130	9.4%	329	8.2%	4%	109	9.0%	119	9.8%	3%
Anemia	416	29.9%	1201	30.0%	0%	371	30.6%	373	30.8%	0%
Glaucoma	148	10.6%	476	11.9%	4%	125	10.3%	127	10.5%	1%
Major hemorrhage	84	6.0%	235	5.9%	0%	71	5.9%	84	6.9%	4%

© 2023 Muanda FT et al. *JAMA Network Open.*

	Unmatched data (N=5399)					Matched data (N=2424)				
	LD-MTX at 5 to <15 mg/week (n=1390)		HCQ (n=4009)		Standardized difference[c]	LD-MTX at 5 to <15 mg/week (n=1212)		HCQ (n=1212)		Standardized difference[c]
Congestive heart failure	243	17.5%	668	16.7%	2%	217	17.9%	222	18.3%	1%
Hypertension	1116	80.3%	3303	82.4%	5%	978	80.7%	991	81.8%	3%
Hypokalemia	35	2.5%	76	1.9%	4%	30	2.5%	27	2.2%	2%
Hyponatremia	33	2.4%	77	1.9%	3%	27	2.2%	29	2.4%	1%
Hypothyroidism	200	14.4%	574	14.3%	0%	174	14.4%	193	15.9%	4%
Migraine	47	3.4%	153	3.8%	2%	35	2.9%	41	3.4%	3%
Obesity	75	5.4%	256	6.4%	4%	64	5.3%	61	5.0%	1%
Parkinson disease	28	2.0%	47	1.2%	6%	22	1.8%	23	1.9%	1%
Peripheral vascular disease	18	1.3%	47	1.2%	1%	16	1.3%	12	1.0%	3%
Schizophrenia	29	2.1%	67	1.7%	3%	27	2.2%	17	1.4%	6%
Ischaemic stroke	25	1.8%	51	1.3%	4%	20	1.7%	23	1.9%	2%
Depression	110	7.9%	364	9.1%	4%	96	7.9%	108	8.9%	4%
Rheumatoid arthritis	697	50.1%	1922	47.9%	4%	660	54.5%	675	55.7%	2%
Syncope	22	1.6%	44	1.1%	4%	16	1.3%	14	1.2%	1%
Arrhythmia	141	10.1%	392	9.8%	1%	127	10.5%	146	12.0%	5%
Inflammatory bowel disease	26	1.9%	33	0.8%	10%	22	1.8%	17	1.4%	3%
Major cancer[f]	560	40.3%	1617	40.3%	0%	493	40.7%	479	39.5%	2%
Prostatic hyperplasia	129	9.3%	364	9.1%	1%	112	9.2%	108	8.9%	1%
Fracture	111	8.0%	313	7.8%	1%	92	7.6%	100	8.3%	3%
Falls	86	6.2%	187	4.7%	7%	69	5.7%	75	6.2%	2%
Hyperkaliema	15	1.1%	46	1.1%	0%	13	1.1%	22	1.8%	6%
Prostatitis	27	1.9%	75	1.9%	0%	26	2.1%	22	1.8%	2%
Hypotension	34	2.4%	65	1.6%	6%	29	2.4%	29	2.4%	0%

© 2023 Muanda FT et al. *JAMA Network Open.*

	Unmatched data (N=5399)					Matched data (N=2424)				
	LD-MTX at 5 to <15 mg/week (n=1390)		HCQ (n=4009)		Standardized difference[c]	LD-MTX at 5 to <15 mg/week (n=1212)		HCQ (n=1212)		Standardized difference[c]
Gallstones /biliary stones	70	5.0%	197	4.9%	0%	62	5.1%	60	5.0%	0%
Prior pneumotoxcity	73	5.3%	173	4.3%	5%	65	5.4%	56	4.6%	4%
Prior myelotoxicity	21	1.5%	41	1.0%	5%	18	1.5%	16	1.3%	2%
Prior sepsis	25	1.8%	58	1.4%	3%	25	2.1%	29	2.4%	2%
Prior methotrexate toxicity	106	7.6%	250	6.2%	6%	95	7.8%	89	7.3%	2%
Psoriasis	230	16.5%	130	3.2%	46%	105	8.7%	103	8.5%	1%
Dermatomyositis	106	7.6%	546	13.6%	20%	103	8.5%	94	7.8%	3%
Sarcoidosis	17	1.2%	24	0.6%	6%	14	1.2%	12	1.0%	2%
Systemic sclerosis or scleroderma	112	8.1%	305	7.6%	2%	96	7.9%	100	8.3%	1%
Systemic lupus erythematosus	141	10.1%	664	16.6%	19%	130	10.7%	122	10.1%	2%
Atopic dermatitis or eczema	482	34.7%	1154	28.8%	13%	376	31.0%	373	30.8%	0%
Ulcerative colitis (UC)	32	2.3%	56	1.4%	7%	29	2.4%	26	2.1%	2%
Crohn disease	27	1.9%	36	0.9%	9%	23	1.9%	19	1.6%	2%
Acute urinary retention	34	2.4%	71	1.8%	4%	27	2.2%	25	2.1%	1%
Myocardial infarction	46	3.3%	122	3.0%	2%	43	3.5%	39	3.2%	2%
Dyslipidemia	369	26.5%	1009	25.2%	3%	314	25.9%	309	25.5%	1%
Macula degeneration	78	5.6%	132	3.3%	11%	58	4.8%	62	5.1%	1%
Gastroesophageal reflux disease	355	25.5%	1101	27.5%	5%	304	25.1%	321	26.5%	3%
Osteoarthritis	131	9.4%	475	11.8%	8%	119	9.8%	142	11.7%	6%
Major surgery	85	6.1%	229	5.7%	2%	75	6.2%	72	5.9%	1%

© 2023 Muanda FT et al. *JAMA Network Open.*

		Unmatched data (N=5399)					Matched data (N=2424)				
		LD-MTX at 5 to <15 mg/week (n=1390)		HCQ (n=4009)		Standardized difference[c]	LD-MTX at 5 to <15 mg/week (n=1212)		HCQ (n=1212)		Standardized difference[c]
Prostate cancer		51	3.7%	115	2.9%	4%	46	3.8%	39	3.2%	3%
Diabete		339	24.4%	990	24.7%	1%	294	24.3%	297	24.5%	0%
Urinary tract infection		122	8.8%	262	6.5%	9%	103	8.5%	108	8.9%	1%
Gout		203	14.6%	610	15.2%	2%	174	14.4%	165	13.6%	2%
Modified Charlson comorbidity index[g]	Mean ± SD	2.58	1.3	2.45	1.13	11%	2.58	1.28	2.56	1.25	2%
	Median (IQR)	2	(2-2)	2	(2-2)	.	2	(2-2)	2	(2-2)	.
	2	1065	76.6%	3218	80.3%	9%	928	76.6%	929	76.7%	0%
	3+	325	23.4%	791	19.7%	9%	284	23.4%	283	23.3%	0%
**Medication use[h]**											
Alpha-adrenergic blocking agents		47	3.4%	133	3.3%	1%	42	3.5%	42	3.5%	0%
Anti-arrhythmic		14	1.0%	39	1.0%	0%	14	1.2%	18	1.5%	3%
Antibiotics		404	29.1%	1127	28.1%	2%	344	28.4%	334	27.6%	2%
Ace inhibitor		390	28.1%	1183	29.5%	3%	341	28.1%	345	28.5%	1%
Anticoagulants		156	11.2%	504	12.6%	4%	141	11.6%	155	12.8%	4%
Anticonvulsants		111	8.0%	405	10.1%	7%	101	8.3%	82	6.8%	6%
Angiotensin II receptor blockers		441	31.7%	1379	34.4%	6%	391	32.3%	402	33.2%	2%
Aspirin		39	2.8%	91	2.3%	3%	33	2.7%	35	2.9%	1%
Antiplatelet agents		77	5.5%	275	6.9%	6%	71	5.9%	67	5.5%	2%
Antifungals		31	2.2%	73	1.8%	3%	25	2.1%	30	2.5%	3%
Tricyclic antidepressant		191	13.7%	676	16.9%	9%	163	13.4%	155	12.8%	2%
Gastrointestinal drugs		86	6.2%	191	4.8%	6%	75	6.2%	67	5.5%	3%

© 2023 Muanda FT et al. *JAMA Network Open.*

	Unmatched data (N=5399)					Matched data (N=2424)				
	LD-MTX at 5 to <15 mg/week (n=1390)		HCQ (n=4009)		Standardized difference[c]	LD-MTX at 5 to <15 mg/week (n=1212)		HCQ (n=1212)		Standardized difference[c]
Beta-blockers	450	32.4%	1363	34.0%	3%	396	32.7%	407	33.6%	2%
Bone Calcium regulators	29	2.1%	98	2.4%	2%	26	2.1%	20	1.7%	3%
Benzodiazepine	187	13.5%	569	14.2%	2%	160	13.2%	154	12.7%	1%
Bisphosphonates	280	20.1%	716	17.9%	6%	251	20.7%	237	19.6%	3%
Beta agonist	140	10.1%	400	10.0%	0%	120	9.9%	123	10.1%	1%
H2 blockers	53	3.8%	165	4.1%	2%	48	4.0%	49	4.0%	0%
Channel calcium blockers	461	33.2%	1419	35.4%	5%	413	34.1%	412	34.0%	0%
Cholinesterase inhibitors	21	1.5%	54	1.3%	2%	15	1.2%	10	0.8%	4%
Glucocorticoid[i]	854	61.4%	1998	49.8%	24%	715	59.0%	706	58.3%	1%
Loop diuretics	271	19.5%	714	17.8%	4%	230	19.0%	249	20.5%	4%
Nitrates	72	5.2%	208	5.2%	0%	62	5.1%	60	5.0%	0%
Fibrates	26	1.9%	85	2.1%	1%	20	1.7%	23	1.9%	2%
NSAIDs (excluding ASA)	277	19.9%	965	24.1%	10%	256	21.1%	251	20.7%	1%
Insulin	124	8.9%	340	8.5%	1%	107	8.8%	109	9.0%	1%
Opioids	317	22.8%	1002	25.0%	5%	285	23.5%	292	24.1%	1%
Over-active bladder medication	33	2.4%	149	3.7%	8%	30	2.5%	39	3.2%	4%
Potassium Sparing diuretics	88	6.3%	276	6.9%	2%	76	6.3%	77	6.4%	0%
Allopurinol	138	9.9%	450	11.2%	4%	123	10.1%	115	9.5%	2%
Anti-psychotics	39	2.8%	91	2.3%	3%	31	2.6%	30	2.5%	1%
Proton pump inhibitors	604	43.5%	1863	46.5%	6%	532	43.9%	544	44.9%	2%

© 2023 Muanda FT et al. *JAMA Network Open.*

		Unmatched data (N=5399)					Matched data (N=2424)				
		LD-MTX at 5 to <15 mg/week (n=1390)		HCQ (n=4009)		Standardized difference[c]	LD-MTX at 5 to <15 mg/week (n=1212)		HCQ (n=1212)		Standardized difference[c]
5 alpha-reductase		72	5.2%	228	5.7%	2%	65	5.4%	59	4.9%	2%
Selective serotonin reuptake inhibitors		148	10.6%	478	11.9%	4%	127	10.5%	143	11.8%	4%
Statins		689	49.6%	2128	53.1%	7%	604	49.8%	597	49.3%	1%
Thiazide diuretics		265	19.1%	736	18.4%	2%	227	18.7%	238	19.6%	2%
Oral antidiabetics		280	20.1%	847	21.1%	2%	242	20.0%	238	19.6%	1%
Number of unique drug names	Mean ± SD	8.39	4.52	8.24	4.38	3%	8.29	4.43	8.33	4.49	1%
	Median (IQR)	8	(5-11)	8	(5-11)	.	8	(5-11)	8	(5-11)	.
	0-4	245	17.6%	776	19.4%	5%	218	18.0%	232	19.1%	3%
	5-9	649	46.7%	1828	45.6%	2%	570	47.0%	551	45.5%	3%
	10-14	377	27.1%	1073	26.8%	1%	327	27.0%	324	26.7%	1%
	15-19	83	6.0%	263	6.6%	2%	71	5.9%	77	6.4%	2%
	20+	36	2.6%	69	1.7%	6%	26	2.1%	28	2.3%	1%
Number of unique dins	Mean ± SD	8.98	5.07	8.82	4.92	3%	8.87	4.98	8.94	5.1	1%
	Median (IQR)	8	(6-12)	8	(5-11)	.	8	(6-12)	8	(5-12)	.
	0-4	228	16.4%	725	18.1%	5%	204	16.8%	216	17.8%	3%
	5-9	597	42.9%	1698	42.4%	1%	525	43.3%	516	42.6%	1%
	10-14	404	29.1%	1121	28.0%	2%	348	28.7%	327	27.0%	4%
	15-19	108	7.8%	326	8.1%	1%	94	7.8%	108	8.9%	4%
	20+	53	3.8%	139	3.5%	2%	41	3.4%	45	3.7%	2%
**Healthcare use[j]**											
Primary care visits	Mean ± SD	10.63	9.54	10.3	9.84	3%	10.51	9.5	10.97	10.95	4%
	Median (IQR)	8	(5-13)	8	(5-13)	.	8	(5-13)	8	(5-14)	.
	0-4	296	21.3%	903	22.5%	3%	264	21.8%	252	20.8%	2%
	5-9	509	36.6%	1474	36.8%	0%	457	37.7%	430	35.5%	5%

© 2023 Muanda FT et al. *JAMA Network Open.*

		Unmatched data (N=5399)					Matched data (N=2424)				
		LD-MTX at 5 to <15 mg/week (n=1390)		HCQ (n=4009)		Standardized difference[c]	LD-MTX at 5 to <15 mg/week (n=1212)		HCQ (n=1212)		Standardized difference[c]
	10-14	292	21.0%	844	21.1%	0%	237	19.6%	261	21.5%	5%
	15-19	132	9.5%	394	9.8%	1%	112	9.2%	124	10.2%	3%
	20+	161	11.6%	394	9.8%	6%	142	11.7%	145	12.0%	1%
Hospitalizations	Mean ± SD	0.22	0.59	0.16	0.54	11%	0.21	0.57	0.23	0.66	3%
	Median (IQR)	0	(0-0)	0	(0-0)	.	0	(0-0)	0	(0-0)	.
	0	1174	84.5%	3550	88.6%	12%	1028	84.8%	1028	84.8%	0%
	1	157	11.3%	331	8.3%	10%	136	11.2%	123	10.1%	4%
	2	39	2.8%	93	2.3%	3%	32	2.6%	42	3.5%	5%
	3+	20	1.4%	35	0.9%	5%	16	1.3%	19	1.6%	3%
Emergency departments visits	Mean ± SD	0.82	1.46	0.7	1.4	8%	0.8	1.44	0.77	1.37	2%
	Median (IQR)	0	(0-1)	0	(0-1)	.	0	(0-1)	0	(0-1)	.
	0	834	60.0%	2593	64.7%	10%	730	60.2%	732	60.4%	0%
	1	289	20.8%	782	19.5%	3%	259	21.4%	267	22.0%	1%
	2	133	9.6%	327	8.2%	5%	112	9.2%	109	9.0%	1%
	3+	134	9.6%	307	7.7%	7%	111	9.2%	104	8.6%	2%
TSH test		961	69.1%	2998	74.8%	13%	862	71.1%	871	71.9%	2%
At-home physician service		41	2.9%	89	2.2%	4%	35	2.9%	27	2.2%	4%
Bone mineral density test		195	14.0%	657	16.4%	7%	170	14.0%	180	<15%	3%
Cardiac catheterization		17	1.2%	48	1.2%	0%	15	1.2%	19	1.6%	3%
Cardiac stress test		191	13.7%	566	14.1%	1%	168	13.9%	170	14.0%	0%
Carotid ultrasound		76	5.5%	193	4.8%	3%	67	5.5%	64	5.3%	1%
Chest X-ray		675	48.6%	1694	42.3%	13%	577	47.6%	578	47.7%	0%
Cataract surgery		79	5.7%	205	5.1%	3%	68	5.6%	71	5.9%	1%

© 2023 Muanda FT et al. *JAMA Network Open.*

	Unmatched data (N=5399)					Matched data (N=2424)				
	LD-MTX at 5 to <15 mg/week (n=1390)		HCQ (n=4009)		Standardized difference[c]	LD-MTX at 5 to <15 mg/week (n=1212)		HCQ (n=1212)		Standardized difference[c]
Cervical cancer screening	44	3.2%	130	3.2%	0%	34	2.8%	42	3.5%	4%
Colorectal cancer screening	211	15.2%	607	15.1%	0%	185	15.3%	198	16.3%	3%
Cholesterol test (total cholesterol, HDL)	896	64.5%	2663	66.4%	4%	778	64.2%	773	63.8%	1%
CT abdomen	164	11.8%	387	9.7%	7%	142	11.7%	145	12.0%	1%
CT extremities	9	0.6%	60	1.5%	9%	8	0.7%	6	0.5%	3%
CT head	138	9.9%	355	8.9%	3%	116	9.6%	131	10.8%	4%
CT neck	21	1.5%	42	1.0%	5%	15	1.2%	17	1.4%	2%
CT pelvis	155	11.2%	361	9.0%	7%	135	11.1%	136	11.2%	0%
CT spine	23	1.7%	104	2.6%	6%	21	1.7%	28	2.3%	4%
CT thorax	184	13.2%	464	11.6%	5%	161	13.3%	154	12.7%	2%
Echocardiography	359	25.8%	1046	26.1%	1%	311	25.7%	304	25.1%	1%
Flu shot	735	52.9%	1951	48.7%	8%	632	52.1%	643	53.1%	2%
Cystoscopy	67	4.8%	172	4.3%	2%	59	4.9%	63	5.2%	1%
Hearing test	60	4.3%	210	5.2%	4%	54	4.5%	55	4.5%	0%
Mammography	131	9.4%	595	14.8%	17%	118	9.7%	113	9.3%	1%
Prostate-specific antigen (psa) test	15	1.1%	36	0.9%	2%	13	1.1%	19	1.6%	4%
Holter monitoring	121	8.7%	361	9.0%	1%	104	8.6%	103	8.5%	0%
Parathyroid hormone testing	154	11.1%	686	17.1%	17%	143	11.8%	148	12.2%	1%
Pulmonary function test	178	12.8%	544	13.6%	2%	163	13.4%	152	12.5%	3%
Urinalysis	804	57.8%	2489	62.1%	9%	704	58.1%	714	58.9%	2%
**Laboratory measurement**										

© 2023 Muanda FT et al. *JAMA Network Open.*

		Unmatched data (N=5399)					Matched data (N=2424)				
		LD-MTX at 5 to <15 mg/week (n=1390)		HCQ (n=4009)		Standardized difference[c]	LD-MTX at 5 to <15 mg/week (n=1212)		HCQ (n=1212)		Standardized difference[c]
eGFR[k]	Mean ± SD	47.42	9.72	47.54	9.87	1%	47.41	9.75	47.27	10.17	1%
	Median (IQR)	49	(41-55)	50	(41-56)	.	49	(41-55)	49	(41-56)	.
Baseline eGFR categories	45-<60	903	65.0%	2643	65.9%	2%	788	65.0%	778	64.2%	2%
	30-<45	404	29.1%	1100	27.4%	4%	350	28.9%	345	28.5%	1%
	<30	83	6.0%	266	6.6%	2%	74	6.1%	89	7.3%	5%
Urine ACR available		495	35.6%	1597	39.8%	9%	440	36.3%	436	36.0%	1%
	Missing	895	64.4%	2412	60.2%	9%	772	63.7%	776	64.0%	1%
Baseline ACR categories, µg/mg	0-<3	293	21.1%	994	24.8%	9%	258	21.3%	263	21.7%	1%
	3-30	151	10.9%	433	10.8%	0%	138	11.4%	132	10.9%	2%
	>30	51	3.7%	170	4.2%	3%	44	3.6%	41	3.4%	1%

Abbreviations: LD-MTX, low-dose methotrexate; HCQ, hydroxychloroquine; ACE inhibitor, angiotensin-converting-enzyme inhibitor; H2 blockers, Histamine H2-receptor antagonists eGFR, estimated glomerular filtration rate; IQR, interquartile range; LHIN, Local Health Integration Network; ACR, urine albumin-to-creatinine ratio.[a] Unless otherwise specified in the footnotes, baseline characteristics were assessed on the date the patient filled a low-dose methotrexate prescription or a hydroxychloroquine prescription—the cohort entry date.

[b] Propensity score matching technique was used to balance comparison groups on indicators of baseline health, including all known indications for methotrexate use (including off-label indications). The propensity score was estimated using multivariable logistic regression with 140 covariates chosen *a priori* (defined in eTable 8 in the Supplement. We use greedy matching to match low-dose methotrexate drug user (1:1) to hydroxychloroquine user based on the logit of the propensity score (within a caliper of ±0.2 standard deviations).[24]

[c] The difference between the groups divided by the pooled SD; a value greater than 10% is interpreted as a meaningful difference.[25]

[d] Income was categorized into fifths of average neighborhood income on the cohort entry date.

[e] Baseline comorbidities were assessed in the 5-year period before the cohort entry date.

[f] Cancer includes the following types of cancer: skin, mouth (lip, tonsil, etc), throat, stomach, small/large intestine, liver, gall bladder, pancreas, breast, male/female reproductive organs, heart, lung, bone, urinary system (kidney, bladder, etc), endocrine glands, as well as leukemias and lymphomas

[g] Presence of kidney disease is a variable in the Charlson comorbidity index, which automatically results in all individuals receiving a minimum score of 2

[h] Medication use was examined in the 120-day period before the cohort entry date (the Ontario Drug Benefit program dispenses a maximum 100-day supply).

[I] Glucocorticoids included many medications regardless of their route of administration such as hydrocortisone acetate, dexamethasone, beclomethasone dipropionate, prednisone, hydrocortisone, flumetasone pivalate, clioquinol, betamethasone valerate, betamethasone, triamcinolone acetonide, triamcinolone

© 2023 Muanda FT et al. *JAMA Network Open.*

diacetate, triamcinolone, flurandrenolide, betamethasone & dexamethasone sodium phosphate, cortisone acetate, dexamethasone tebutate, prednisolone, dexamethasone, corticotrophin, prednisolone acetate, fluocinolone acetonide, hydrocortisone sodium succinate, methylprednisolone sodium succinate, methylprednisolone acetate, methylprednisolone disodium phosphate, methylprednisolone, fluocinonide, betamethasone disodium phosphate, medrysone & polyvinyl alcohol, prednisolone acetate & sulfacetamide sodium, dexamethasone & neomycin sulfate & polymyxin b sulfate, clioquinol & flumetasone pivalate, clioquinol & hydrocortisone, 1,2-propanediol diacetate & acetic acid & benzethonium chloride & hydrocortisone, clioquinol & triamcinolone acetonide, flurandrenolide, fluocinolone acetonide, dexamethasone & neomycin sulfate, hydrocortisone & lidocaine hcl & neomycin sulfate, haemorroidal veinous plexus, prednisone & pheniramine maleate & inositol & phosphatidyl choline & vitamin a & vitamin d2& vitamin e, chloramphenicol & hydrocortisone acetate, haemorroidal veinous plexus, dexamethasone & framycetin sulfate & gramicidin, dibucaine hcl & esculin & framycetin sulfate & hydrocortisone, betamethasone valerate & neomycin sulfate, betamethasone valerate & gentamicin sulfate, prednisolone acetate & sulfacetamide sodium, ascorbic acid & chlorpheniramine maleate & prednisone acetate, neomycin sulfate & prednisolone acetate & sulfacetamide sodium, gramicidin & neomycin sulfate & triamcinolone acetonide, methylprednisolone, acetylsalicylic acid & methyltestosterone, methylprednisolone sulfate & neomycin sulfate, hydrocortisone acetate & neomycin sulfate, aluminum chlorohydrate & methylprednisolone acetate & neomycin sulfate & sulfur, gramicidin & neomycin sulfate & nystatin & triamcinolone acetonide, hydrocortisone acetate & zinc oxide, hydrocortisone acetate & pramoxine hcl & zinc sulfate, aluminum chlorohydrate & methylprednisolone acetate & sulfur, hydrocortisone acetate & zinc oxide, hydrocortisone acetate & pramoxine hcl & zinc sulfate, desonide, clobetasol propionate, beclomethasone dipropionate & clioquinol, bacitracin zinc & hydrocortisone & neomycin sulfate & polymyxin b sulfate, hydrocortisone & neomycin sulfate & polymyxin b sulfate, gramicidin & neomycin sulfate & nystatin & triamcinolone acetonide, fluorometholone & polyvinyl alcohol, aluminum chlorohydrate & methylprednisolone acetate & sulfur, fluorometholone, lidocaine hcl & methylprednisolone acetate, flumetasone pivalate & salicylic acid, fluorometholone, lidocaine hcl & methylprednisolone acetate, aclometasone dipropionate, allantoin & chloramphenicol & hydrocortisone, amcinonide, atropine sulfate & prednisolone acetate, bacitracin & hydrocortisone & neomycin sulfate & polymyxin b sulfate, benzalkonium & dexamethasone & tobramycin , benzocaine & hydrocortisone acetate & zinc sulfate, betamethasone & sulfacetamide sodium, betamethasone acetate & betamethasone sodium phosphate, betamethasone benzoate, betamethasone dipropionate, betamethasone dipropionate &calcipotriene, betamethasone dipropionate &clotrimazole, betamethasone dipropionate & gentamicin sulfate, betamethasone dipropionate & salicylic acid, betamethasone disodium phosphate, betamethasone valerate & salicylic acid, betamethasone valerate & gentamycin sulfate, betamethasone valerate & neomycin sulfate, budesonide, camphor & hydrocortisone & menthol, chlorbutol & dexamethasone &tobramycin, ciclesonide.

[j] Total number of healthcare visits/tests in the 12-month period before the cohort entry date.

[k] The most recent eGFR measurement in the 365-day period before the cohort entry date (including the cohort entry date); eGFR was calculated using the new Chronic Kidney Disease (CKD)–Epidemiology (EPI) equation: $142\times \min([\text{serum creatinine concentration in } \mu mol/L/88.4]/\kappa, 1)^{\alpha} \times \max([\text{serum creatinine concentration in } \mu mol/L/88.4]/\kappa, 1)^{-1.200} \times 0.9938^{Age} \times 1.012$ [if female]; κ=0.7 if female and 0.9 if male; α=-0.241 if female and -0.302 if male; min=the minimum of serum creatinine concentration/κ or 1; max=the maximum of serum creatinine concentration/κ or 1.

© 2023 Muanda FT et al. *JAMA Network Open.*

eTable 19. Survival analysis in older adults with chronic kidney disease who started a new prescription for low-dose methotrexate- vs a new prescription for hydroxychloroquine[a]: risk of a hospital visit with myelosuppression, sepsis, pneumotoxicity, and hepatotoxicity

	**Unmatched**				**Matched[b]**				
	**No. events (%)**		**No. events per 1000 person-years**		**No. events (%)**		**No. events per 1000 person-years**		
	**LD-MTX (n = 2900)**	**HCQ (n = 4009)**	**LD-MTX**	**HCQ**	**LD-MTX (n = 2309)**	**HCQ (n = 2309)**	**LD-MTX**	**HCQ**	**Hazard ratio (95% CI)**
Hospital visit with myelosuppression, sepsis, pneumotoxicity, and hepatotoxicity[c] (death censored)	97 (3.34)	66 (1.65)	138.7	67.6	82 (3.55)	40 (1.73)	147.1	71.1	2.03 (1.19 to 3.44)

Abbreviations: LD-MTX, low-dose methotrexate; HCQ, hydroxychloroquine

[a] Reference group: hydroxychloroquine

[b] The propensity score was estimated using multivariable logistic regression with 140 covariates chosen *a priori* (defined in eTable 8 in the Supplement).We use greedy matching to match low-dose methotrexate drug user (1:1) to hydroxychloroquine user based on the logit of the propensity score (within a caliper of ±0.2 standard deviations).[24] Hazard ratios and 95% confidence intervals were obtained using a Cox proportional hazards regression, and 95% confidence intervals were obtained using a bootstrap variance estimator. The proportional hazards assumption was assessed using a time-dependent covariate test and was met for the primary outcome. Death was treated as a censoring event.

[c] The 90-day risk of a hospital visit with myelosuppression, sepsis, pneumotoxicity, and hepatotoxicity

© 2023 Muanda FT et al. *JAMA Network Open.*

eTable 20. Risk of a hospital visit with myelosuppression, sepsis, pneumotoxicity, and hepatotoxicity in older adults with chronic kidney disease within 90 days of starting a new prescription for low-dose methotrexate vs. a new prescription for hydroxychloroquine[a] using Inverse probability of treatment weighting analysis

**Outcome**	**Unweighted**		**Weighted[b]**		**Risk difference, % (95% CI)**	**Number needed to harm (95% CI)**	**Risk ratio (95% CI)**
	**No. events (%)**		**No. events (%)**				
	**LD-MTX**	**HCQ**	**LD-MTX**	**HCQ**			
	**(n = 2900)**	**(n = 4009)**	**(n = 2900)**	**(n = 2921)**			
Hospital visit with myelosuppression, sepsis, pneumotoxicity, and hepatotoxicity	97 (3.34)	66 (1.65)	97 (3.34)	51 (1.75)	1.60 (0.74 to 2.46)	63 (41 to 135)	1.91 (1.32 to 2.79)

Abbreviations: LD-MTX, low-dose methotrexate; HCQ, hydroxychloroquine.

[a] Reference group: hydroxychloroquine.

[b]The propensity score was estimated using multivariable logistic regression with 140 covariates chosen *a priori* (defined in eTable 8 in the Supplement).[27-29] Patients in the reference group were weighted as [propensity score/(1 – propensity score)]. [27-29] This method produces a weighted pseudo-sample of patients in the reference group with the same distribution of measured covariates as the exposed group.[27,28] Weighted risk ratios and 95% CIs were obtained using modified Poisson regression[26]and weighted risk differences and 95% CIs were obtained using a binomial regression model with an identity link function.

© 2023 Muanda FT et al. *JAMA Network Open.*

eTable 21. Risk of a hospital visit with myelosuppression, sepsis, pneumotoxicity, and hepatotoxicity in older adults with chronic kidney disease within 90 days of starting a new prescription for low-dose methotrexate vs a new prescription for hydroxychloroquine[a] using Fine stratification weighting[b]

Outcome	Unweighted No. events (%)		Weighted[b] No. events (%)		Risk difference, % (95% CI)	Number needed to harm (95% CI)	Risk ratio (95% CI)
	LD-MTX (n = 2900)	HCQ (n = 4009)	LD-MTX (n = 1690)	HCQ (n = 2479)			
Hospital visit with myelosuppression, sepsis, pneumotoxicity, and hepatotoxicity	97 (3.34)	66 (1.65)	53 (3.14)	42 (1.69)	1.44 (0.23 to 2.64)	69 (38 to 435)	1.84 (1.03 to 3.28)

Abbreviations: LD-MTX, low-dose methotrexate; HCQ, hydroxychloroquine

[a] Reference group: hydroxychloroquine

[b] This weighting method does not use the propensity score directly to calculate weights; instead, propensity scores are used to create fine stratum after ranking only the exposed patients (LD-MTX users) based on the propensity score and assigning unexposed patients (HCQ users) to these strata based on their propensity score. The weights for the exposed group are set to 1 and reference patients are re-weighted based on the number of exposed patients residing within their stratum, so that unexposed patients contribute proportionally to the relative number of total patients within a stratum. Patients in the unexposed group are weighted as ($N_{\text{exposed in PS stratum i}}/N_{\text{total exposed}}$) / ($N_{\text{unexposed in PS stratum i}}/N_{\text{total unexposed}}$). This weighting creates a pseudo-population in which confounder distribution concordance is achieved between the exposed and unexposed groups, to the extent that it is achieved within each stratum. As a result, extreme weights due to propensity scores that are very close to 0 or 1 are unlikely.[30,31] This method calculates a treatment effect estimate similar to the propensity score matching estimate (i.e., an average treatment effect among the treated population, ATT).[30,31]

.

© 2023 Muanda FT et al. *JAMA Network Open.*

eTable 22. Risk of a hospital visit with myelosuppression, sepsis, pneumotoxicity, and hepatotoxicity in older adults with chronic kidney disease within 90 days of starting a new prescription for low-dose methotrexate at 15 to 35 mg/week vs 5 to <15 mg/week[a]

	**Unweighted**		**Weighted[b]**				
	**No. events (%)**		**No. events (%)**				
	**15 to 35 mg/week (n = 1510)**	**5 to <15 mg/week (n = 1390)**	**15 to 35 mg/week (n = 1510)**	**5 to <15 mg/week (n = 1514)**	**Risk difference, % (95% CI)**	**NNH, % (95% CI)**	**Risk ratio (95% CI)**
**Primary outcome**							
Hospital visit with myelosuppression, sepsis, pneumotoxicity, and hepatotoxicity[c]	60 (3.97)	37 (2.66)	60 (3.97)	28 (1.85)	2.13 (0.85 to 3.40)	47 (29 to 118)	2.15 (1.30 to 3.56)

Abbreviations: LD-MTX, low-dose methotrexate; HCQ, hydroxychloroquine, NA, not applicable
[a] Reference group: LD-MTX at 5 to <15 mg/week
[b]The propensity score was estimated using multivariable logistic regression with 140 covariates chosen *a priori* (defined in eTable 8 in the Supplement).[26-29] Patients in the reference group were weighted as [propensity score/(1 – propensity score)]. [26-29]This method produces a weighted pseudo-sample of patients in the reference group with the same distribution of measured covariates as the exposed group.[26,27] Weighted risk ratios and 95% CIs were obtained using modified Poisson regression[26]and weighted risk differences and 95% CIs were obtained using a binomial regression model with an identity link function

© 2023 Muanda FT et al. *JAMA Network Open.*

eTable 23. Baseline characteristics[a] of older adults with chronic kidney disease newly prescribed low-dose methotrexate (LD MTX) at 15 to 35mg/week vs 5 to<15 mg/week in Ontario, Canada (2008–2021)

		Unweighted data (=2900) LD-MTX					Weighted data (n=3024)[b] LD-MTX				.
		15 to 35 mg/week (n=1510		5 to <15 mg/week (n=1390)		Standardized Difference[c]	15 to 35 mg/week (n=1510)		5 to <15 mg/week (n=1514)		Standardized Difference[c]
**Demographics**											
Age at cohort entry	Mean ± SD	76.3	6.7	78.2	7.1	27%	76.3	6.7	76	7.2	4%
	Median (IQR)	76	(71-81)	78	(73-84)	.	76	(71-81)	75	(70-81)	.
	66-<70	288	19.1%	186	13.4%	15%	288	19.1%	339	22.4%	8%
	70-<75	369	24.4%	280	20.1%	10%	369	24.4%	341	22.5%	4%
	75-<80	363	24.0%	340	24.5%	1%	363	24.0%	370	24.4%	1%
	80-<85	298	19.7%	284	20.4%	2%	298	19.7%	262	17.3%	6%
	85-<90	149	9.9%	221	15.9%	18%	149	9.9%	157	10.3%	1%
	90+	43	2.8%	79	5.7%	14%	43	2.8%	46	3.0%	1%
sex	F	983	65.1%	935	67.3%	5%	983	65.1%	962	63.6%	3%
	M	527	34.9%	455	32.7%	5%	527	34.9%	551	36.4%	3%
Year of cohort entry	2008	40	2.6%	41	2.9%	2%	40	2.6%	40	2.6%	0%
	2009	90	6.0%	109	7.8%	7%	90	6.0%	105	6.9%	4%
	2010	97	6.4%	130	9.4%	11%	97	6.4%	91	6.0%	2%
	2011	109	7.2%	121	8.7%	6%	109	7.2%	109	7.2%	0%
	2012	117	7.7%	119	8.6%	3%	117	7.7%	120	7.9%	1%
	2013	98	6.5%	118	8.5%	8%	98	6.5%	98	6.5%	0%
	2014	104	6.9%	107	7.7%	3%	104	6.9%	111	7.4%	2%
	2015	92	6.1%	92	6.6%	2%	92	6.1%	93	6.1%	0%
	2016	111	7.4%	95	6.8%	2%	111	7.4%	101	6.7%	3%
	2017	135	8.9%	83	6.0%	11%	135	8.9%	131	8.6%	1%

© 2023 Muanda FT et al. *JAMA Network Open.*

		Unweighted data (=2900) LD-MTX					Weighted data (n=3024)[b] LD-MTX				.
		15 to 35 mg/week (n=1510		5 to <15 mg/week (n=1390)		Standardized Difference[c]	15 to 35 mg/week (n=1510)		5 to <15 mg/week (n=1514)		Standardized Difference[c]
	2018	132	8.7%	111	8.0%	3%	132	8.7%	137	9.0%	1%
	2019	138	9.1%	112	8.1%	4%	138	9.1%	141	9.3%	1%
	2020	135	8.9%	79	5.7%	12%	135	8.9%	136	9.0%	0%
	2021	112	7.4%	73	5.3%	9%	112	7.4%	101	6.7%	3%
Location	Urban	1314	87.0%	1203	86.5%	1%	1314	87.0%	1319	87.2%	1%
	rural	196	13.0%	187	13.5%	1%	196	13.0%	194	12.8%	1%
Residence	1	18	1.2%	24	1.7%	4%	18	1.2%	18	1.2%	0%
LHIN	1	87	5.8%	72	5.2%	3%	87	5.8%	83	5.5%	1%
	2	123	8.1%	115	8.3%	1%	123	8.1%	127	8.4%	1%
	3	129	8.5%	49	3.5%	21%	129	8.5%	145	9.6%	4%
	4	249	16.5%	223	16.0%	1%	249	16.5%	247	16.3%	1%
	5	110	7.3%	81	5.8%	6%	110	7.3%	110	7.3%	0%
	6	118	7.8%	67	4.8%	12%	118	7.8%	110	7.3%	2%
	7	65	4.3%	74	5.3%	5%	65	4.3%	67	4.4%	0%
	8	117	7.7%	125	9.0%	5%	117	7.7%	110	7.2%	2%
	9	134	8.9%	155	11.2%	8%	134	8.9%	135	8.9%	0%
	10	105	7.0%	80	5.8%	5%	105	7.0%	111	7.3%	1%
	11	142	9.4%	218	15.7%	19%	142	9.4%	137	9.1%	1%
	12	40	2.6%	39	2.8%	1%	40	2.6%	37	2.4%	1%
	13	59	3.9%	68	4.9%	5%	59	3.9%	62	4.1%	1%
	14	32	2.1%	24	1.7%	3%	32	2.1%	32	2.1%	0%
Socio-economic status[d]	1	281	18.6%	285	20.5%	5%	281	18.6%	293	19.3%	2%
	2	328	21.7%	302	21.7%	0%	328	21.7%	336	22.2%	1%
	3	320	21.2%	299	21.5%	1%	320	21.2%	310	20.5%	2%

© 2023 Muanda FT et al. *JAMA Network Open.*

		Unweighted data (=2900) LD-MTX					Weighted data (n=3024)[b] LD-MTX				.
		15 to 35 mg/week (n=1510		5 to <15 mg/week (n=1390)		Standardized Difference[c]	15 to 35 mg/week (n=1510)		5 to <15 mg/week (n=1514)		Standardized Difference[c]
	4	288	19.1%	258	18.6%	1%	288	19.1%	291	19.2%	0%
	5	293	19.4%	246	17.7%	4%	293	19.4%	284	18.7%	2%
Prescriber information											
Rheumatologist		913	60.5%	627	45.1%	31%	913	60.5%	897	59.3%	2%
General practitioner		191	12.6%	237	17.1%	13%	191	12.6%	202	13.3%	2%
Dermatologist		128	8.5%	193	13.9%	17%	128	8.5%	124	8.2%	1%
Internist		99	6.6%	111	8.0%	5%	99	6.6%	108	7.1%	2%
Other		64	4.2%	94	6.8%	11%	64	4.2%	67	4.4%	1%
Missing		115	7.6%	128	9.2%	6%	115	7.6%	116	7.7%	0%
Comorbidities[e]											
Acute kidney injury		88	5.8%	116	8.3%	10%	88	5.8%	79	5.2%	3%
Alcoholism		17	1.1%	13	0.9%	2%	17	1.1%	14	0.9%	2%
Angina		261	17.3%	229	16.5%	2%	261	17.3%	277	18.3%	3%
Atrial fibrillation/flutter		94	6.2%	105	7.6%	6%	94	6.2%	113	7.5%	5%
Bipolar disorder		32	2.1%	21	1.5%	5%	32	2.1%	45	3.0%	6%
Chronic liver disease		49	3.2%	58	4.2%	5%	49	3.2%	52	3.4%	1%
Chronic obstructive pulmonary disease		461	30.5%	402	28.9%	4%	461	30.5%	487	32.2%	4%
Cirrhosis		30	2.0%	40	2.9%	6%	30	2.0%	27	1.8%	1%
Coronary artery disease (minus angina)		423	28.0%	397	28.6%	1%	423	28.0%	429	28.3%	1%
Dementia		139	9.2%	130	9.4%	1%	139	9.2%	140	9.2%	0%
Anemia		448	29.7%	416	29.9%	0%	448	29.7%	441	29.1%	1%

© 2023 Muanda FT et al. *JAMA Network Open.*

	Unweighted data (=2900) LD-MTX					Weighted data (n=3024)[b] LD-MTX				.
	15 to 35 mg/week (n=1510		5 to <15 mg/week (n=1390)		Standardized Difference[c]	15 to 35 mg/week (n=1510)		5 to <15 mg/week (n=1514)		Standardized Difference[c]
Glaucoma	176	11.7%	148	10.6%	3%	176	11.7%	162	10.7%	3%
Major hemorrhage	100	6.6%	84	6.0%	2%	100	6.6%	92	6.1%	2%
Congestive heart failure	238	15.8%	243	17.5%	5%	238	15.8%	237	15.6%	1%
Hypertension	1213	80.3%	1116	80.3%	0%	1213	80.3%	1231	81.3%	3%
Hypokalemia	31	2.1%	35	2.5%	3%	31	2.1%	27	1.8%	2%
Hyponatremia	22	1.5%	33	2.4%	7%	22	1.5%	21	1.4%	1%
Hypothyroidism	194	12.8%	200	14.4%	5%	194	12.8%	199	13.1%	1%
Migraine	40	2.6%	47	3.4%	5%	40	2.6%	40	2.7%	1%
Obesity	95	6.3%	75	5.4%	4%	95	6.3%	108	7.1%	3%
Parkinson disease	17	1.1%	28	2.0%	7%	17	1.1%	17	1.1%	0%
Peripheral vascular disease	19	1.3%	18	1.3%	0%	19	1.3%	27	1.8%	4%
Schizophrenia	23	1.5%	29	2.1%	5%	23	1.5%	21	1.4%	1%
Ischemic stroke	22	1.5%	25	1.8%	2%	22	1.5%	29	1.9%	3%
Depression	114	7.5%	110	7.9%	2%	114	7.5%	125	8.3%	3%
Rheumatoid arthritis	803	53.2%	697	50.1%	6%	803	53.2%	806	53.2%	0%
Syncope	28	1.9%	22	1.6%	2%	28	1.9%	27	1.8%	1%
Arrythmia	141	9.3%	141	10.1%	3%	141	9.3%	176	11.6%	8%
Inflammatory bowel disease	25	1.7%	26	1.9%	2%	25	1.7%	25	1.7%	0%
Major cancer[f]	611	40.5%	560	40.3%	0%	611	40.5%	599	39.5%	2%
Prostatic hyperplasia	181	12.0%	129	9.3%	9%	181	12.0%	200	13.2%	4%
Fracture	102	6.8%	111	8.0%	5%	102	6.8%	96	6.3%	2%
Falls	55	3.6%	86	6.2%	12%	55	3.6%	57	3.8%	1%
Hyperkaliemia	11	0.7%	15	1.1%	4%	11	0.7%	12	0.8%	1%

© 2023 Muanda FT et al. *JAMA Network Open.*

	Unweighted data (=2900) LD-MTX					Weighted data (n=3024)[b] LD-MTX				.
	15 to 35 mg/week (n=1510		5 to <15 mg/week (n=1390)		Standardized Difference[c]	15 to 35 mg/week (n=1510)		5 to <15 mg/week (n=1514)		Standardized Difference[c]
Prostatitis	34	2.3%	27	1.9%	3%	34	2.3%	35	2.3%	0%
Hypotension	31	2.1%	34	2.4%	2%	31	2.1%	33	2.2%	1%
Gallstones /biliary stones	74	4.9%	70	5.0%	0%	74	4.9%	73	4.8%	0%
Prior pneumotoxcity	69	4.6%	73	5.3%	3%	69	4.6%	66	4.4%	1%
Prior myelotoxicity	15	1.0%	21	1.5%	5%	15	1.0%	12	0.8%	2%
Prior sepsis	24	1.6%	25	1.8%	2%	24	1.6%	23	1.5%	1%
Prior methotrexate toxicity	90	6.0%	106	7.6%	6%	90	6.0%	86	5.7%	1%
Psoriasis	218	14.4%	230	16.5%	6%	218	14.4%	243	16.0%	4%
Dermatomyositis	85	5.6%	106	7.6%	8%	85	5.6%	74	4.9%	3%
Sarcoidosis	13	0.9%	17	1.2%	3%	13	0.9%	14	0.9%	0%
Systemic sclerosis or scleroderma	109	7.2%	112	8.1%	3%	109	7.2%	122	8.1%	3%
Systemic lupus erythematosus	123	8.1%	141	10.1%	7%	123	8.1%	111	7.3%	3%
Atopic dermatitis or eczema	436	28.9%	482	34.7%	12%	436	28.9%	456	30.1%	3%
Ulcerative colitis (UC)	28	1.9%	32	2.3%	3%	28	1.9%	32	2.1%	1%
Crohn disease	30	2.0%	27	1.9%	1%	30	2.0%	27	1.8%	1%
Acute urinary retention	27	1.8%	34	2.4%	4%	27	1.8%	22	1.4%	3%
Myocardial infarction	61	4.0%	46	3.3%	4%	61	4.0%	66	4.4%	2%
Dyslipidemia	375	24.8%	369	26.5%	4%	375	24.8%	387	25.6%	2%
Macula degeneration	71	4.7%	78	5.6%	4%	71	4.7%	64	4.3%	2%
Gastroesophageal reflux disease	383	25.4%	355	25.5%	0%	383	25.4%	383	25.3%	0%
Osteoarthritis	153	10.1%	131	9.4%	2%	153	10.1%	166	11.0%	3%

© 2023 Muanda FT et al. *JAMA Network Open.*

		Unweighted data (=2900)<br>LD-MTX					Weighted data (n=3024)[b]<br>LD-MTX				.
		15 to 35 mg/week (n=1510		5 to <15 mg/week (n=1390)		Standardized Difference[c]	15 to 35 mg/week (n=1510)		5 to <15 mg/week (n=1514)		Standardized Difference[c]
Major surgery		86	5.7%	85	6.1%	2%	86	5.7%	92	6.1%	2%
Prostate cancer		56	3.7%	51	3.7%	0%	56	3.7%	56	3.7%	0%
Diabetes		393	26.0%	339	24.4%	4%	393	26.0%	418	27.6%	4%
Urinary tract infection		78	5.2%	122	8.8%	14%	78	5.2%	84	5.5%	1%
Gout		215	14.2%	203	14.6%	1%	215	14.2%	212	14.0%	1%
Charlson comorbidity index[g]	Mean ± SD	2.47	1.16	2.58	1.3	9%	2.47	1.16	2.45	1.14	2%
	Median (IQR)	2	(2-2)	2	(2-2)	.	2	(2-2)	2	(2-2)	.
	2	1206	79.9%	1065	76.6%	8%	1206	79.9%	1215	80.2%	1%
	3+	304	20.1%	325	23.4%	8%	304	20.1%	299	19.8%	1%
Medication use [h]											
Alpha adrenergic blocking agents		44	2.9%	47	3.4%	3%	44	2.9%	45		
Anti-arrhythmic		25	1.7%	14	1.0%	6%	25	1.7%	27	1.8%	
Antibiotics		457	30.3%	404	29.1%	3%	457	30.3%	453	29.9%	
Ace inhibitor		449	29.7%	390	28.1%	4%	449	29.7%	459	30.3%	
Anticoagulants		200	13.2%	156	11.2%	6%	200	13.2%	226	<15%	
Anticonvulsants		125	8.3%	111	8.0%	1%	125	8.3%	131	8.7%	
Angiotensin II receptor blockers		495	32.8%	441	31.7%	2%	495	32.8%	512	33.8%	
Aspirin		26	1.7%	39	2.8%	7%	26	1.7%	28	1.9%	
Antiplatelet agents		93	6.2%	77	5.5%	3%	93	6.2%	85	5.6%	
Antifungals		23	1.5%	31	2.2%	5%	23	1.5%	26	1.7%	
Tricyclic antidepressant		225	<15%	191	13.7%	3%	225	<15%	234	15.5%	
Gastrointestinal drugs		78	5.2%	86	6.2%	4%	78	5.2%	83	5.5%	

© 2023 Muanda FT et al. *JAMA Network Open.*

	Unweighted data (=2900) LD-MTX					Weighted data (n=3024)[b] LD-MTX				.
	15 to 35 mg/week (n=1510		5 to <15 mg/week (n=1390)		Standardized Difference[c]	15 to 35 mg/week (n=1510)		5 to <15 mg/week (n=1514)		Standardized Difference[c]
Beta blockers	469	31.1%	450	32.4%	3%	469	31.1%	464	30.6%	1%
Bone Calcium regulators	21	1.4%	29	2.1%	5%	21	1.4%	17	1.1%	3%
Benzodiazepine	198	13.1%	187	13.5%	1%	198	13.1%	192	12.7%	1%
Bisphosphonates	338	22.4%	280	20.1%	6%	338	22.4%	327	21.6%	2%
Beta agonist	158	10.5%	140	10.1%	1%	158	10.5%	170	11.2%	2%
H2 blockers	57	3.8%	53	3.8%	0%	57	3.8%	58	3.8%	0%
Channel calcium blockers	501	33.2%	461	33.2%	0%	501	33.2%	483	31.9%	3%
Cholinesterase inhibitors	25	1.7%	21	1.5%	2%	25	1.7%	19	1.3%	3%
Glucocorticoid	946	62.6%	854	61.4%	2%	946	62.6%	961	63.5%	2%
Loop diuretics	234	15.5%	271	19.5%	11%	234	15.5%	237	15.6%	0%
Nitrates	62	4.1%	72	5.2%	5%	62	4.1%	73	4.8%	3%
Fibrates	31	2.1%	26	1.9%	1%	31	2.1%	33	2.2%	1%
NSAIDS (excluding ASA)	357	23.6%	277	19.9%	9%	357	23.6%	363	24.0%	1%
Insulin	109	7.2%	124	8.9%	6%	109	7.2%	118	7.8%	2%
Opioids	356	23.6%	317	22.8%	2%	356	23.6%	381	25.2%	4%
Over-active bladder medication	40	2.6%	33	2.4%	1%	40	2.6%	34	2.3%	2%
Potassium Sparing diuretics	89	5.9%	88	6.3%	2%	89	5.9%	88	5.8%	0%
Allopurinol	126	8.3%	138	9.9%	6%	126	8.3%	115	7.6%	3%
Anti-psychotics	27	1.8%	39	2.8%	7%	27	1.8%	23	1.5%	2%
Proton pump inhibitors	655	43.4%	604	43.5%	0%	655	43.4%	652	43.1%	1%
5 alpha reductases	98	6.5%	72	5.2%	6%	98	6.5%	102	6.8%	1%
Selective serotonin reuptake inhibitors	177	11.7%	148	10.6%	3%	177	11.7%	218	14.4%	8%
Statins	750	49.7%	689	49.6%	0%	750	49.7%	783	51.7%	4%

© 2023 Muanda FT et al. *JAMA Network Open.*

		Unweighted data (=2900) LD-MTX					Weighted data (n=3024)[b] LD-MTX				.
		15 to 35 mg/week (n=1510		5 to <15 mg/week (n=1390)		Standardized Difference[c]	15 to 35 mg/week (n=1510)		5 to <15 mg/week (n=1514)		Standardized Difference[c]
Thiazide diuretics		304	20.1%	265	19.1%	3%	304	20.1%	301	19.9%	1%
Oral antidiabetics		362	24.0%	280	20.1%	9%	362	24.0%	379	25.1%	3%
Number of unique drug names	Mean ± SD	8.33	4.37	8.39	4.52	1%	8.33	4.37	8.47	4.68	3%
	Median (IQR)	8	(5-11)	8	(5-11)	.	8	(5-11)	8	(5-11)	.
	0-4	245	17.6%	294	19.5%	5%	294	17.6%	251	16.6%	8%
	5-9	653	43.2%	649	46.7%	7%	653	43.2%	713	47.1%	8%
	10-14	434	28.7%	377	27.1%	4%	434	28.7%	421	27.8%	2%
	15-19	107	7.1%	83	6.0%	4%	107	7.1%	91	6.0%	4%
	20+	22	1.5%	36	2.6%	8%	22	1.5%	37	2.5%	7%
Number of unique dins	Mean ± SD	8.96	4.88	8.98	5.07	0%	8.96	4.88	9.14	5.34	4%
	Median (IQR)	8	(5-12)	8	(6-12)	.	8	(5-12)	9	(6-12)	.
	0-4	272	18.0%	228	16.4%	4%	272	18.0%	237	15.7%	6%
	5-9	606	40.1%	597	42.9%	6%	606	40.1%	637	42.1%	4%
	10-14	437	28.9%	404	29.1%	0%	437	28.9%	469	31.0%	5%
	15-19	146	9.7%	108	7.8%	7%	146	9.7%	113	7.5%	8%
	20+	49	3.2%	53	3.8%	3%	49	3.2%	58	3.8%	3%
Healthcare use[J]											
Primary care visits	Mean ± SD	10.31	9.01	10.63	9.54	3%	10.31	9.01	10.33	9.44	0%
	Median (IQR)	8	(5-13)	8	(5-13)	.	8	(5-13)	8	(5-13)	.
	0-4	335	22.2%	296	21.3%	2%	335	22.2%	330	21.8%	1%
	5-9	556	36.8%	509	36.6%	0%	556	36.8%	557	36.8%	0%
	10-14	319	21.1%	292	21.0%	0%	319	21.1%	325	21.4%	1%
	15-19	142	9.4%	132	9.5%	0%	142	9.4%	155	10.2%	3%
	20+	158	10.5%	161	11.6%	4%	158	10.5%	147	9.7%	3%

© 2023 Muanda FT et al. *JAMA Network Open.*

		Unweighted data (=2900) LD-MTX					Weighted data (n=3024)[b] LD-MTX				.
		15 to 35 mg/week (n=1510		5 to <15 mg/week (n=1390)		Standardized Difference[c]	15 to 35 mg/week (n=1510)		5 to <15 mg/week (n=1514)		Standardized Difference[c]
Hospitalizations	Mean ± SD	0.18	0.57	0.22	0.59	7%	0.18	0.57	0.19	59.0%	2%
	Median (IQR)	0	(0-0)	0	(0-0)	.	0	(0-0)	0	(0-0)	.
	0	1322	87.5%	1174	84.5%	9%	1322	87.5%	1312	86.7%	2%
	1	132	8.7%	157	11.3%	9%	132	8.7%	148	9.8%	4%
	2	32	2.1%	39	2.8%	5%	32	2.1%	31	2.0%	1%
	3+	24	1.6%	20	1.4%	2%	24	1.6%	23	1.5%	1%
Emergency departments visits	Mean ± SD	0.72	1.35	0.82	1.46	7%	0.72	1.35	0.75	1.37	2%
	Median (IQR)	0	(0-1)	0	(0-1)	.	0	(0-1)	0	(0-1)	.
	0	976	64.6%	834	60.0%	10%	976	64.6%	912	60.3%	9%
	1	276	18.3%	289	20.8%	6%	276	18.3%	341	22.5%	10%
	2	135	8.9%	133	9.6%	2%	135	8.9%	140	9.2%	1%
	3+	123	8.1%	134	9.6%	5%	123	8.1%	120	8.0%	0%
TSH test		1040	68.9%	961	69.1%	0%	1040	68.9%	1046	69.1%	0%
At home physician service		31	2.1%	41	2.9%	5%	31	2.1%	29	1.9%	1%
Bone mineral density test		214	14.2%	195	14.0%	1%	214	14.2%	194	12.8%	4%
Cardiac catheterization		22	1.5%	17	1.2%	3%	22	1.5%	19	1.2%	3%
Cardiac stress test		206	13.6%	191	13.7%	0%	206	13.6%	229	15.1%	4%
Carotid ultrasound		80	5.3%	76	5.5%	1%	80	5.3%	83	5.5%	1%
Chest-x-ray		712	47.2%	675	48.6%	3%	712	47.2%	703	46.4%	2%
Cataract surgery		80	5.3%	79	5.7%	2%	80	5.3%	73	4.8%	2%
Cervical cancer screening		60	4.0%	44	3.2%	4%	60	4.0%	55	3.6%	2%
Colorectal cancer screening		230	15.2%	211	15.2%	0%	230	15.2%	248	16.4%	3%
Cholesterol test (total cholesterol, HDL)		993	65.8%	896	64.5%	3%	993	65.8%	1027	67.8%	4%

© 2023 Muanda FT et al. *JAMA Network Open.*

		Unweighted data (=2900) LD-MTX					Weighted data (n=3024)[b] LD-MTX				.
		15 to 35 mg/week (n=1510		5 to <15 mg/week (n=1390)		Standardized Difference[c]	15 to 35 mg/week (n=1510)		5 to <15 mg/week (n=1514)		Standardized Difference[c]
CT abdomen		173	11.5%	164	11.8%	1%	173	11.5%	183	12.1%	2%
CT extremities		22	1.5%	9	0.6%	9%	22	1.5%	19	1.3%	2%
CT head		146	9.7%	138	9.9%	1%	146	9.7%	150	9.9%	1%
CT neck		21	1.4%	21	1.5%	1%	21	1.4%	17	1.1%	3%
CT pelvis		161	10.7%	155	11.2%	2%	161	10.7%	168	11.1%	1%
CT spine		30	2.0%	23	1.7%	2%	30	2.0%	29	1.9%	1%
CT thorax		151	10.0%	184	13.2%	10%	151	10.0%	147	9.7%	1%
Echocardiography		357	23.6%	359	25.8%	5%	357	23.6%	352	23.2%	1%
Flu shot		704	46.6%	735	52.9%	13%	704	46.6%	736	48.6%	4%
Cystoscopy		56	3.7%	67	4.8%	5%	56	3.7%	65	4.3%	3%
Hearing test		62	4.1%	60	4.3%	1%	62	4.1%	58	3.9%	1%
Mammography		192	12.7%	131	9.4%	11%	192	12.7%	169	11.1%	5%
Prostate specific antigen (PSA) test		26	1.7%	15	1.1%	5%	26	1.7%	26	1.7%	0%
Holter monitoring		128	8.5%	121	8.7%	1%	128	8.5%	125	8.2%	1%
Parathyroid hormone testing		173	11.5%	154	11.1%	1%	173	11.5%	164	10.8%	2%
Pulmonary function test		188	12.5%	178	12.8%	1%	188	12.5%	207	13.7%	4%
Urinalysis		858	56.8%	804	57.8%	2%	858	56.8%	845	55.8%	2%
**Laboratory measurement**											
eGFR[k]	Mean ± SD	49.42	8.46	47.42	9.72	22%	49.42	8.46	49.45	9	0%
	Median (IQR)	51	(45-56)	49	(41-55)	.	51	(45-56)	52	(45-56)	.
Baseline eGFR categories	45-<60	1115	73.8%	903	65.0%	19%	1115	73.8%	1121	74.1%	1%

© 2023 Muanda FT et al. *JAMA Network Open.*

		Unweighted data (=2900) LD-MTX					Weighted data (n=3024)[b] LD-MTX				.
		15 to 35 mg/week (n=1510		5 to <15 mg/week (n=1390)		Standardized Difference[c]	15 to 35 mg/week (n=1510)		5 to <15 mg/week (n=1514)		Standardized Difference[c]
	30-<45	342	22.6%	404	29.1%	15%	342	22.6%	342	22.6%	0%
	<30	53	3.5%	83	6.0%	12%	53	3.5%	51	3.4%	1%
Urine ACR available		578	38.3%	495	35.6%	6%	578	38.3%	572	37.8%	1%
	Missing	932	61.7%	895	64.4%	6%	932	61.7%	941	62.2%	1%
Baseline ACR categories, µg/mg	<3	1289	85.4%	1188	85.5%	0%	1289	85.4%	1290	85.2%	1%
	3-30	172	11.4%	151	10.9%	2%	172	11.4%	176	11.6%	1%
	>30	49	3.2%	51	3.7%	3%	49	3.2%	48	3.1%	1%

Abbreviations: LD-MTX, low-dose methotrexate; ACE inhibitor, angiotensin-converting-enzyme inhibitor; H2 blockers, Histamine H2-receptor antagonists; eGFR, estimated glomerular filtration rate; IQR, interquartile range; LHIN, Local Health Integration Network; ACR, urine albumin-to-creatinine ratio.

[a] Unless otherwise specified in the footnotes, baseline characteristics were assessed on the date the patient filled a low-dose methotrexate prescription at 15 to 35 mg/week or 5 to <15 mg/week—the cohort entry date.

[b] Propensity score matching technique was used to balance comparison groups on indicators of baseline health, including all known indications for methotrexate use (including off-label indications). The propensity score was estimated using multivariable logistic regression with 140 covariates chosen *a priori* (defined in eTable 8 in the Supplement). We use greedy matching, to match low-dose methotrexate drug user (1:1) to hydroxychloroquine user based on the logit of the propensity score (within a caliper of ±0.2 standard deviations).[24]

[c] the difference between the groups divided by the pooled SD; a value greater than 10% is interpreted as a meaningful difference.[25]

[d] Income was categorized into fifths of average neighborhood income on the cohort entry date.

[e] Baseline comorbidities were assessed in the 5-year period before the cohort entry date.

© 2023 Muanda FT et al. *JAMA Network Open.*

[f] Cancer includes the following types of cancer: skin, mouth (lip, tonsil, etc), throat, stomach, small/large intestine, liver, gall bladder, pancreas, breast, male/female reproductive organs, heart, lung, bone, urinary system (kidney, bladder, etc), endocrine glands, as well as leukemias and lymphomas

[g] Presence of kidney disease is a variable in the Charlson comorbidity index, which automatically results in all individuals receiving a minimum score of 2

[h] Medication use was examined in the 120-day period before the cohort entry date (the Ontario Drug Benefit program dispenses a maximum 100-day supply).

[i] Glucocorticoids included many medications regardless of their route of administration such as hydrocortisone acetate, dexamethasone, beclomethasone dipropionate, prednisone, hydrocortisone, flumetasone pivalate, clioquinol, betamethasone valerate, betamethasone, triamcinolone acetonide, triamcinolone diacetate, triamcinolone, flurandrenolide, betamethasone & dexamethasone sodium phosphate, cortisone acetate, dexamethasone tebutate, prednisolone, dexamethasone, corticotrophin, prednisolone acetate, fluocinolone acetonide, hydrocortisone sodium succinate, methylprednisolone sodium succinate, methylprednisolone acetate, methylprednisolone disodium phosphate, methylprednisolone, fluocinonide, betamethasone disodium phosphate, medrysone & polyvinyl alcohol, prednisolone acetate & sulfacetamide sodium, dexamethasone & neomycin sulfate & polymyxin b sulfate, clioquinol & flumetasone pivalate, clioquinol & hydrocortisone, 1,2-propanediol diacetate & acetic acid & benzethonium chloride & hydrocortisone, clioquinol & triamcinolone acetonide, flurandrenolide, fluocinolone acetonide, dexamethasone & neomycin sulfate, hydrocortisone & lidocaine hcl & neomycin sulfate, haemorroidal veinous plexus, prednisone & pheniramine maleate & inositol & phosphatidyl choline & vitamin a & vitamin d2& vitamin e, chloramphenicol & hydrocortisone acetate, haemorroidal veinous plexus, dexamethasone & framycetin sulfate & gramicidin, dibucaine hcl & esculin & framycetin sulfate & hydrocortisone, betamethasone valerate & neomycin sulfate, betamethasone valerate & gentamicin sulfate, prednisolone acetate & sulfacetamide sodium, ascorbic acid & chlorpheniramine maleate & prednisone acetate, neomycin sulfate & prednisolone acetate & sulfacetamide sodium, gramicidin & neomycin sulfate & triamcinolone acetonide, methylprednisolone, acetylsalicylic acid & methyltestosterone, methylprednisolone sulfate & neomycin sulfate, hydrocortisone acetate & neomycin sulfate, aluminum chlorohydrate & methylprednisolone acetate & neomycin sulfate & sulfur, gramicidin & neomycin sulfate & nystatin & triamcinolone acetonide, hydrocortisone acetate & zinc oxide, hydrocortisone acetate & pramoxine hcl & zinc sulfate, aluminum chlorohydrate & methylprednisolone acetate & sulfur, hydrocortisone acetate & zinc oxide, hydrocortisone acetate & pramoxine hcl & zinc sulfate, desonide, clobetasol propionate, beclomethasone dipropionate & clioquinol, bacitracin zinc & hydrocortisone & neomycin sulfate & polymyxin b sulfate, hydrocortisone & neomycin sulfate & polymyxin b sulfate, gramicidin & neomycin sulfate & nystatin & triamcinolone acetonide, fluorometholone & polyvinyl alcohol, aluminum chlorohydrate & methylprednisolone acetate & sulfur, fluorometholone, lidocaine hcl & methylprednisolone acetate, flumetasone pivalate & salicylic acid, fluorometholone, lidocaine hcl & methylprednisolone acetate, aclometasone dipropionate, allantoin & chloramphenicol & hydrocortisone, amcinonide, atropine sulfate & prednisolone acetate, bacitracin & hydrocortisone & neomycin sulfate & polymyxin b sulfate, benzalkonium &

© 2023 Muanda FT et al. *JAMA Network Open.*

dexamethasone & tobramycin , benzocaine & hydrocortisone acetate & zinc sulfate, betamethasone & sulfacetamide sodium, betamethasone acetate & betamethasone sodium phosphate, betamethasone benzoate, betamethasone dipropionate, betamethasone dipropionate &calcipotriene, betamethasone dipropionate &clotrimazole, betamethasone dipropionate & gentamicin sulfate, betamethasone dipropionate & salicylic acid, betamethasone disodium phosphate, betamethasone valerate & salicylic acid, betamethasone valerate & gentamycin sulfate, betamethasone valerate & neomycin sulfate, budesonide, camphor & hydrocortisone & menthol, chlorbutol & dexamethasone &tobramycin, ciclesonide.

[j] Total number of healthcare visits/tests in the 12-month period before the cohort entry date.

[k] The most recent eGFR measurement in the 365-day period before the cohort entry date (including the cohort entry date); eGFR was calculated using the new Chronic Kidney Disease (CKD)–Epidemiology (EPI) equation: $142\times \min([\text{serum creatinine concentration in } \mu\text{mol/L}/88.4]/\kappa, 1)^{\alpha} \times \max([\text{serum creatinine concentration in } \mu\text{mol/L}/88.4]/\kappa, 1)^{-1.200} \times 0.9938^{Age} \times 1.012$ [if female]; κ=0.7 if female and 0.9 if male; α=-0.241 if female and -0.302 if male; min=the minimum of serum creatinine concentration/κ or 1; max=the maximum of serum creatinine concentration/κ or 1.

© 2023 Muanda FT et al. *JAMA Network Open.*

eTable 24. Risk of an outpatient or hospital visit with hearing disorders in older adults with chronic kidney disease within 90 days of starting a new prescription for low-dose methotrexate vs a new prescription for hydroxychloroquine[a]

	Matched[b]				
	No. events (%)				
	LD-MTX (n = 2309)	HCQ (n = 2309)	Risk difference, % (95% CI)	NNH, %	Risk ratio (95% CI)
**Primary outcome**					
Outpatient or hospital visit with hearing disorders	26 (1.13)	33 (1.43)	-0.30 (-0.96 to 0.35)	NA	0.79 (0.47 to 1.32)

Abbreviations: LD-MTX, low-dose methotrexate; HCQ, hydroxychloroquine; NA, not applicable.

[a] Reference group: hydroxychloroquine.

[b] The propensity score was estimated using multivariable logistic regression with 140 covariates chosen *a priori* (defined in eTable 8 in the Supplement). We use greedy matching, to match low-dose methotrexate drug user (1:1) to hydroxychloroquine user based on the logit of the propensity score (within a caliper of ±0.2 standard deviations).[24] Risk ratios and 95% CIs were obtained using modified Poisson regression[26]and weighted risk differences and 95% CIs were obtained using a binomial regression model with an identity link function.

[c] The 90-day risk of a hospital visit with major surgery.

© 2023 Muanda FT et al. *JAMA Network Open.*

eTable 25. Proportion of patients who received a test for a complete blood count, liver function, or a chest radiograph within 90 days of starting a new prescription for low-dose methotrexate vs. a new prescription for hydroxychloroquine

Tests	**Matched cohort**		**Risk Ratio, % (95% CI)**
	**No. events (%)**	**No. events (%)**	
	**LD-MTX**	**HCQ**	
	**(n = 2900)**	**(n = 2900)**	
Complete blood count tests	1890 (81.9)	1313 (56.9)	1.44 (1.38 to1.50)
Liver function tests (ASAT AND ALAT)	1832 (79.3)	1055 (45.7)	1.74 (1.65 to 1.82)
Chest X-ray	504 (21.8)	441 (19.1)	1.14 (1.02 to 1.28)

Abbreviations: ASAT, aspartate amino transferase; ALAT, alanine amino transferase.

© 2023 Muanda FT et al. *JAMA Network Open.*

eTable 26. Risk of a hospital visit with myelosuppression, sepsis, pneumotoxicity, and hepatotoxicity in older adults with chronic kidney disease who received at least one test within 90 days of starting a new prescription for low-dose methotrexate vs a new prescription for hydroxychloroquine[a]

	**Unweighted**		**Weighted[b]**				
	**No. events (%)**		**No. events (%)**				
	**LD-MTX (n = 2001)**	**HCQ (n = 1502)**	**LD-MTX (n = 2001)**	**HCQ (n = 1999)**	**Risk difference, % (95% CI)**	**NNH, % (95% CI)**	**Risk ratio (95% CI)**
**Primary outcome**							
Hospital visit with myelosuppression, sepsis, pneumotoxicity, and hepatotoxicity[c]	82 (4.10)	39 (2.60)	82 (4.10)	48 (2.38)	1.72 (0.54 to 2.90)	58 (35 to 185)	1.72 (1.16 to 2.56)

Abbreviations: LD-MTX, low-dose methotrexate; HCQ, hydroxychloroquine

[a] Reference group: hydroxychloroquine

[b] The propensity score was estimated using multivariable logistic regression with 140 covariates chosen a priori (defined in eTable 8 in the Supplement). Patients in the reference group were weighted as [propensity score/(1 – propensity score)]. [26-29]This method produces a weighted pseudo-sample of patients in the reference group with the same distribution of measured covariates as the exposed group.[26,27] Weighted risk ratios and 95% CIs were obtained using modified Poisson regression[26]and weighted risk differences and 95% CIs were obtained using a binomial regression model with an identity link function.
[c] The 90-day risk of a hospital visit with myelosuppression, sepsis, pneumotoxicity, and hepatotoxicity**.**

© 2023 Muanda FT et al. *JAMA Network Open.*

eTable 27. Risk of a hospital admission with myelosuppression, sepsis, pneumotoxicity, and hepatotoxicity in older adults with chronic kidney disease within 90 days of starting a new prescription for low-dose methotrexate vs a new prescription for hydroxychloroquine[a]

	Matched[b]				
	No. events (%)				
	LD-MTX (n = 2309)	HCQ (n = 2309)	Risk difference, % (95% CI)	NNH, % (95% CI)	Risk ratio (95% CI)
**Primary outcome**					
Hospital admission with myelosuppression, sepsis, pneumotoxicity, and hepatotoxicity[c]	30 (1.30)	11 (0.48)	0.82 (0.28 to 1.37)	122 (73 to 357)	2.72 (1.37 to 5.44)

Abbreviations: LD-MTX, low-dose methotrexate; HCQ, hydroxychloroquine.

[a] Reference group: hydroxychloroquine.

[b] The propensity score was estimated using multivariable logistic regression with 140 covariates chosen *a priori* (defined in eTable 8 in the Supplement). We use greedy matching to match low-dose methotrexate drug user (1:1) to hydroxychloroquine user based on the logit of the propensity score (within a caliper of ±0.2 standard deviations).[24] Risk ratios and 95% CIs were obtained using modified Poisson regression[26] and weighted risk differences, and 95% CIs were obtained using a binomial regression model with an identity link function.

[c] The 90-day risk of a hospital visit with myelosuppression, sepsis, pneumotoxicity, and hepatotoxicity.

© 2023 Muanda FT et al. *JAMA Network Open.*

eTable 28. Risk of a hospital visit with myelosuppression, sepsis, pneumotoxicity, and hepatotoxicity in older adults with chronic kidney disease within 30 days of starting a new prescription for low-dose methotrexate vs. a new prescription for hydroxychloroquine[a]

	Unmatched		Matched[b]				
	No. events (%)		No. events (%)				
	LD-MTX (n = 2900)	HCQ (n = 4009)	LD-MTX (n = 2309)	HCQ (n = 2309)	Risk difference, % (95% CI)	NNH, % (95% CI)	Risk ratio (95% CI)
**Primary outcome**							
Hospital visit with myelosuppression, sepsis, pneumotoxicity, and hepatotoxicity[c]	73 (2.52)	46 (1.15)	61 (2.64)	24 (1.04)	1.60 (0.85 to 2.35)	63 (43 to 118)	2.54 (1.61 to 4.01)

Abbreviations: LD-MTX, low-dose methotrexate; HCQ, hydroxychloroquine.

[a] Reference group: hydroxychloroquine.

[b] The propensity score was estimated using multivariable logistic regression with 140 covariates chosen *a priori* (defined in eTable 8 in the Supplement). We use greedy matching, to match low-dose methotrexate drug user (1:1) to hydroxychloroquine user based on the logit of the propensity score (within a caliper of ±0.2 standard deviations).[24] Risk ratios and 95% CIs were obtained using modified Poisson regression[26] and weighted risk differences, and 95% CIs were obtained using a binomial regression model with an identity link function.

[c] The 30-day risk of a hospital visit with myelosuppression, sepsis, pneumotoxicity, and hepatotoxicity.

© 2023 Muanda FT et al. *JAMA Network Open.*

eFigure 1. Study design diagram comparing use of methotrexate vs hydroxychloroquine and the risk of serious adverse events in patients with chronic kidney disease

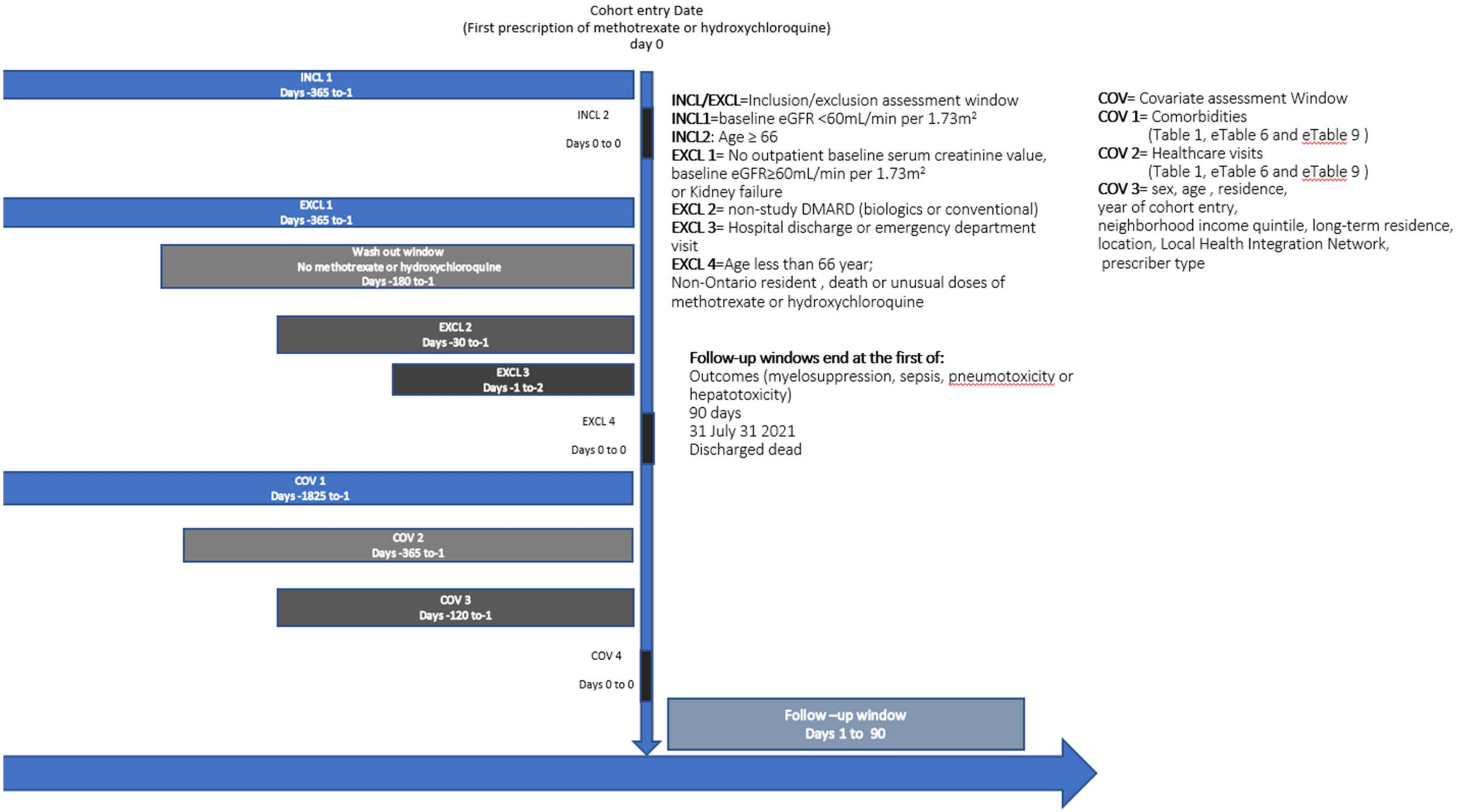

Abbreviations: DMARD, disease-modifying antirheumatic drug; LD-MTX, low-dose methotrexate; HCQ, hydroxychloroquine

© 2023 Muanda FT et al. *JAMA Network Open.*

eFigure 2. E-value analysis to assess the extent of unmeasured confounding that would be required to negate the observed results

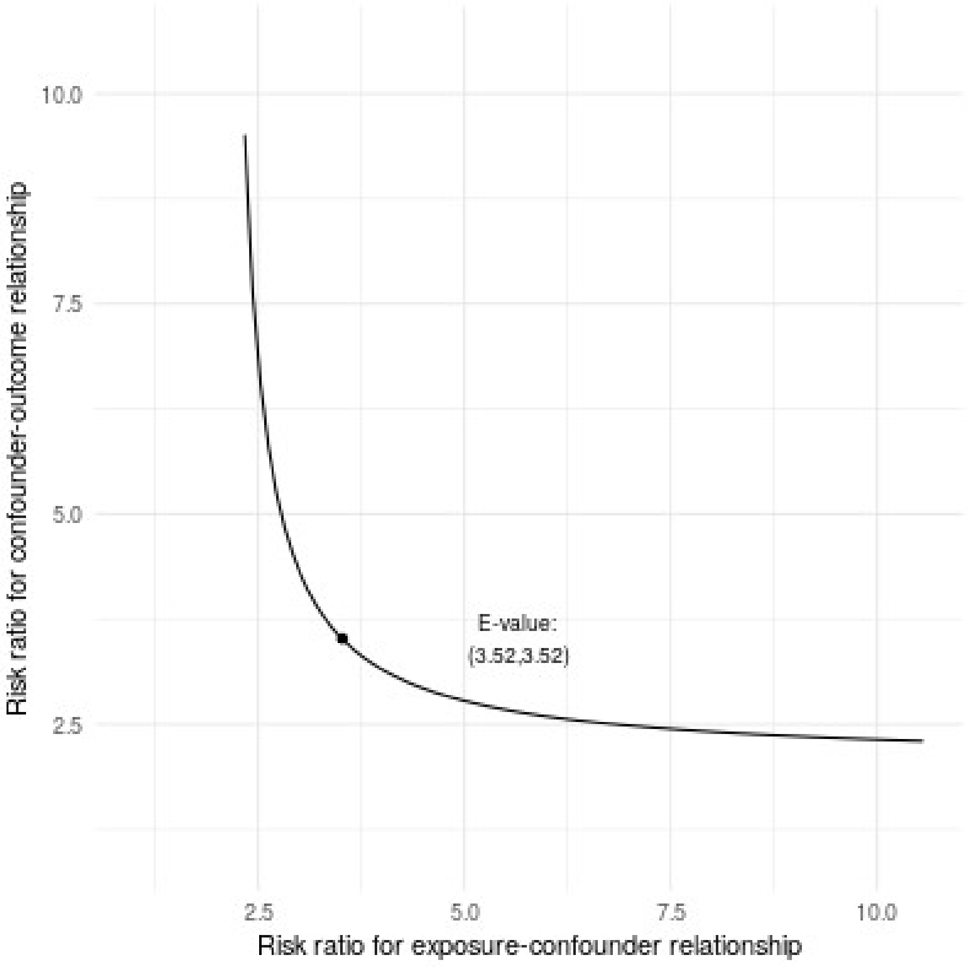

E-value for point estimate: 3.52 and for lower bound of the confidence interval: 2.19

© 2023 Muanda FT et al. *JAMA Network Open.*

eReferences

1. Methotrexate: Drug information – UpToDate. https://www.uptodate.com/contents/methotrexate-drug-information?source=auto_suggest&selectedTitle=1~2---1~2---methot&search=methotrexate. Accessed November 04, 2022.

2. Methotrexate (Product Monograph). https://pdf.hres.ca/dpd_pm/00064334.PDF. Accessed November 04, 2022.

3. Kintzel PE, Dorr RT. Anticancer drug renal toxicity and elimination: dosing guidelines for altered renal function. Cancer Treat Rev. 1995;21(1):33-64. [PubMed 7859226]

4. Aronoff GR, Bennett WM, Berns JS, et al. Drug Prescribing in Renal Failure: Dosing Guidelines for Adults and Children. 5th ed. Philadelphia, PA: American College of Physicians; 2007, p 101.

5. Bressolle F, Bologna C, Kinowski JM, Sany J, Combe B. Effects of moderate renal insufficiency on pharmacokinetics of methotrexate in rheumatoid arthritis patients. Ann Rheum Dis. Feb 1998;57(2):110-3. doi:10.1136/ard.57.2.110

6. Gutierrez-Ureña S, Molina JF, García CO, Cuéllar ML, Espinoza LRJA, Rheumatology ROJotACo. Pancytopenia secondary to methotrexate therapy in rheumatoid arthritis. 1996;39(2):272-6.

7. Calvo-Romero JMJAoP. Severe pancytopenia associated with low-dose methotrexate therapy for rheumatoid arthritis. 2001;35(12):1575-7.

8. Arakawa Y, Arakawa A, Vural S, Mahajan R, Prinz JCJJcr. Renal clearance and intracellular half-life essentially determine methotrexate toxicity: a case series. 2019;5(1):98.

9. Yoon KH, Ng SCJJJoCR. Early onset methotrexate-induced pancytopenia and response to G-CSF: a report of two cases. 2001;7(1):17-20.

10. Shaikh N, Sardar M, Raj R, Jariwala PJCrim. A rapidly fatal case of low-dose methotrexate toxicity. 2018;2018.

11. Park GT, Jeon DW, Roh KH, Mun HS, Lee CH, Park CH, et al. A case of pancytopenia secondary to low-dose pulse methotrexate therapy in a patient with rheumatoid arthritis and renal insufficiency. 1999;14(1):85.

12. Chen T-J, Chung W-H, Chen C-B, Hui RC-Y, Huang Y-H, Lu Y-T, et al. Methotrexate-induced epidermal necrosis: a case series of 24 patients. 2017;77(2):247-55. e2.

13. Lim A, Gaffney K, Scott DJR. Methotrexate-induced pancytopenia: serious and under-reported? Our experience of 25 cases in 5 years. 2005;44(8):1051-5.

© 2023 Muanda FT et al. *JAMA Network Open.*

14. Ajmani S, Preet Singh Y, Prasad S, Chowdhury A, Aggarwal A, Lawrence A, Misra R, Mishra R, Agarwal V. Methotrexate-induced pancytopenia: a case series of 46 patients. Int J Rheum Dis. 2017 Jul;20(7):846-851. doi: 10.1111/1756-185X.13004. Epub 2017 Mar 5. PMID: 28261918

15. Bergner R, Wadsack D, Löffler C (2019) Severe MTX Toxicity in Rheumatic Diseases - Analysis of 22 Cases. J Rheum Dis Treat 5:070. doi.org/10.23937/2469-5726/1510070

16. Mitsuboshi SJBJoCP. Risk of haematological events and preventive effect of folic acid in methotrexate users with chronic kidney disease and rheumatoid arthritis: Analysis of the Japanese Adverse Drug Event Report database. 2021;87(5):2286-9.

17. . Lee JS, Oh JS, Kim YG, Lee CK, Yoo B, Hong S. Methotrexate-related toxicity in patients with rheumatoid arthritis and renal dysfunction. *Rheumatol Int*. May 2020;40(5):765-770. doi:10.1007/s00296-020-04547-

18. Kuklik N, Stausberg J, Jöckel KH. Adverse drug events in German hospital routine data: A validation of International Classification of Diseases, 10th revision (ICD-10) diagnostic codes. PLoS One. 2017 Nov 2;12(11):e0187510. doi: 10.1371/journal.pone.0187510. PMID: 29095926; PMCID: PMC5667751.

19. Heden KE, Jensen AØ, Farkas DK, Nørgaard M. Validity of a procedure to identify patients with chronic idiopathic thrombocytopenic purpura in the Danish National Registry of Patients. Clin Epidemiol. 2009 Aug 9;1:7-10. doi: 10.2147/clep.s4832. PMID: 20865080; PMCID: PMC2943173.

20. Skull SA, Andrews RM, Byrnes GB, Campbell DA, Nolan TM, Brown GV, Kelly HA. ICD-10 codes are a valid tool for identification of pneumonia in hospitalized patients aged > or = 65 years. Epidemiol Infect. 2008 Feb;136(2):232-40. doi: 10.1017/S0950268807008564. Epub 2007 Apr 20. PMID: 17445319; PMCID: PMC2870806.

21. Myers RP, Leung Y, Shaheen AA, Li B. Validation of ICD-9-CM/ICD-10 coding algorithms for the identification of patients with acetaminophen overdose and hepatotoxicity using administrative data. BMC Health Serv Res. 2007 Oct 2;7:159. doi: 10.1186/1472-6963-7-159. PMID: 17910762; PMCID: PMC2174469.

22. Jolley RJ, Quan H, Jetté N, Sawka KJ, Diep L, Goliath J, Roberts DJ, Yipp BG, Doig CJ. Validation and optimisation of an ICD-10-coded case definition for sepsis using administrative health data. BMJ Open. 2015 Dec 23;5(12):e009487. doi: 10.1136/bmjopen-2015-009487. PMID: 26700284; PMCID: PMC4691777.

23. Jha P, Deboer D, Sykora K, Naylor CD: Characteristics and mortality outcomes of thrombolysis trial participants and nonparticipants: A population-based comparison. J Am Coll Cardiol 27: 1335– 1342, 1996.

24. Austin PC. Statistical criteria for selecting the optimal number of untreated subjects matched to each treated subject when using many-to-one matching on the propensity

© 2023 Muanda FT et al. *JAMA Network Open.*

score. *American journal of epidemiology*. Nov 1 2010;172(9):1092-7. doi:10.1093/aje/kwq224

25. Austin PC, Grootendorst P, Anderson GM. A comparison of the ability of different propensity score models to balance measured variables between treated and untreated subjects: a Monte Carlo study. Stat Med. 2007;26(4):734-753.

26. Zou G. A modified poisson regression approach to prospective studies with binary data. *American journal of epidemiology.* 2004;159(7):702-70

27. Sato T, Matsuyama Y. Marginal structural models as a tool for standardization. *Epidemiology (Cambridge, Mass.).* Nov 2003;14(6):680-686.

28. Brookhart MA, Wyss R, Layton JB, Sturmer T. Propensity score methods for confounding control in nonexperimental research. *Circ Cardiovasc Qual Outcomes.* Sep 1 2013;6(5):604-611.

29. Austin PC. An Introduction to Propensity Score Methods for Reducing the Effects of Confounding in Observational Studies. *Multivariate Behav Res.* May 2011;46(3):399-424.

30. Desai RJ, Franklin JM. Alternative approaches for confounding adjustment in observational studies using weighting based on the propensity score: a primer for practitioners. BMJ (Clinical research ed). 2019;367: l5657.

31. Desai RJ, Rothman KJ, Bateman BT, Hernandez-Diaz S, Huybrechts KF. A propensity-score-based fine stratification approach for confounding adjustment when exposure is infrequent. Epidemiology 2017; 28:249-57. doi:10.1097/ EDE.0000000000000595
