# Supplementary material for: Low-Dose Methotrexate and Serious Adverse Events Among Older Adults With Chronic Kidney Disease
Source: JAMA Netw Open. 2023 Nov 27;6(11):e2345132. doi: 10.1001/jamanetworkopen.2023.45132 (PMC10682837; doi:10.1001/jamanetworkopen.2023.45132)
Supplement: Supplement 2. — Data Sharing Statement [file jamanetwopen-e2345132-s002.pdf]

Data Sharing Statement

Muanda. Low-Dose Methotrexate and Serious Adverse Events Among Older Adults With Chronic Kidney Disease. *JAMA Netw Open*. Published November 27, 2023. doi:10.1001/jamanetworkopen.2023.45132

Data
Data available: No

Additional Information
Explanation for why data not available: No Data will be shared because of governmental privacy regulations.
